# Zintl cluster supported low coordinate Rh(i) centers for catalytic H/D exchange between H_2_ and D_2_†

**DOI:** 10.1039/d2sc02552c

**Published:** 2022-06-09

**Authors:** Oliver P. E. Townrow, Simon B. Duckett, Andrew S. Weller, Jose M. Goicoechea

**Affiliations:** Department of Chemistry, University of Oxford, Chemistry Research Laboratory 12 Mansfield Road Oxford OX1 3TA UK jose.goicoechea@chem.ox.ac.uk; Department of Chemistry, University of York YO10 5DD UK andrew.weller@york.ac.uk

## Abstract

Ligand exchange reactions of [Rh(COD){η^4^-Ge_9_(Hyp)_3_}] with L-type nucleophiles such as PMe_3_, PPh_3_, IMe_4_ (IMe_4_ = 1,3,4,5-tetramethylimidazol-2-ylidene) or [W(Cp)_2_H_2_] result in the displacement of the COD ligand to afford clusters with coordinatively unsaturated trigonal pyramidal rhodium(i) centers [Rh(L){η^3^-Ge_9_(Hyp)_3_}]. These species can be readily protonated allowing access to cationic rhodium–hydride complexes, *e.g.* [RhH(PPh_3_){η^3^-Ge_9_(Hyp)_3_}]^+^. These clusters act as catalysts in H/D exchange between H_2_ and D_2_ and alkene isomerisation, thereby illustrating that metal-functionalized Zintl clusters are active in both H–H and C–H bond activation processes. The mechanism of H/D exchange was probed using *para*hydrogen induced polarization experiments.

## Abstract 2

We describe the synthesis of the coordinatively unsaturated Zintl clusters [Rh(L){η^3^-Ge_9_(Hyp)_3_}] (where L = PMe_3_, PPh_3_, IMe_4_ or [W(Cp)_2_H_2_]). These species are active catalysts in H/D exchange and C–H bond activation reactions.

## Graphical abstract

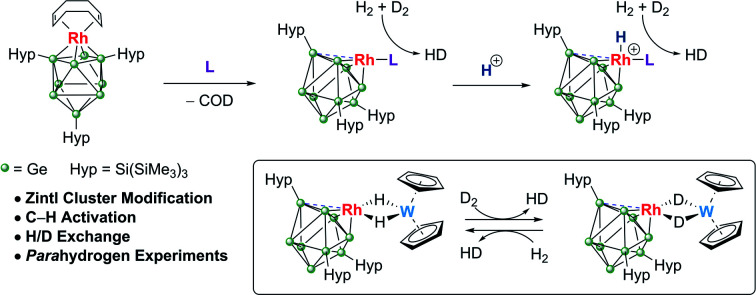

## Introduction

Transition-metal/main-group element alloys (TMMGAs) are an interesting class of materials that combine late transition metals (*e.g.* nickel, platinum or palladium) with low melting point post-transition metals such as gallium, indium, tin, lead or bismuth. In their molten state, these alloys have been employed as catalysts in a number of challenging processes that involve light alkane valorization, *e.g.* methane pyrolysis to form graphitic carbon and hydrogen, dry reforming of methane, or propane dehydrogenation.^1–7^ These melts offer access to “solvated” late transition metal atoms accounting for their impressive catalytic performance, however *in situ* monitoring of reaction profiles/mechanisms remains a challenge due to the harsh conditions employed (*e.g.* operating temperatures of 1040 °C) and the lack of suitable spectroscopic probes. This led us to turn our attention to the synthesis of molecular TMMGAs, compounds in which a late transition-metal–ligand fragment is supported on a main-group cluster platform. For this purpose, we have explored the use of Zintl clusters, anionic clusters of the main-group elements, that can be readily functionalized with transition-metals. In principle, these compounds can act as molecular models for TMMGAs. However, poor solubility in non-polar solvents, low yielding syntheses, and a tendency for oxidative coupling have long hindered their application in homogeneous catalysis.

The silylated Zintl ion [Ge_9_(Hyp)_3_]^−^ (Hyp = Si(SiMe_3_)_3_) is an attractive platform for the design of molecular TMMGAs due to its increased solubility in hydrocarbon solvents.^8,9^ Recently we demonstrated that the rhodium(i) cluster [Rh(COD){η^4^-Ge_9_(Hyp)_3_}] can be employed as a catalyst for the hydrogenation of cyclic alkenes.^10^ This is the first example of a Zintl cluster being used in catalysis, and a proof-of-concept study illustrating that this class of compound may be used to mimic the impressive reactivity of TMMGAs. Furthermore, the [Ge_9_(Hyp)_3_]^−^ cage can readily isomerize to adapt to the steric and electronic requirements of the transition metal.^10^ These molecular dynamics have the potential to play a pivotal role in catalysis, where geometric responses to substrate and product binding can provide lower energy pathways in elementary catalytic steps. Herein we demonstrate that, in addition to being active hydrogenation catalysts, metal-functionalized Zintl clusters are also active catalysts for H/D exchange between H_2_ and D_2_, and alkene isomerization. While both of these catalytic bond transformations are common in organometallic chemistry they are unknown in Zintl ion chemistry.^11^

## Results and discussion

**Scheme 1:**
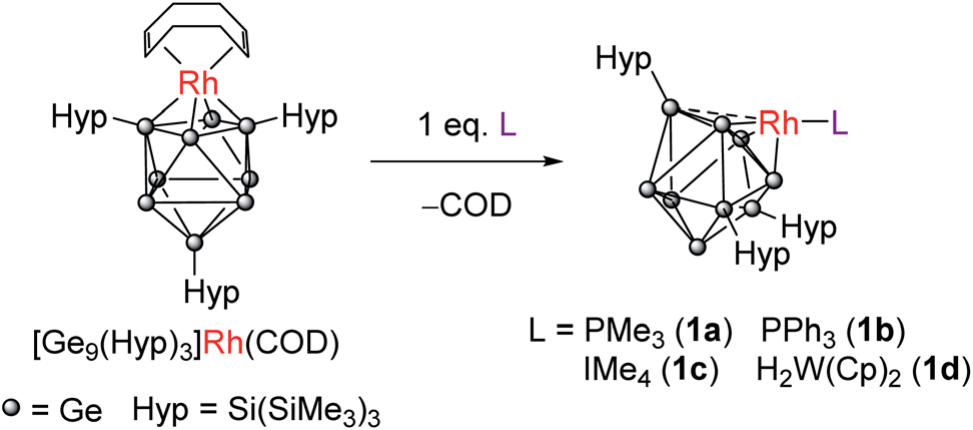
Synthesis of 1a–1d from reaction of [Rh(COD){η^4^-Ge_9_(Hyp)_3_}] with Lewis bases.

We have previously shown that reaction of [Rh(COD){η^4^-Ge_9_(Hyp)_3_}] with the bidentate, κ^2^, ligand dppe (1,2-bis(diphenylphosphino)ethane) results in both the displacement of the COD ligand and cluster isomerization, so that the rhodium centre moves from occupying at a 4-connected vertex position in the cluster to a 5-connected vertex position. The rhodium(i) center is formally 18-electron in both.^10^ This prompted us to explore whether the COD ligand could be displaced by monodentate L-type ligands to afford a coordinatively unsaturated rhodium(i) metal center that has a formal 16-valence electron count. Consequently, the addition of one equivalent of PMe_3_ to [Rh(COD){η^4^-Ge_9_(Hyp)_3_}] led to the reaction shown in Scheme 1. The two hypersilyl environments initially present in the ^1^H NMR spectrum of [Rh(COD){η^4^-Ge_9_(Hyp)_3_}] disappeared giving rise to a single resonance (0.50 ppm). In the ^31^P{^1^H} NMR spectrum a doublet resonance was observed at −13.5 ppm, (^1^*J*_P–Rh_ = 210.3 Hz). This new species, [Rh(PMe_3_){η^3^-Ge_9_(Hyp)_3_}] (1a), and free COD are the only two products observable by ^1^H NMR spectroscopy. The single hypersilyl resonance observed at room temperature points to a fluxional process that makes these groups equivalent on the NMR timescale, as also noted for [Rh(dppe){η^5^-Ge_9_(Hyp)_3_}].^10^

The related clusters [Rh(PPh_3_){η^3^-Ge_9_(Hyp)_3_}] (1b), [Rh(IMe_4_){η^3^-Ge_9_(Hyp)_3_}] (1c), and [Rh{η^3^-Ge_9_(Hyp)_3_}(μ-H)_2_W(Cp)_2_] (1d) were synthesized in a similar manner, from the reaction of [Rh(COD){η^4^-Ge_9_(Hyp)_3_}] with one equivalent of the L-type ligands PPh_3_, IMe_4_ (IMe_4_ = 1,3,4,5-tetramethylimidazol-2-ylidene) or [W(Cp)_2_H_2_],^12–15^ respectively. Clusters 1b–1d exhibit similar NMR spectra to 1a (see ESI† for full details), and are thus also fluxional at room temperature. In the case of 1d, the ^1^H NMR spectrum exhibits a doublet resonance (integrating to two protons) at −16.15 ppm (^1^*J*_Rh–H_ = 26.2 Hz) that exhibits satellites due to ^183^W coupling (^1^*J*_W–H_ is 76.0 Hz), signaling Rh–H–W bridging hydrides. These data are comparable to those found for two known [Rh](μ-H)_2_W(Cp)_2_ complexes reported in the literature, only one of which has been structurally authenticated, *i.e.* [Rh(PPh_3_)_2_(μ-H)_2_W(Cp)_2_][PF_6_].^16–18^

**Fig. 1:**
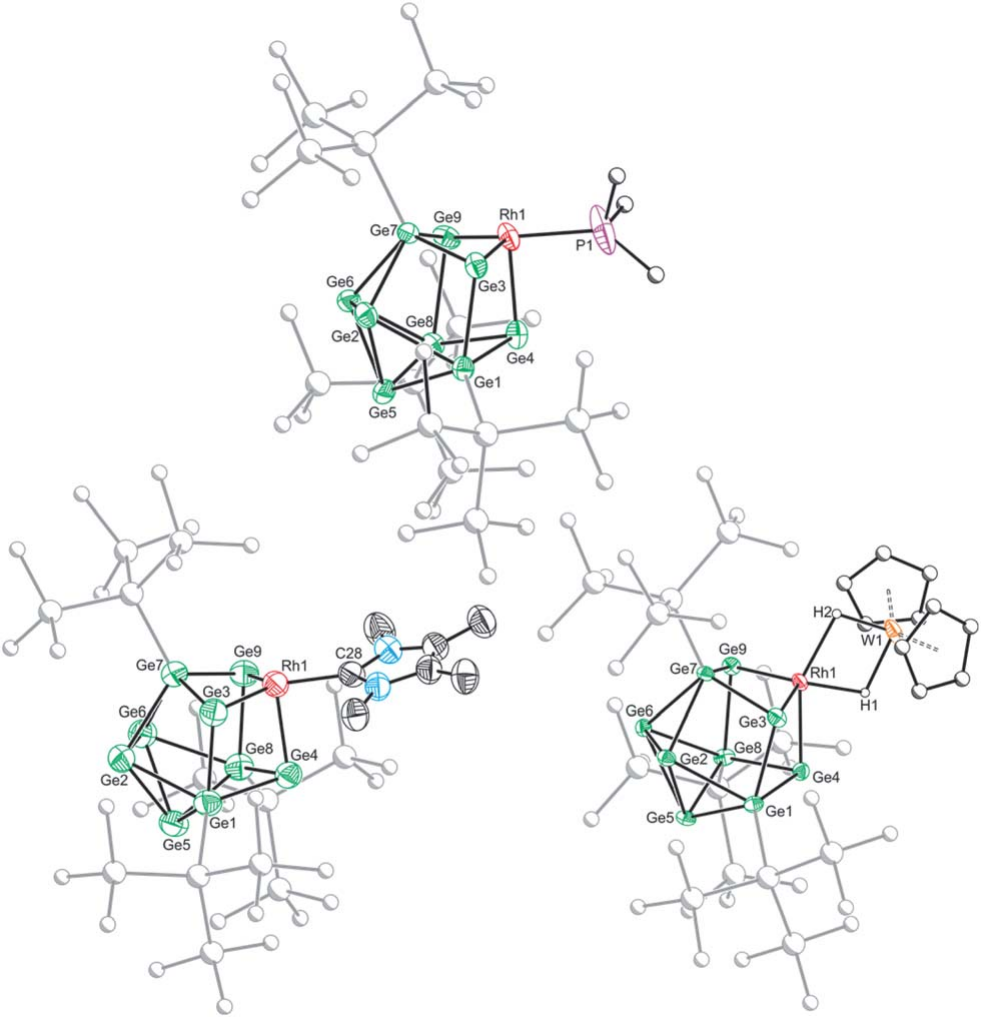
Molecular structures of 1a (top), 1c (bottom left) and 1d (bottom right). Anisotropic displacement ellipsoids are set at 50% probability. Hydrogen atoms have been omitted for clarity. Only the major disordered component is shown.

Crystallization of 1a, 1c and 1d from saturated *n*-hexane or *n*-pentane solutions allowed for structural characterization of the new clusters by single-crystal X-ray diffraction. All three structures exhibit coordinatively unsaturated rhodium(i) centers, bound to the [Ge_9_(Hyp)_3_]^−^ cluster in an η^3^ mode (Fig. 1), in which the ligand (*e.g.* PMe_3_) sits *trans* to a silylated vertex. This gives the cluster overall, non-crystallographic, *C*_s_ symmetry. The *τ*_4_ values for 1a, 1c and 1d are 0.79, 0.83 and 0.85, respectively,‡‡Note that for 1d the *τ*_4_ value is calculated by taking into consideration the Rh–W bond as one of the four bonds. in line with the value for an ideal *C*_3v_ coordination geometry (0.85).^19^ Despite there being an apparent vacant coordination site, no structural or spectroscopic evidence was observed for an interaction between the most proximal hypersilyl substituent with the metal center (*e.g.* the closest Rh⋯C interatomic distances are greater than 3.65 Å).^20^ The structures of 1a and 1d each feature a single crystallographically unique cluster in the asymmetric unit which exhibits positional disorder. This disorder is best accounted for by two different orientations of the {RhL} fragment that are related by rotation with respect to the (static) [Ge_9_(Hyp)_3_]^−^ cluster. By contrast, the structure of 1c reveals two crystallographically unique clusters in the asymmetric unit with near identical bond metrics. The rhodium centers in all the complexes bind to the cluster through three short Rh–Ge bonds (1a: 2.394(1)–2.433(1) Å; 1c: 2.397(1)–2.418(1) Å; 1d: 2.409(2)–2.429(1) Å), and a slightly longer Rh⋯Ge contact with the nearest silylated germanium atom (1a: 2.517(7) Å; 1c: 2.667(1) Å; 1d: 2.644(2) Å). The Rh–L distances for 1a and 1c are similar to those reported in related rhodium−phosphine,^21^ and rhodium–carbene complexes.^22^ In the case of 1d, the Rh⋯W distance of 2.852(1) Å is notably longer than that in [Rh(PPh_3_)_2_(μ-H)_2_W(Cp)_2_][PF_6_] (2.721(3) Å),^16^ which we hypothesize is a consequence of the steric demands of the hypersilyl substituents of the [Ge_9_(Hyp_3_)]^−^ cage. From an electron-counting perspective, 1a–1d can be viewed as *hypercloso*-like, and therefore similar to [Rh(CO)_3_(B_9_H_9_)]^+^.^23^ The effect of displacing a four-electron donor ligand (COD) by a two electron donor (*e.g.* PMe_3_) is to reduce the overall cluster electron count, and this is consequently accompanied by a structural distortion. The reverse effect has previously been seen when *hypercloso*-[Ru(PEt_3_)_2_(C_2_B_7_H_9_)] converts to *closo*-[Ru(PEt_3_)_3_(C_2_B_7_H_9_)] on addition of PEt_3_.^24,25^ Significant rhodium d-orbital participation in cluster bonding can be observed for 1b on inspection of frontier orbitals (see ESI†).

**Fig. 2:**
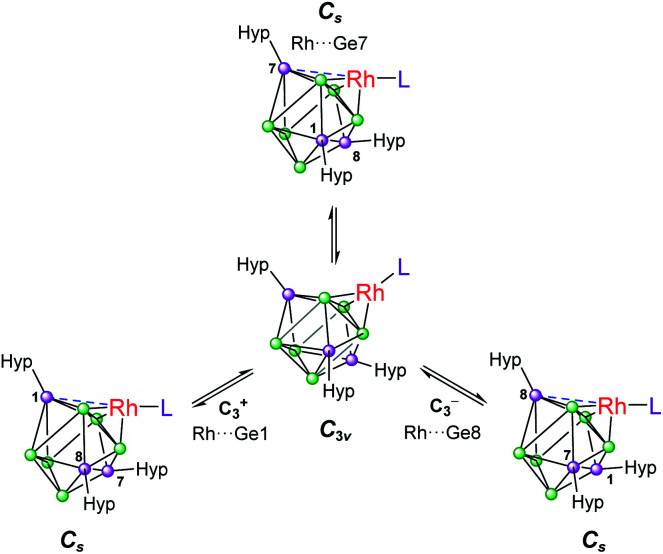
Fluxional behavior of 1b. Numbering of silylated vertices as per Fig. 1.

Based on these crystallographically-determined structures, one would expect two chemically inequivalent hypersilyl substituents to be observed in the solution phase ^1^H NMR spectra of 1a–1d. The fact that only one ^1^H resonance is observed suggests that, in solution, a fluxional process is operating that makes the hypersilyl groups equivalent. This process was modelled using density functional theory (DFT) calculations for 1b, and shown to proceed *via* a *C*_s_ to *C*_3v_ transition (Fig. 2) whereby the rhodium center adopts a tetrahedral coordination geometry and coordinates to a triangular face of the [Ge_9_(Hyp)_3_]^−^ cage. This process involves cleavage of the Rh1⋯Ge7 contact (as per the numbering scheme in Fig. 1) and the contraction of the distance between Ge3 and Ge9 by *ca.* 0.7 Å. Consistent with the observed fluxionality, that is not frozen out at −80 °C, the optimized geometries for the *C*_s_ and *C*_3v_ symmetric clusters were found to be within 2.4 kcal mol^−1^ of one another, with a computed transition state barrier of 4.4 kcal mol^−1^. The positional disorder observed in the single-crystal X-ray diffraction structures of 1a and 1d is consistent with the superposition of two of these *C*_s_ symmetric isomers in the lattice.

Trigonal pyramidal rhodium(i) compounds are rare, and, in some cases, have been shown to be weakly electrophilic.^26–28^ For example, Grützmacher has shown that [Rh(trop_2_SiMe)(C_2_H_4_)] (trop_2_SiMe = bis(5*H*-dibenzo[*a*,*d*]cyclohepten-5-yl)methyl-silane) will reversibly bind THF to afford a trigonal bipyramidal compound. However, this reactivity is finely balanced, as other nucleophiles, such as PPh_3_, will instead displace the coordinated alkene in the equatorial site rather than bind in the axial position.^26^ Reaction of 1a–1d with alkenes indicate that these clusters are not particularly strong electrophiles, as no adduct is observed to be formed to the detection limit of ^1^H NMR spectroscopy. Indeed, inspection of the computed frontier orbitals reveals an energetically accessible HOMO (with a significant rhodium d orbital contribution) suggesting that these complexes are more nucleophilic in character. Thus their reactivity is predicted to mimic that of more traditional organometallic nucleophiles such as Cp*Rh(PMe_3_)_2_.^29^

**Scheme 2:**
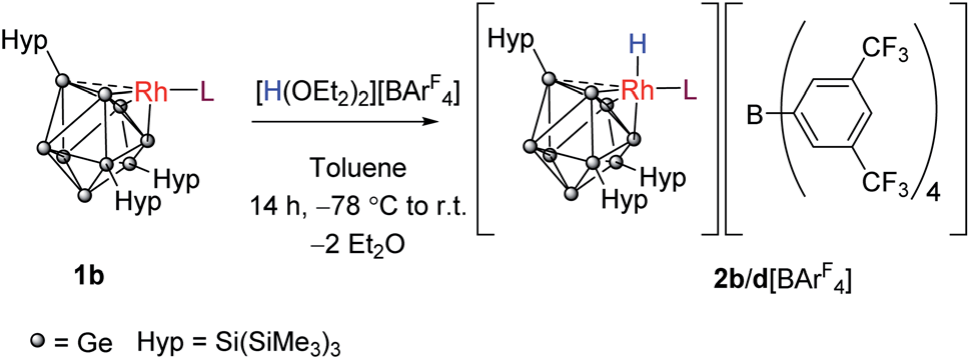
Synthesis of 2b[BAr^F^_4_] and 2d[BAr^F^_4_].

We probed this chemically by reaction of 1b and 1d with Brookhart's acid [H(OEt_2_)_2_][BAr^F^_4_] (Ar^F^ = 3,5-(CF_3_)_2_C_6_H_3_).^30^ Reaction of 1b with 1 equivalent of this acid converts the rhodium(i) center to its conjugate rhodium(iii) hydride, [RhH(PPh_3_){η^3^-Ge_9_(Hyp)_3_}][BAr^F^_4_] (2b[BAr^F^_4_]; Scheme 2). The ^1^H NMR spectrum now shows two new broad singlet resonances at 0.07 and 0.33 ppm (in a 1 : 2 ratio) corresponding to its hypersilyl substituents on a now static Zintl cage,§§Protonation blocks fluxionality *via* a *C*_3v_ symmetric cluster with a tetrahedrally coordinated rhodium(i) center as previously observed for 1a–1d. as well as a new doublet of doublets, integrating to one proton, at −1.48 ppm (^1^*J*_H–Rh_ = 12.6 Hz, ^2^*J*_H–P_ = 3.4 Hz) assigned to Rh–H. ^1^H{^31^P} NMR spectroscopy measurements allowed for the unequivocal assignment of the coupling constants (see ESI†). Similarly, 1d can also be protonated to afford the rhodium(iii) trihydride, 2d[BAr^F^_4_]. On inspection of the ^1^H NMR spectrum of 2d[BAr^F^_4_], the hypersilyl groups present as two inequivalent peaks, and there is a new doublet resonance (−0.03 ppm; ^1^*J*_Rh–H_ = 12.2 Hz) indicating a terminal hydride. The Rh–H–W bridging hydride resonance also shifts from −16.15 ppm (^1^*J*_Rh–H_ = 26.2 Hz, ^1^*J*_W–H_ = 76.0 Hz) to −15.41 ppm (^1^*J*_Rh–H_ = 27.8 Hz, ^1^*J*_W–H_ = 83.3 Hz).

**Fig. 3:**
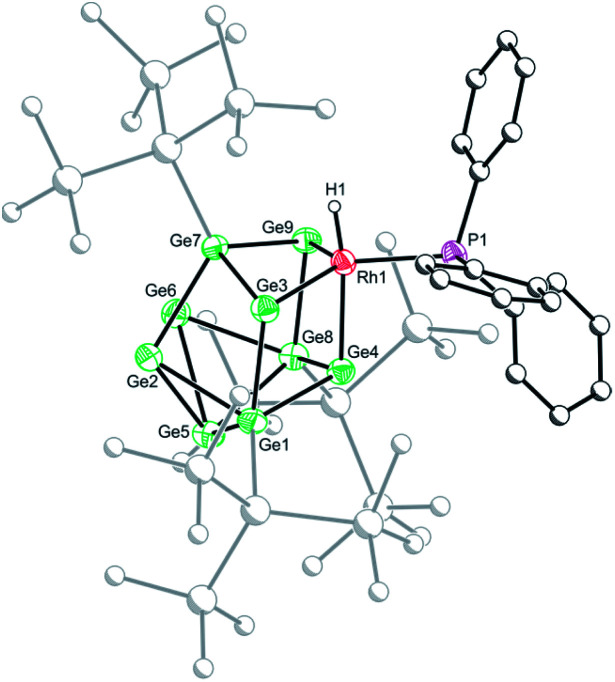
Molecular structure of the cationic moiety in 2b[BAr^F^_4_]·1.5hex. Anisotropic displacement ellipsoids are set at 50% probability. C–H hydrogen atoms have been omitted for clarity.

Crystals of 2b[BAr^F^_4_]·1.5hex (Fig. 3) and 2d[BAr^F^_4_]·Et_2_O suitable for single crystal X-ray diffraction were grown from concentrated *n*-hexane and diethyl ether solutions, respectively. The cationic cluster 2d exhibits positional disorder (two different cluster orientations related by rotation in a 2 : 8 ratio), consequently only the bond metric data for 2b[BAr^F^_4_]·1.5hex will be discussed in detail. The crystal structure reveals a single crystallographically unique cationic cluster in the unit cell, [RhH(PPh_3_){η^3^-Ge_9_(Hyp)_3_}]^+^, accompanied by a [BAr^F^_4_]^−^ counterion and solvent of crystallization. The cluster core of 2b is comparable to those of 1a and 1c, with the notable exception that the rhodium metal center now adopts a trigonal bipyramidal geometry with a (located) hydride in an axial position, fully consistent with protonation of the HOMO in 1b. On protonation, the formal oxidation state of the rhodium center changes from +1 to +3. This is consistent with the moderate contraction (0.03 Å) of the Rh–Ge distances, 2.365(1) to 2.397(1) Å that is observed, relative to those observed for 1a and 1c (*cf.* 2.397(1) to 2.418(1) Å for 1c). As with 1a and 1c, a close Rh⋯Ge contact to the nearest silylated germanium center is seen (2.677(1) Å).

Having probed the electronic structure of 1b and 1d through protonation studies, we turned our attention to see if they might be viable catalysts. Given the presence of an apparent vacant coordination site, we chose to explore the reactivity of these compounds towards dihydrogen activation, a ubiquitous process in organometallic chemistry that often occurs at Rh(i) metal centers.^11^

Placing a C_6_D_6_ solution of 1b under an atmosphere of H_2_ resulted in no change to the ^1^H NMR spectrum. Similarly, no changes were observed to the NMR spectra of 1b if placed under an atmosphere of D_2_. While this observation seemed at odds with the presence of an apparent vacant coordination site on the rhodium(i) center on the cluster, motifs that are well known to oxidatively add H_2_, we show later H_2_ addition is rapid, reversible, and endergonic. We thus turned our attention the dihydride bridged compound 1d to *indirectly* probe the possibility of reversible H_2_ addition to 1b through reaction with D_2_.^31,32^

**Scheme 3:**
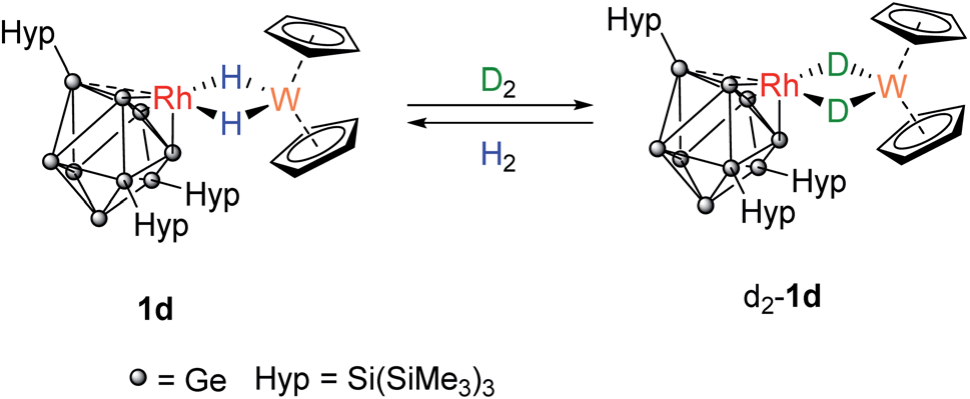
Reversible H_2_/D_2_ exchange at 1d proceeds *via* the Rh(μ-H)(μ-D)W form (not shown).

A C_6_D_6_ solution of 1d was placed under an atmosphere of D_2_ (Scheme 3). Almost immediately, the ^1^H signal for the hydrides (−16.15 ppm) started to decrease in intensity and signals for dissolved H_2_ and HD (4.43 ppm, 1 : 1 : 1 triplet, ^1^*J*_H–D_ = 42.8 Hz) were observed. After approximately 3 hours, two overlapping hydride resonances were clearly visible that are assigned to 1d and its isotopologue [Rh{Ge_9_(Hyp)_3_}(μ-H)(μ-D)W(Cp)_2_}]. Complete loss of the bridging ^1^H signals was observed after 16 hours as [Rh{Ge_9_(Hyp)_3_}(μ-D)_2_W(Cp)_2_], d_2_-1d, now predominates. The presence of the bridging Rh–D–W isotopologue was confirmed by ^2^H NMR spectroscopy, by a resonance observed at −16.06 ppm (^1^*J*_W–D_ = 12.0 Hz; ^1^*J*_Rh–D_ = 4.1 Hz). Replacing the D_2_ atmosphere with H_2_ resulted in complete regeneration of 1d and the observation of dissolved HD, over the course of 24 hours. For context, this reactivity does not occur when [W(Cp)_2_H_2_] is placed under D_2_, although [Rh(PPh_3_)_2_(μ-H)_2_W(Cp)_2_)][PF_6_] does undergo H/D exchange in D_2_O/d_6_-acetone.^16^

The reversible nature of H/D exchange at 1d prompted us to re-evaluate the reaction of 1b with H_2_. We reasoned that perhaps H–H bond activation does indeed take place when the cluster is placed under an H_2_ atmosphere, but that this process is endergonic, rapid and reversible. In order to probe this, 1b was placed under a mixture of H_2_ and D_2_. The immediate formation of HD_(dissolved)_ was observed on time of mixing, indicating fast H_2_ and D_2_ activation on the NMR timescale, with 1b acting a catalyst for H/D exchange. This reaction was unaffected by addition of mercury to the reaction mixture, suggesting a homogeneous process.^33^ To further investigate the H/D exchange process, *para*hydrogen (*p*-H_2_) induced polarization experiments were conducted by reacting 1b and 1d with *p*-H_2_. Due to the hyperpolarization effects arising from a bias in spin relaxation pathways, such experiments can be used to observe short-lived and low concentration intermediates in reactions involving *p*-H_2_.^34–38^ While these experiments did not allow us to directly observe any rhodium−hydride intermediates, two important observations were made allowing us to infer that rapid and reversible H–H bond activation takes place on reaction of 1b or 1d with H_2_. The first of these is that when solutions of 1b or 1d are exposed to NMR silent *p*-H_2_ (3 bar) in an airtight NMR tube, instant formation of NMR detectable *ortho*-dihydrogen (*o*-H_2_) is observed. The second important observation is that when a dilute solution of 1b (0.0005 mmol ml^−1^) was cooled to 263 K in the NMR spectrometer, removed from the magnet and shaken under *p*-H_2_ (3 bar), on returning to the NMR spectrometer a signal with Partially Negative Lineshape (PNL) was observed at 4.56 ppm. This enhancement has a lifetime of around 5 s at this temperature, after which, only *o*-H_2_ was observed. The observation of a PNL effect at 263 K, whist no hydride species are seen, suggests a very rapid and reversible transfer of *p*-H_2_ onto the cluster (most likely the rhodium center) in which the two spins become distinct. This would be consistent with the endergonic formation of either a dihydrogen, Rh(H_2_), or a dihydride, Rh(H)_2_, motif.^39,40^ Both are common intermediates in hydrogenation reactions mediated by organometallic complexes.^11^ At room temperature *p*-H_2_ destruction to form *o*-H_2_ proceeds so rapidly, by reversible formation of a dihydride complex, that the PNL effect is quenched.

What remains undetermined at this stage is the mechanism of H/D exchange by 1b. Oxidative addition of H_2_/D_2_ at the rhodium(i) center is supported by the studies using *p*-H_2_. As this would result in an 18-valence electron rhodium complex, association of a second molecule of H_2_/D_2_ would necessitate a change in the hapticity of the [Ge_9_(Hyp)_3_]^−^ cage (for example from η^3^ to η^1^),^41^*or* phosphine dissociation. The resulting dihydride/σ-dihydrogen compound could undergo H/D exchange *via* a σ-CAM type mechanism.^42,43^ DFT calculations, however, show that cluster isomerization from η^3^ to η^1^ is unfavorable (42.4 kcal mol^−1^), as is phosphine dissociation (32.8 kcal mol^−1^). An alternative is a proton-catalyzed mechanism, as previously invoked by Brookhart and co-workers for the oxidative-addition of H_2_ by the d^8^ iridium(i) complex [Ir(PONOP)(CH_3_)] (PONOP = 2,6-bis(di-*tert*-butylphosphinito) pyridine).^44^ Here H/D exchange would occur by initial reversible protonation of 1b by adventitious water to form trace amounts of formally 16-electron 2b[OH], which would then undergo H/D exchange. To explore such reactivity, 2b[BAr^F^_4_] was exposed to a H_2_/D_2_ mixture. While these studies were hampered by the low solubility of 2b[BAr^F^_4_] and its propensity to precipitate out of solution, they showed that C_6_D_6_ solutions of 2b[BAr^F^_4_] generate HD when exposed to a mixture of H_2_ and D_2_. Moreover, such H/D scrambling occurs on a similar timescale to that observed when using 1b (*i.e.* approx. 15 minutes). Thus, it is possible that adventitious moisture in the solvent or gas mixture could give rise to trace amounts of 2b[BAr^F^_4_] on dissolving 1b and that this species is, in fact, the active catalyst. Arguing against this hypothesis is that addition of proton sponge to 1b H_2_/D_2_ mixture did not suppresses H/D exchange, as might be anticipated for a proton-transfer mechanism. However, we cannot discount the formation of trace [OH]^−^ under these conditions that may act to deprotonate intermediate dihydrogen or hydride complex to form 2b.^45^ While the precise details of the mechanism remain to be resolved, clear is that rapid H/D exchange does occur, a first for a Zintl cluster.

**Scheme 4:**
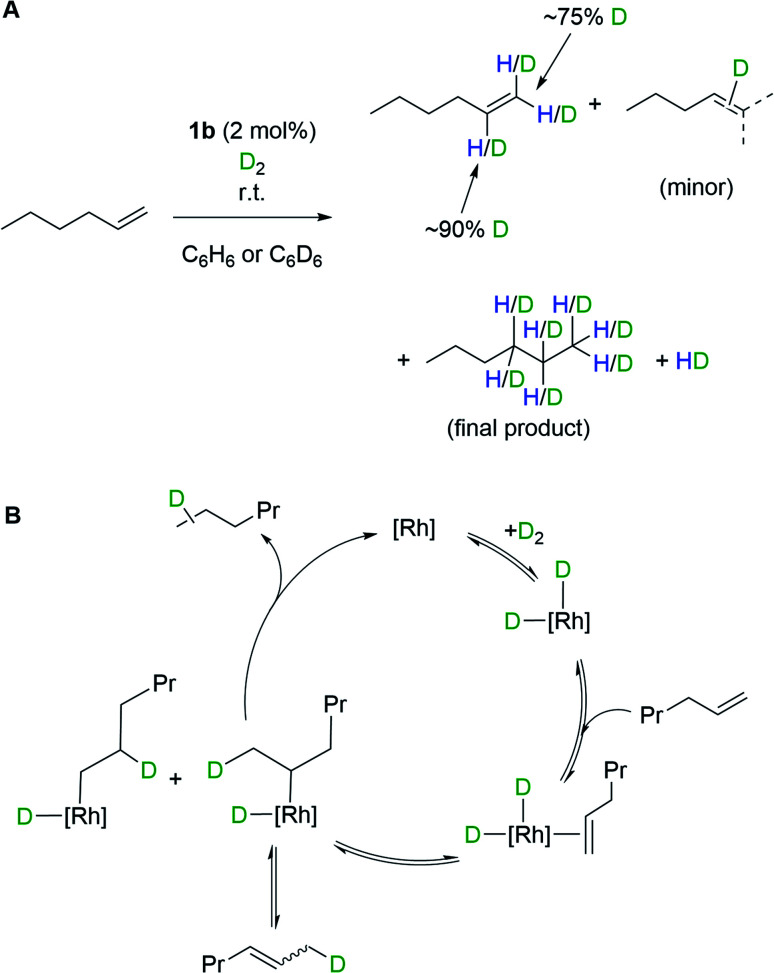
(A) H/D exchange into 1-hexene, as catalyzed by complex 1b. (B) Proposed mechanism.

The oxidative addition of H_2_ to 1b, and the ability to subsequently bind an additional ligand, is further demonstrated by isotope scrambling experiments when 1b is used to catalyze the deuteration of 1-hexene. Addition of 1-hexene to 1b (2 mol%) under a D_2_ atmosphere (1 bar, 16 hours) resulted in the formation of a mixture of the deuterated alkenes d_*n*_-1-hexene (major) and d_*n*_-2-hexene (minor), as well as d_*n*_-hexane, as measured by ^1^H and ^2^H NMR spectroscopy. The d_*n*_-1-hexene has deuterium incorporated into both geminal positions (∼75% D total) as well as the vicinal position (∼90% D) of the alkene. HD_(dissolved)_ is also observed as pictured in Scheme 4A. Recharging with D_2_ results in only d_*n*_-hexane being observed after a further 16 hours, in which deuterium has been incorporated into the 1-, 2- and 3-positions. These observations suggest the formation of di-deuteride intermediate (consistent with both H_2_/D_2_ exchange and *p*-H_2_ experiments), followed by reversible coordination of 1-hexene, and reversible insertion into either of the alkene positions of 1-hexene, followed by a rate-determining reductive elimination of hexane, Scheme 4B. The observation of 2-hexene and d-incorporation into positions 1, 2 and 3 of the final product, hexane, indicates a slower isomerization process also occurs, likely *via* non-degenerative β-elimination from a 2° alkyl-hydride intermediate. As for H_2_/D_2_ exchange we cannot discount that catalysis occurs by a proton-catalyzed mechanism, *via* an (undetected) analogue of mono-hydride 2b[BAr^F^_4_].

## Conclusions

To conclude, we have shown that the cluster [Rh(COD){Ge_9_(Hyp)_3_}] can be modified resulting in coordinatively unsaturated rhodium(i) containing Zintl clusters. Protonation results in a Rh-hydride, while rapid, catalytic H/D exchange occurs between H_2_ and D_2_ in the presence of such clusters. The organometallic chemistry of rhodium-phosphine-hydrides is well established – particularly with supporting cyclopentadienyl ligands – and has been critical in the development of our collective understanding of important concepts in the field, such as structure/property relationships, reactivity patterns and catalysis.^46^ However, the “inorganometallic” chemistry of analogous species with Zintl ions as supporting ligands is essentially unexplored.^47,48^ This contribution shows that molecular TMMGAs offer similar rich structural and bond activation chemistry that suggests further study into their uses in catalysis is warranted.

## Experimental

### Materials and general procedures

All reactions and product manipulations were carried out under an inert atmosphere of argon or dinitrogen using standard Schlenk-line or glovebox techniques (MBraun UNIlab glovebox maintained at < 0.1 ppm H_2_O and < 0.1 ppm O_2_). *n*-pentane (pent; Sigma-Aldrich, HPLC grade, ≥99%), *n*-hexane (hex; Sigma-Aldrich, HPLC grade, ≥97%), benzene (Rathburn, HPLC grade, 99.8%), and toluene (Sigma-Aldrich, HPLC grade, 99.8%), were purified using an MBraun SPS-800 solvent system. C_6_D_6_ (Sigma-Aldrich, 99.5%) and C_7_D_8_ (Sigma-Aldrich, 99.5%) were distilled over sodium metal/benzophenone. All dry solvents were stored under argon in gas-tight ampoules over activated 3 Å molecular sieves. K_4_Ge_9_ was synthesized by heating a mixture of the elements (potassium, 99.95%, Alfa Aesar; germanium powder −100 mesh, 99.999%, Alfa Aesar) at 900 °C for 2 days in sealed niobium containers jacketed in evacuated fused-silica ampules according to previously reported synthetic procedures. [Rh(COD)Cl]_2_,^49^ K{Ge_9_[Si(SiMe_3_)_3_]_3_},^50^ [Rh(COD){Ge_9_(Hyp_3_)_3_}],^10^ IMe_4_ (IMe_4_ = 1,3,4,5-tetramethylimidazol-2-ylidene),^51^ Na[B(3,5-C_6_H_3_(CF_3_)_2_)_4_],^52^ and [H(OEt_2_)_2_][B(3,5-C_6_H_3_(CF_3_)_2_)_4_]^30^ were prepared according to literature procedures. Triphenylphosphine (PPh_3_; Sigma Aldrich, 99%), trimethylphosphine (PMe_3_; Sigma Aldrich, 97%) chlorotris(trimethylsilyl)silane (HypCl; TCI, 97%) rhodium(iii) chloride hydrate (Precious Metals Online), 1,5-cyclooctadiene (Sigma-Aldrich, >99%), and proton sponge (Sigma-Aldrich, 99%) were used as received without further purification. Parahydrogen was sourced as previously described.^37^

NMR samples were prepared inside an inert atmosphere glovebox in NMR tubes fitted with a gas-tight valve. ^1^H NMR spectra were recorded at either 499.9 MHz or 400.1 MHz on a Bruker AVIII 500 or a Bruker AVIII 400 NMR spectrometer, respectively. ^13^C{^1^H} NMR spectra were recorded at either 151 MHz, 125.8 MHz or 100.6 MHz on a Bruker NEO 600 with broadband helium cryoprobe, a Bruker AVII 500 fitted with a cryoprobe or a Bruker AVIII 400 NMR spectrometer, respectively. ^31^P{^1^H} NMR spectra were recorded at 202.4 MHz or 162.0 MHz on a Bruker AVIII 500 or a Bruker AVIII 400 NMR spectrometer, respectively. ^1^H and ^13^C{^1^H} NMR spectra are reported relative to TMS and referenced to the most downfield residual solvent resonance. ^31^P{^1^H} NMR spectra are externally referenced to an 85% solution of H_3_PO_4_ in H_2_O (*δ* = 0 ppm).

Elemental analyses were carried out by Elemental Microanalyses Ltd (Devon, UK). Samples (approx. 10 mg), submitted in sealed Pyrex ampoules.

### Synthetic procedures

#### Synthesis of [Rh(PMe_3_){η^3^-Ge_9_(Hyp)_3_}] (1a)

[Rh(COD){Ge_9_(Hyp)_3_}] (100 mg, 0.062 mmol) was dissolved in toluene (20 ml) and cooled to −77 °C. PMe_3_ in toluene (0.031 M, 2 ml, 0.062 mmol) was added dropwise over the course of 10 minutes. The brown solution was stirred for 90 minutes before warming to room temperature and stirring for 4 hours. The toluene was removed *in vacuo* and the product was extracted in *n*-hexane (10 ml) as a brown solution. The product was dried under a dynamic vacuum and lyophilized from benzene (4 ml). Yield: 91 mg, 93%. CAUTION: fine dry powders of 1a ignite spontaneously in air. Black-brown crystals suitable for X-ray crystallography were obtained by dissolving 50 mg of 1a in 0.5 ml of *n*-hexane and cooling to −40 °C. Elemental analysis calcd for C_30_H_90_Ge_9_PRhSi_12_ (*M* = 1575.62 g mol^−1^): C 22.87, H 5.76. Found: C 23.98, H 5.84. ^1^H NMR (C_6_D_6_, 298 K, 400.2 MHz): *δ* (ppm) 0.50 (s, 81H; CH_3_), 1.13 (d, ^2^*J*_H–P_ = 9.3 Hz, 9H; CH_3_). ^13^C{^1^H} NMR (C_6_D_6_, 298 K, 100.64 MHz): *δ* (ppm) 3.13 (Si(*C*H_3_)_3_), 26.78 (*d*, ^1^*J*_C–P_ = 28.2 Hz; P*C*H_3_). ^29^Si NMR (C_6_D_6_, 298 K, 99.32 MHz): *δ* (ppm) −95.2, −9.7. ^31^P{^1^H} NMR (C_6_D_6_, 298 K, 202.39 MHz): *δ* (ppm) −13.47 (d, ^1^*J*_P–Rh_ = 210.3 Hz).

#### Synthesis of [Rh(PPh_3_){η^3^-Ge_9_(Hyp)_3_}] (1b)

Toluene (20 ml) was added to a mixture of [Rh(COD){Ge_9_(Hyp)_3_}] (250 mg, 0.16 mmol) and PPh_3_ (41 mg, 0.16 mmol) at room temperature. The brown solution was stirred for 3 hours before heating to 70 °C for 4 days. The toluene was removed *in vacuo* and the product was extracted in *n*-pentane (10 ml) as a brown solution. The product was dried under a dynamic vacuum and lyophilized from benzene (3 ml). The resulting fine brown powder was purified by sublimation of any excess (typically ∼5%) PPh_3_ at 85 °C over 12 hours. This was then again extracted with *n*-pentane (5 ml) and lyophilized from benzene (3 ml) to yield 1b as a brown powder (240 mg, 84%). CAUTION: fine dry powders of 1b ignite spontaneously in air. Black-brown crystals suitable for X-ray crystallography were obtained by dissolving 200 mg of the powder in 0.5 ml of *n*-hexane and cooling to −40 °C. Elemental analysis calcd for C_45_H_96_Ge_9_PRhSi_12_ (*M* = 1761.83 g mol^−1^): C 30.68, H 5.49. Found: C 30.71, H 5.77. ^1^H NMR (C_6_D_6_, 298 K, 499.93 MHz): *δ* (ppm) 0.46 (s, 81H; CH_3_), 6.99 (m, 3H; CH), 7.04 (m, 6H; CH), 7.66 (m, 6H; CH). ^13^C{^1^H} NMR (C_6_D_6_, 298 K, 125.72 MHz): *δ* (ppm) 3.04 (Si(*C*H_3_)_3_), 128.57 (CH), 128.65 (CH), 130.19 (CH), 134.75 (CH), 134.86 (CH) 136.10 (d, ^1^*J*_C–P_ = 41.4 Hz; CP). ^29^Si NMR (C_6_D_6_, 298 K, 99.32 MHz): *δ* (ppm) −94.2, −9.6. ^31^P{^1^H} NMR (C_6_D_6_, 298 K, 202.39 MHz): *δ* (ppm) 49.28 (d, ^1^*J*_P–Rh_ = 214.0 Hz).

#### Synthesis of [Rh(IMe_4_){η^3^-Ge_9_(Hyp)_3_}] (1c)

[Rh(COD){Ge_9_(Hyp)_3_}] (100 mg, 0.062 mmol) was dissolved in *n*-pentane (5 ml) and added to a suspension of IMe_4_ (8 mg, 0.062 mmol) in *n*-pentane (5 ml) at room temperature. The brown suspension was stirred for 16 hours before filtering. The solution was concentrated to ∼1 ml and cooled to −40 °C producing black crystals of compound 1c (60 mg, 58%) which were suitable for single crystal X-ray diffraction. Elemental analysis calcd for C_34_H_93_Ge_9_N_2_RhSi_12_(C_5_H_12_) (*M* = 1623.73 + 72.15 g mol^−1^): C 27.64, H 6.19, N 1.65. Found: C 27.19, H 6.02, N 1.77. ^1^H NMR (C_6_D_6_, 298 K, 499.93 MHz): *δ* (ppm) 0.55 (s, 81H; CH_3_), 1.16 (s, 6H; CH_3_), 3.38 (s, 6H; CH_3_). ^13^C{^1^H} NMR (C_6_D_6_, 298 K, 125.72 MHz): *δ* (ppm) 3.28 (Si(*C*H_3_)_3_), 9.00 (CH_3_), 40.38 (CH_3_), 171.4 (d, ^1^*J*_C–Rh_ = 76.0 Hz; Rh*C*). ^29^Si NMR (C_6_D_6_, 298 K, 99.32 MHz): *δ* (ppm) −97.7, −9.7.

#### Synthesis of [Rh{η^3^-Ge_9_(Hyp)_3_}(μ-H)_2_W(Cp)_2_] (1d)

[Rh(COD){Ge_9_(Hyp)_3_}] (300 mg, 0.19 mmol) was dissolved in *n*-hexane (20 ml) and cooled to −78 °C. (Cp)_2_WH_2_ (60 mg, 0.19 mmol) was dispersed in *n*-hexane (10 ml). The stirring (Cp)_2_WH_2_ mixture was then added dropwise to the solution of [Ge_9_(Hyp)_3_]Rh(COD) over the course of 20 minutes. This was allowed to stir and warm to room temperature for 18 hours. The solution was then reduced in volume to ∼10 ml *in vacuo* and stirred at room temperature under a static vacuum for 4 days, producing a brown suspension. Upon standing for 3 hours, the mixture was filtered, affording 1d as a brown powder. The product was dried *in vacuo* and lyophilized from benzene (5 ml) to yield a fine brown powder of 1d (240 mg, 71%). CAUTION: powders of 1d are highly pyrophoric. Black crystals suitable of X-ray crystallography were obtained by dissolving 30 mg in a 1 : 10 mixture of toluene and *n*-pentane (∼0.5 ml total volume) and cooling to −40 °C. Attempts to heat the reaction to above 45 °C or stir the reaction longer (∼1 week) resulted in the formation of decomposition products. Elemental analysis calcd for C_37_H_93_Ge_9_RhSi_12_W (*M* = 1815.59 g mol^−1^): C 24.48, H 5.16. Found: C 23.62, H 4.24. ^1^H NMR (C_6_D_6_, 298 K, 400.16 MHz): *δ* (ppm) −16.15 (d, ^1^*J*_Rh–H_ = 26.2 Hz, ^1^*J*_W–H_ = 76.0 Hz, 2H; Rh*H*_2_W), 0.59 (s, 81H; CH_3_), 4.26 (s, 10H; CH). ^13^C{^1^H} NMR (C_6_D_6_, 298 K, 125.72 MHz): *δ* (ppm) 3.26 (Si(*C*H_3_)_3_), 77.55 (CH). ^29^Si NMR (C_6_D_6_, 298 K, 99.32 MHz): *δ* (ppm) −95.21, −9.55.

#### Synthesis of [RhH(PPh_3_){η^3^-Ge_9_(Hyp)_3_}][BAr^F^_4_] (2b[BAr^F^_4_])

Compound 1b (100 mg, 0.057 mmol) and [H(OEt_2_)_2_][BAr^F^_4_] (60 mg, 0.059 mmol) were added to an ampoule, which was quickly cooled to −78 °C. The solids were stirred and cold toluene (−78 °C, 10 ml) was added. The reaction was stirred for 30 minutes before warming slowly to room temperature overnight to produce a reddish-brown solution and brown oil. This was filtered to a Schlenk flask and washed with toluene (3 × 10 ml) until the extracts were almost colourless. The red-brown solution was dried *in vacuo* to produce a red oil. *n*-Hexane (30 ml) was then added and the flask and shaken to disperse the oil as a dark red mixture, this was then allowed to stand overnight, producing black-red crystals of 2b[BAr^F^_4_]. N.B. Due to the insolubility of 2b[BAr^F^_4_] in C_6_D_6_, it can also be prepared in Et_2_O *in situ* as evidenced by ^31^P{^1^H} NMR spectroscopy, however, attempts to isolate solids from this reaction were unsuccessful. Elemental analysis calcd for C_53_H_105_Ge_9_P_2_RhSi_12_ (*M* = 2626.06 g mol^−1^): C 35.22, H 4.18. Found: C 31.92, H 3.63. ^1^H NMR (C_6_D_6_, 298 K, 400.17 MHz): *δ* (ppm) −1.48 (dd, ^1^*J*_H–Rh_ = 12.6 Hz, ^2^*J*_H–P_ = 3.4 Hz, 1H; RhH), 0.07 (s br, 27H; CH_3_), 0.33 (s br, 54H; CH_3_), 7.01 (m, 9H; CH), 7.37 (m, 6H; CH), 7.70 (s, 4H; CH), 8.46 (s, 8H; CH). ^13^C{^1^H} NMR (C_6_D_6_, 298 K, 151 MHz): *δ* (ppm) 2.32 (Si(*C*H_3_)_3_), 3.19 (Si(*C*H_3_)_3_), 118.06 (m; CH), 125.30 (q, one signal hidden under C_6_D_6_, ^1^*J*_C–F_ = 272.2 Hz; CF_3_), 129.25 (d, ^3^*J*_C–P_ = 10.4 Hz; CH), 129.95 (qq, ^2^*J*_C–F_ = 30.6 Hz, ^3^*J*_C–B_ = 2.8 Hz; *C*CF_3_), 131.50 (d, ^1^*J*_C–P_ = 45.1 Hz; CP), 131.84 (d, ^4^*J*_C–P_ = 2.2 Hz; CH), 134.43 (d, ^2^*J*_C–P_ = 13.2 Hz; CP), 135.50 (br. s; CH), 162.90 (q, ^1^*J*_C–B_ = 50.58 Hz; CB). ^11^B{^1^H} NMR (C_6_D_6_, 298 K, 128.40 MHz): *δ* (ppm) −5.84 (s, 1B; BAr^F^_4_). ^19^F{^1^H} NMR (C_6_D_6_, 298 K, 376.53 MHz): *δ* (ppm) −61.67 (s, 24F; CF_3_). ^29^Si NMR (C_6_D_6_, 298 K, 79.5 MHz): *δ* (ppm) −83.87, −8.38. ^31^P{^1^H} NMR (C_6_D_6_, 298 K, 162 MHz): *δ* (ppm) 41.39 (d, ^1^*J*_P–Rh_ = 188.9 Hz).

#### Synthesis of [RhH{η^3^-Ge_9_(Hyp)_3_}(μ-H)_2_W(Cp)_2_][BAr^F^_4_] (2d[BAr^F^_4_])

A mixture of 1d (50 mg, 0.027 mmol) and [H(OEt_2_)_2_][BAr^F^_4_] (28 mg, 0.027 mmol) were added to an ampoule, which was quickly cooled to −78 °C. The solids were stirred and cold toluene was added. The reaction was stirred for 30 minutes before warming slowly to room temperature overnight to produce a reddish-brown solution and brown oil. This was filtered to a Schlenk flask and washed with toluene (3 × 10 ml) until the extracts were almost colourless. The red-brown solution was dried *in vacuo* to produce a red-brown oil. This was then dissolved in Et_2_O (3 ml) and slowly evaporated to ∼1 ml before cooling to −40 °C, producing dark red crystals of 2d[BAr^F^_4_] (70 mg, 64%). Elemental analysis calcd for C_69_H_106_BF_24_Ge_9_RhSi_12_W (*M* = 2679.81 g mol^−1^): C 30.93, H 3.99. Found: C 29.58, H 3.81. ^1^H NMR (C_6_D_6_, 298 K, 400.17 MHz): *δ* (ppm) −15.41 (d, ^1^*J*_Rh–H_ = 27.8 Hz, ^1^*J*_W–H_ = 83.3 Hz, 2H; RhH_2_W), −0.03 (d, ^1^*J*_Rh–H_ = 12.2 Hz, 1H; RhH), 0.21 (s, 27H; CH_3_), 0.42 (s, 54H; CH_3_), 4.31 (s, 10H; CH). ^13^C{^1^H} NMR (C_6_D_6_, 298 K, 151 MHz): *δ* (ppm) 2.45 (CH_3_), 2.71 (CH_3_), 79.71 (s; CH), 118.12 (m; CH), 125.20 (q, one signal hidden under C_6_D_6_, ^1^*J*_C–F_ = 272.2 Hz; CF_3_), 129.94 (qq, ^2^*J*_C–F_ = 31.6 Hz, ^3^*J*_C–B_ = 3.0 Hz; *C*CF_3_), 135.44 (br. s; CH), 162.80 (q, ^1^*J*_C–B_ = 50.58 Hz; CB). ^11^B{^1^H} NMR (C_6_D_6_, 298 K, 128.40 MHz): *δ* (ppm) −5.96 (s, 1B; BAr^F^_4_). ^19^F{^1^H} NMR (C_6_D_6_, 298 K, 376.53 MHz): *δ* (ppm) −61.89 (s, 24F; CF_3_). ^29^Si NMR (C_6_D_6_, 298 K, 79.5 MHz): *δ* (ppm) −85.83, −84.26, −8.57, −7.98.

#### Synthesis of [Rh{η^3^-Ge_9_(Hyp)_3_}(μ-D)_2_W(Cp)_2_] (d_2_-1d)

Compound 1d (15 mg, 0.01 mmol) was dissolved in toluene (0.4 ml) in an airtight NMR tube. This was degassed by three freeze–pump–thaw cycles and pressurized with D_2_ (1 bar). The reaction mixture was stirred by tumbling the NMR tube overnight producing compound d_2_-1d, H_2_ and HD. Degassing the sample and replacing the atmosphere with H_2_ (1 bar), regenerates 1d quantitatively by NMR spectroscopy. ^2^H NMR (C_7_H_8_, 298 K, 76.74 MHz): *δ* (ppm) −16.06 (d, ^1^*J*_Rh–D_ = 4.1 Hz; ^1^*J*_W–D_ 12 Hz, 2D; RhD_2_W).

## Data availability

Spectral data, selected crystallographic information and computational data are included in the ESI.†

## Author contributions

O. P. E. T. carried out all of the synthetic and computational work described in the manuscript, O. P. E. T. and J. M. G. conducted the crystallographic studies, O. P. E. T. and S. B. D. conducted the *para*hydrogen experiments, and A. S. W. and J. M. G. wrote the manuscript and managed the project.

## Conflicts of interest

There are no conflicts to declare.

## Supplementary Material

SC-013-D2SC02552C-s001

SC-013-D2SC02552C-s002

SC-013-D2SC02552C-s003
