# Supplementary material for: Zintl cluster supported low coordinate Rh(i) centers for catalytic H/D exchange between H2 and D2
Source: Chem Sci. 2022 Jun 9;13(25):7626–33. doi: 10.1039/d2sc02552c (PMC9242017; doi:10.1039/d2sc02552c)
Supplement: SC-013-D2SC02552C-s002 [file SC-013-D2SC02552C-s002.pdf]

Electronic Supplementary Material (ESI) for Chemical Science.
This journal is © The Royal Society of Chemistry 2022

Electronic Supplementary Information

Zintl cluster supported low coordinate Rh(I) centers for catalytic H/D exchange between H_2 and D_2

Table of Contents

1. NMR Spectra 3

1.1. NMR spectra for [Rh(PMe_3){Ge_9(Hyp)$_3$}] (**1a**). 3

1.2. NMR spectra for [Rh(PPh_3){Ge_9(Hyp)$_3$}] (**1b**). 5

1.3. NMR spectra for [Rh(IMe_4){Ge_9(Hyp)$_3$}] (**1c**). 7

1.4. NMR spectra for [Rh{Ge_9(Hyp)$_3$}(μ-H)$_2$W(Cp)$_2$] (**1d**). 9

1.5. NMR spectra for [RhH(PPh_3){Ge_9(Hyp)$_3$}][BAr$^F{}_4$] (**2b**[BAr$^F{}_4$]). 12

1.6. NMR spectra for [RhH{Ge_9(Hyp)$_3$}(μ-H)$_2$W(Cp)$_2$][BAr$^F{}_4$] (**2d**[BAr$^F{}_4$]). 16

1.7. NMR spectra for [Rh{Ge_9(Hyp)$_3$}(μ-D)$_2$W(Cp)$_2$] (d_2-**1d**). 19

2. X-ray Crystallography 21

3. Catalysis Procedures 25

3.1. H/D Scrambling 25

3.2. H/D Scrambling with Alkenes 27

4. Computational Details 30

4.1. Methodology 30

4.2. Fluxional Behavior 31

4.3. η^3–η^1 Isomerisation 33

4.4. Phosphine Dissociation 35

4.5. Oxidative Addition of H_2 at **1b** 36

4.6. Oxidative Addition of H_2 at **2b** 39

4.7. Computational Appendix 41

5. References..87

1. NMR Spectra

1.1. NMR spectra for [Rh(PMe$_3$){Ge$_9$(Hyp)$_3$}] (**1a**).

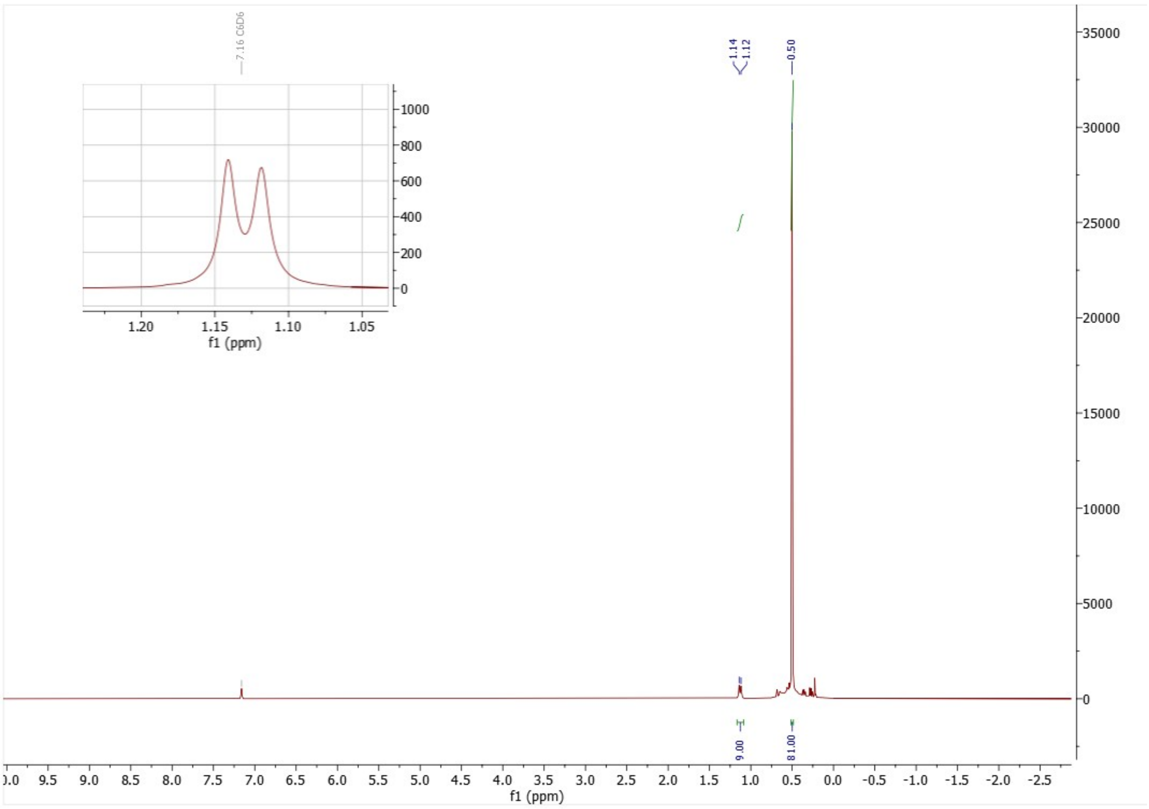

Figure S1. ^{1}H NMR spectrum (C$_6$D$_6$, 298 K, 400 MHz) of [Rh(PMe$_3$){Ge$_9$(Hyp)$_3$}] (**1a**).

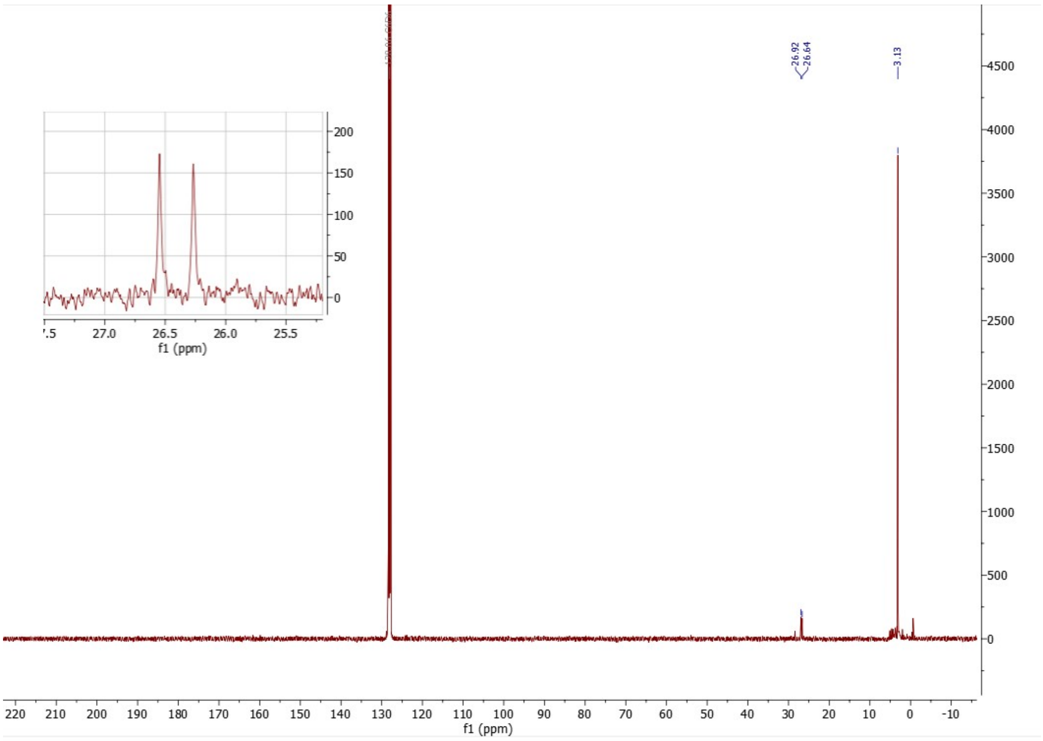

Figure S2. ^{13}C{^{1}H} NMR spectrum (C$_6$D$_6$, 298 K, 100.64 MHz) of [Rh(PMe$_3$){Ge$_9$(Hyp)$_3$}] (**1a**).

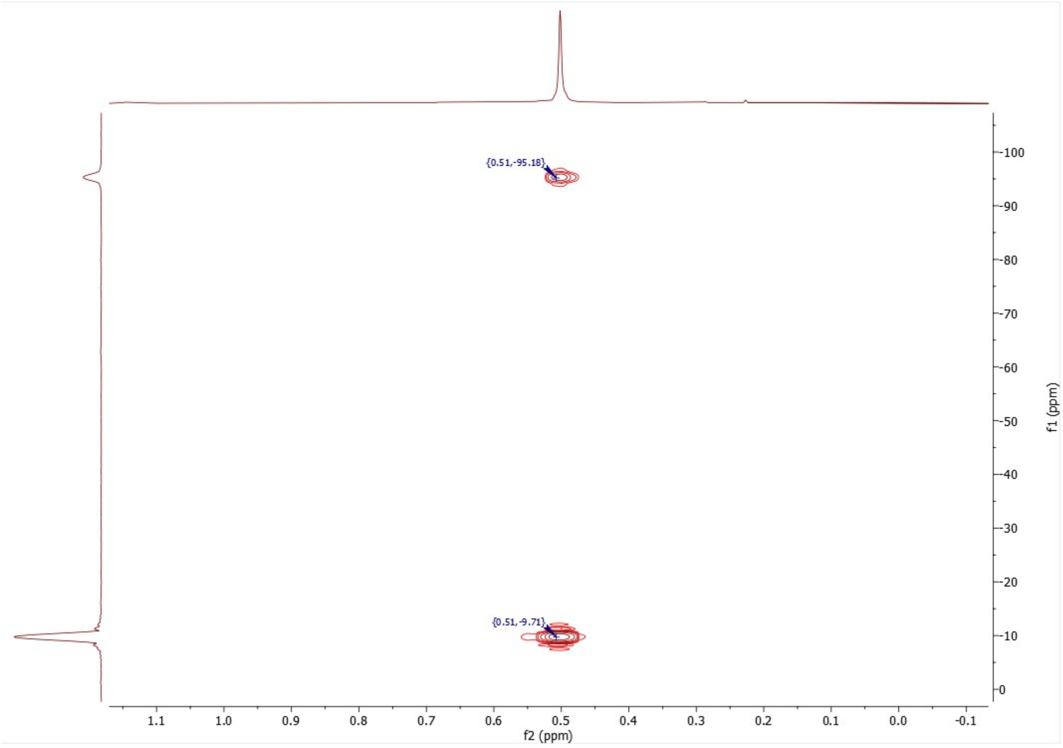

Figure S3. $^{1}H/^{29}Si$ HMBC NMR spectrum (C_6D_6, 298 K, 99.32 MHz) of [Rh(PMe_3){$Ge_9(Hyp)_3$}] (**1a**).

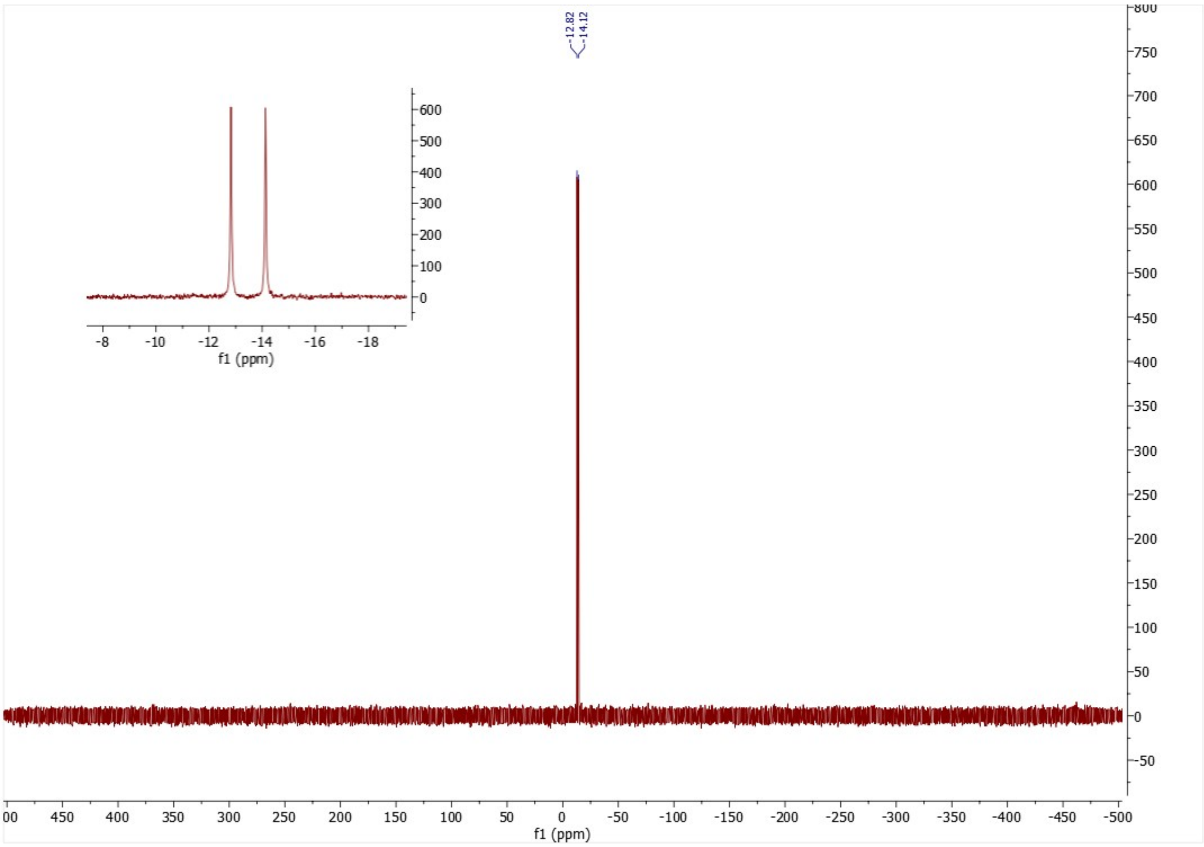

Figure S4. $^{31}P\{^{1}H\}$ NMR spectrum (C_6D_6, 298 K, 202.39 MHz) of [Rh(PMe_3){$Ge_9(Hyp)_3$}] (**1a**).

1.2. NMR spectra for [Rh(PPh$_3$){Ge$_9$(Hyp)$_3$}] (**1b**).

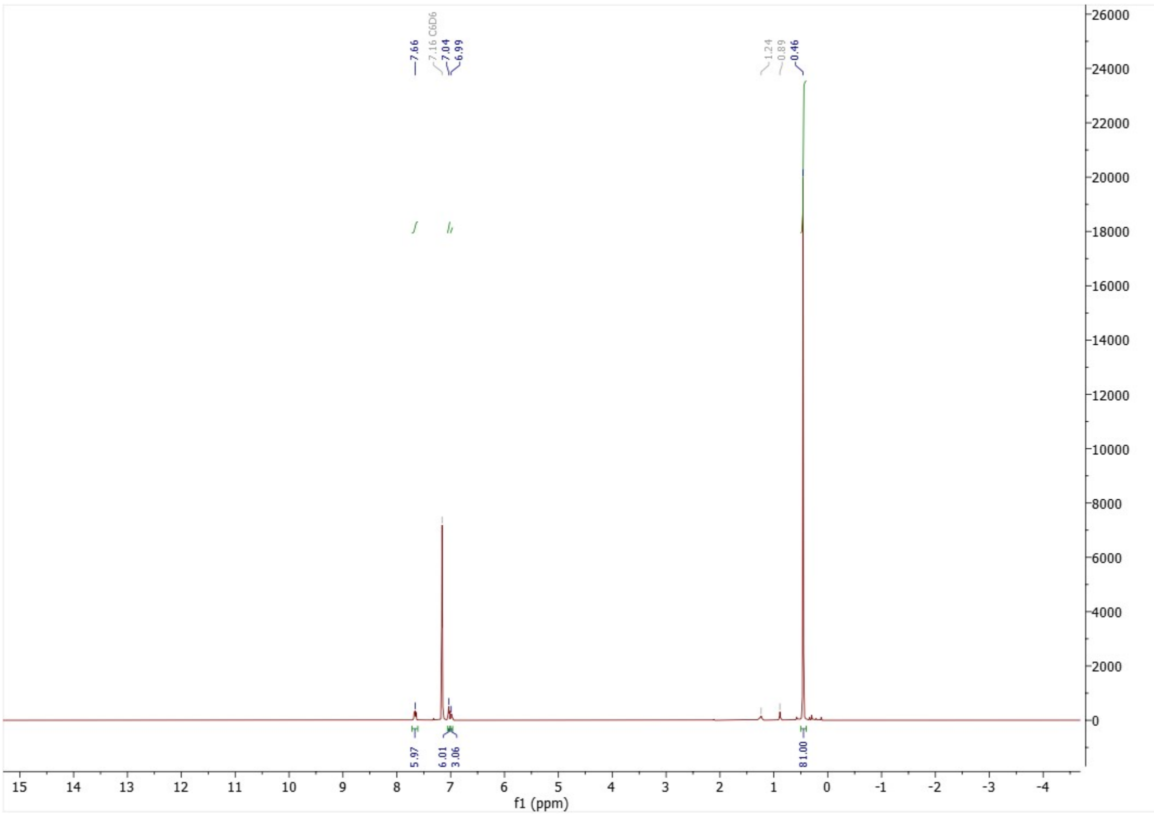

Figure S5. ^{1}H NMR spectrum (C_6D_6, 298 K, 500 MHz) of [Rh(PPh$_3$){Ge$_9$(Hyp)$_3$}] (**1b**).

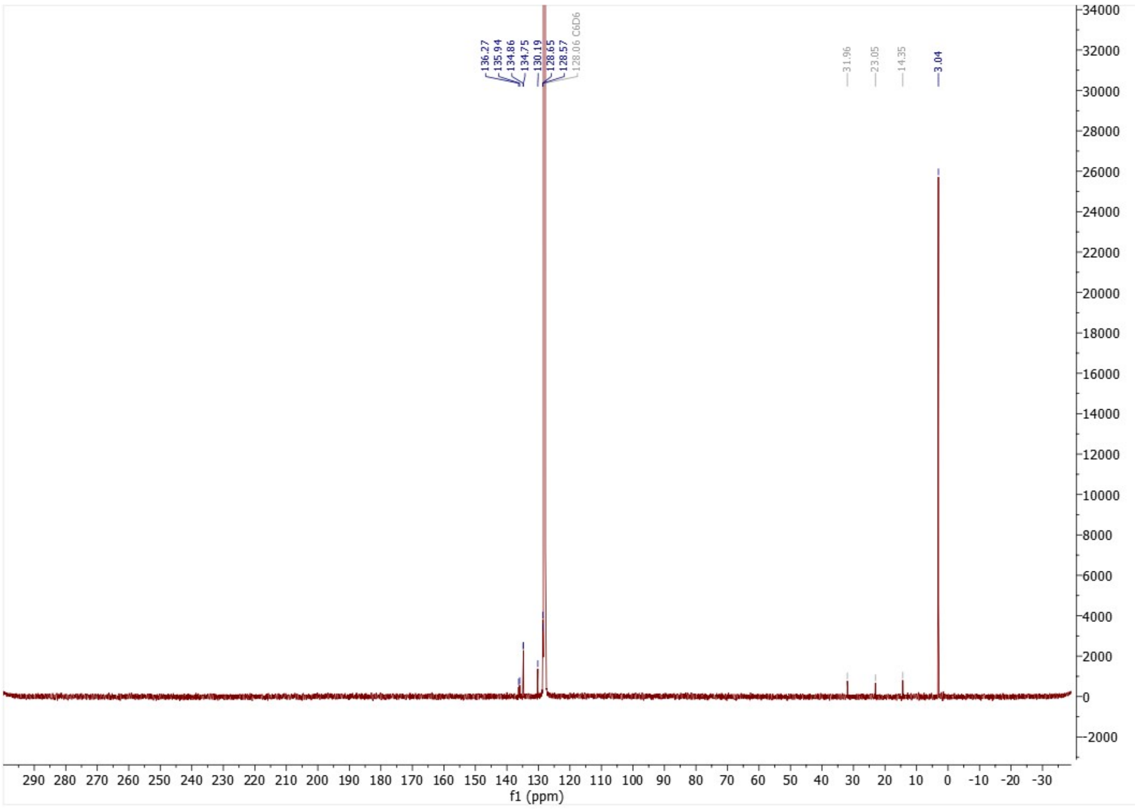

Figure S6. ^{13}C{^{1}H} NMR spectrum (C_6D_6, 298 K, 125.81 MHz) of [Rh(PPh$_3$){Ge$_9$(Hyp)$_3$}] (**1b**).

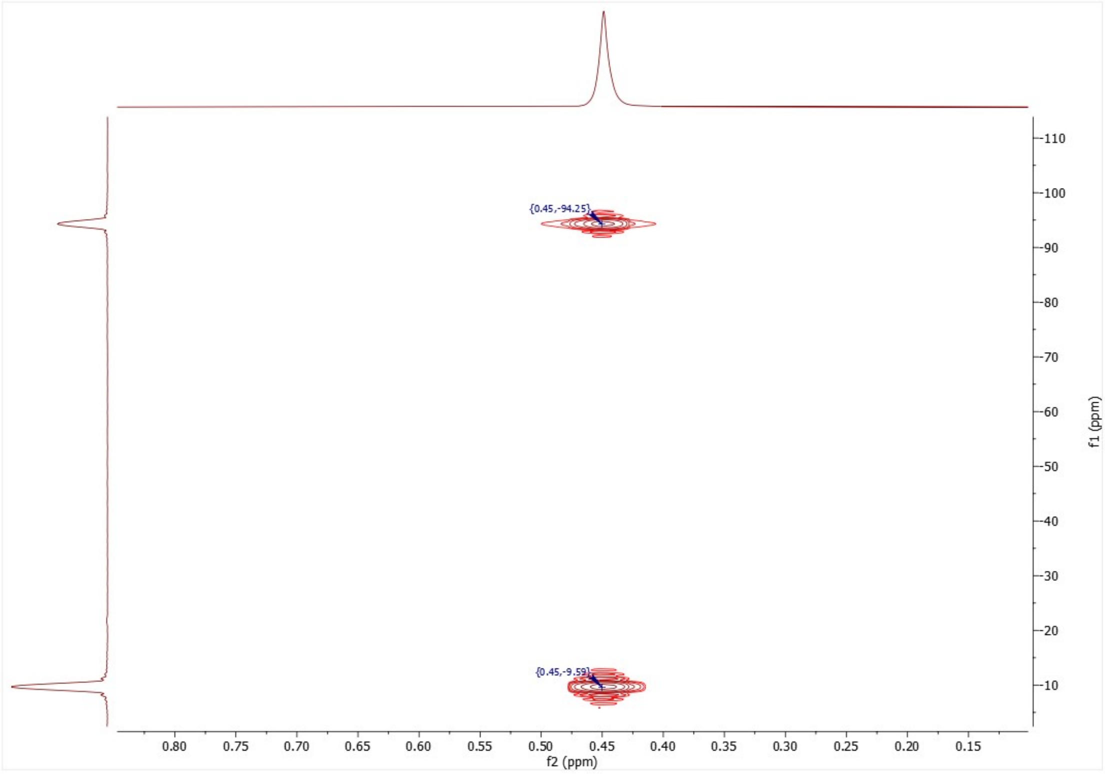

Figure S7. $^{1}H/^{29}Si$ HMBC NMR spectrum (C_6D_6, 298 K, 99.32 MHz) of [Rh(PPh$_3$){Ge$_9$(Hyp)$_3$}] (**1b**).

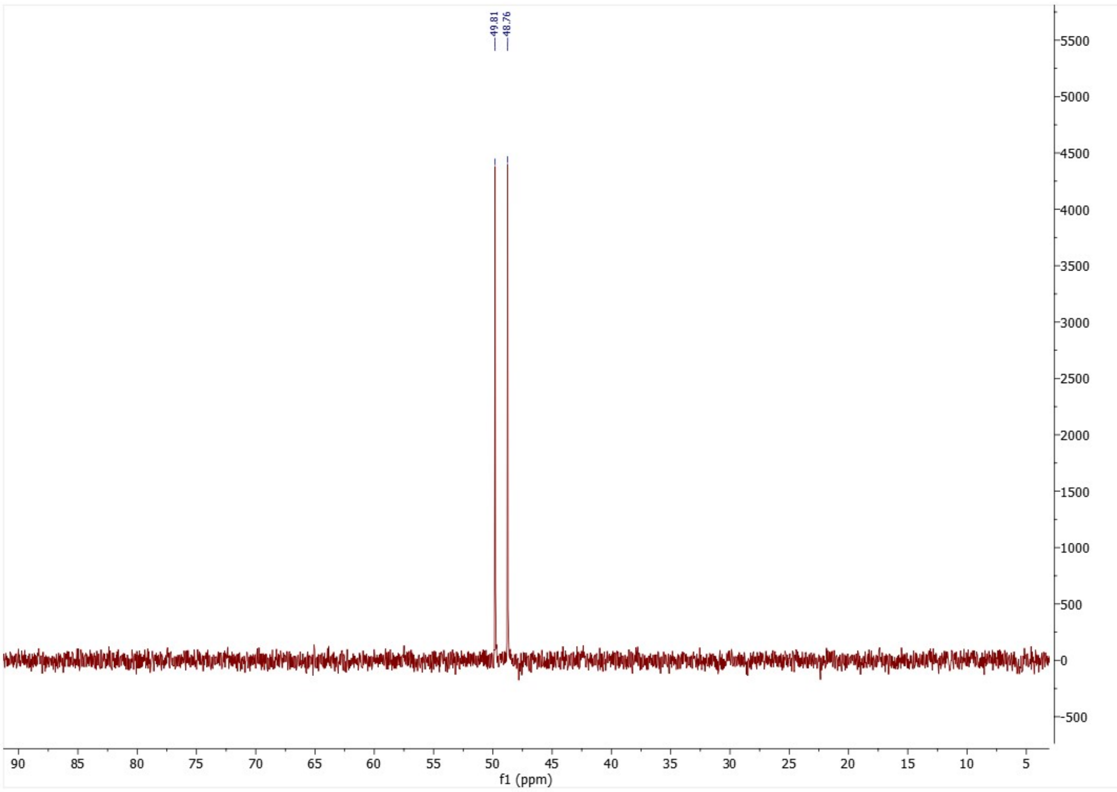

Figure S8. $^{31}P\{^{1}H\}$ NMR spectrum (C_6D_6, 298 K, 202.39 MHz) of [Rh(PPh$_3$){Ge$_9$(Hyp)$_3$}] (**1b**).

1.3. NMR spectra for [Rh(IMe$_4$){Ge$_9$(Hyp)$_3$}] (**1c**).

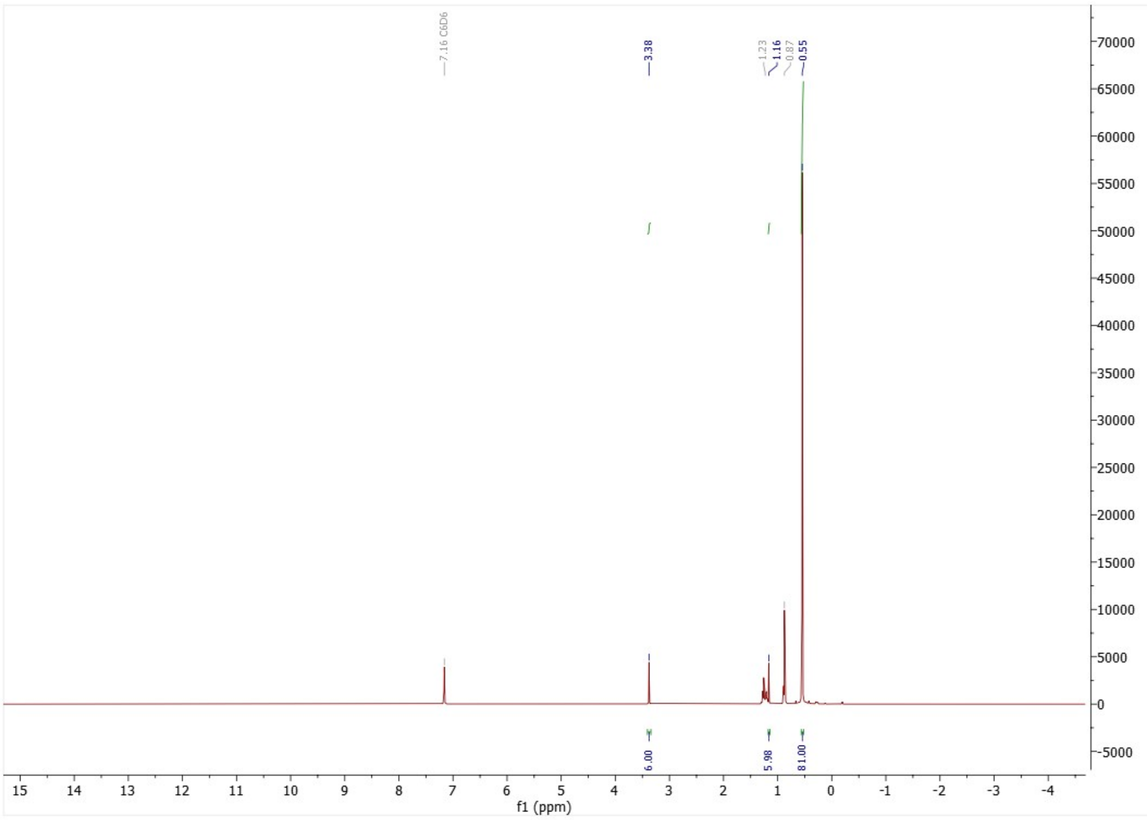

Figure S9. ^{1}H NMR spectrum (C_6D_6, 298 K, 500 MHz) of [Rh(IMe$_4$){Ge$_9$(Hyp)$_3$}] (**1c**).

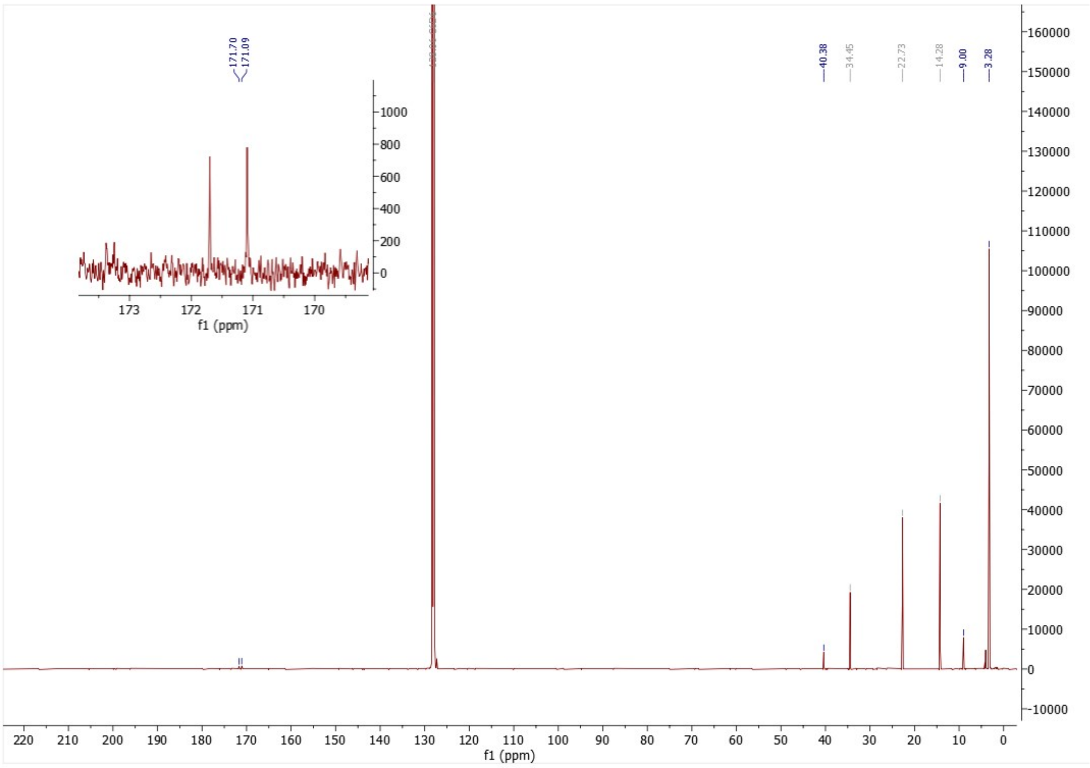

Figure S10. ^{13}C{^{1}H} NMR spectrum (C_6D_6, 298 K, 125.81 MHz) of [Rh(IMe$_4$){Ge$_9$(Hyp)$_3$}] (**1c**).

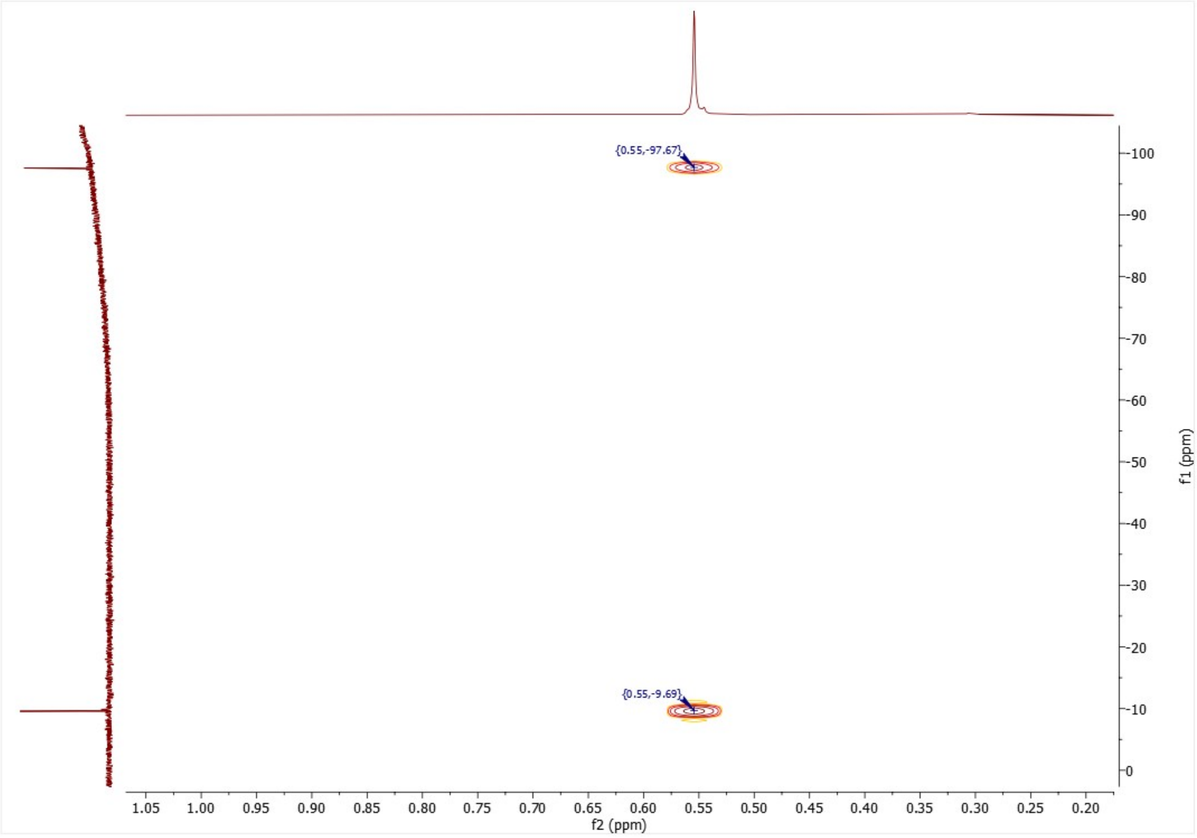

Figure S11. $^{1}H/^{29}Si$ HMBC NMR spectrum (C_6D_6, 298 K, 99.32 MHz) of [Rh(IMe$_4$){Ge$_9$(Hyp)$_3$}] (**1c**).

1.4. NMR spectra for [Rh{Ge_9(Hyp)$_3$}(μ-H)$_2$W(Cp)$_2$] (**1d**).

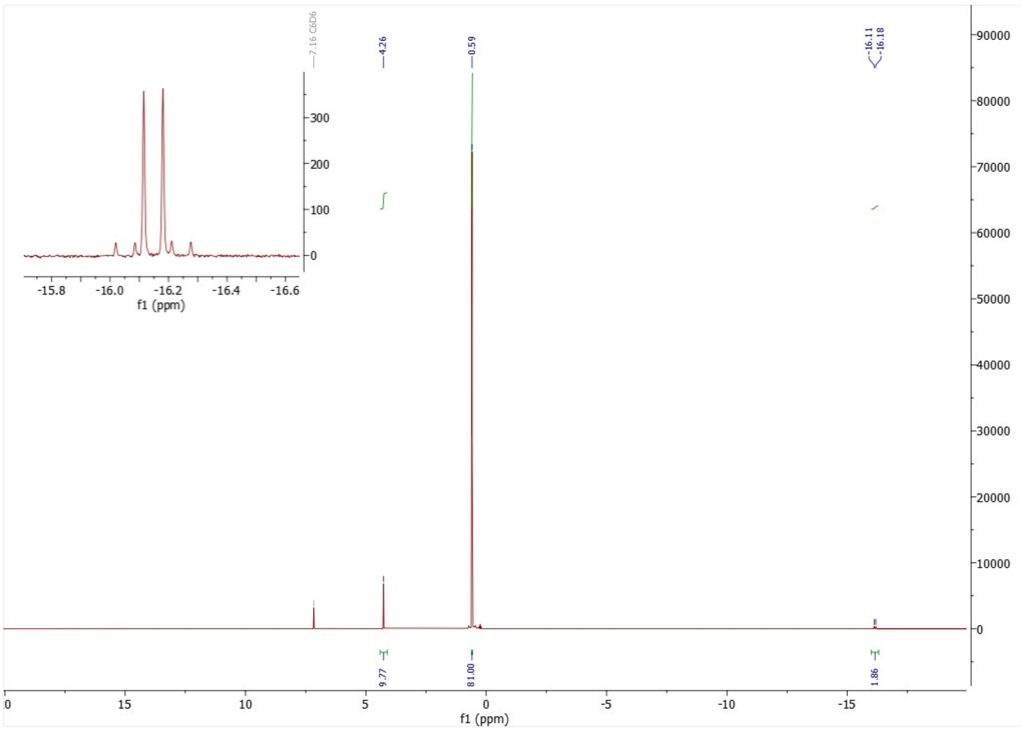

Figure S12. ^{1}H NMR spectrum (C_6D_6, 298 K, 400.16 MHz) of [Rh{Ge_9(Hyp)$_3$}(μ-H)$_2$W(Cp)$_2$] (**1d**).

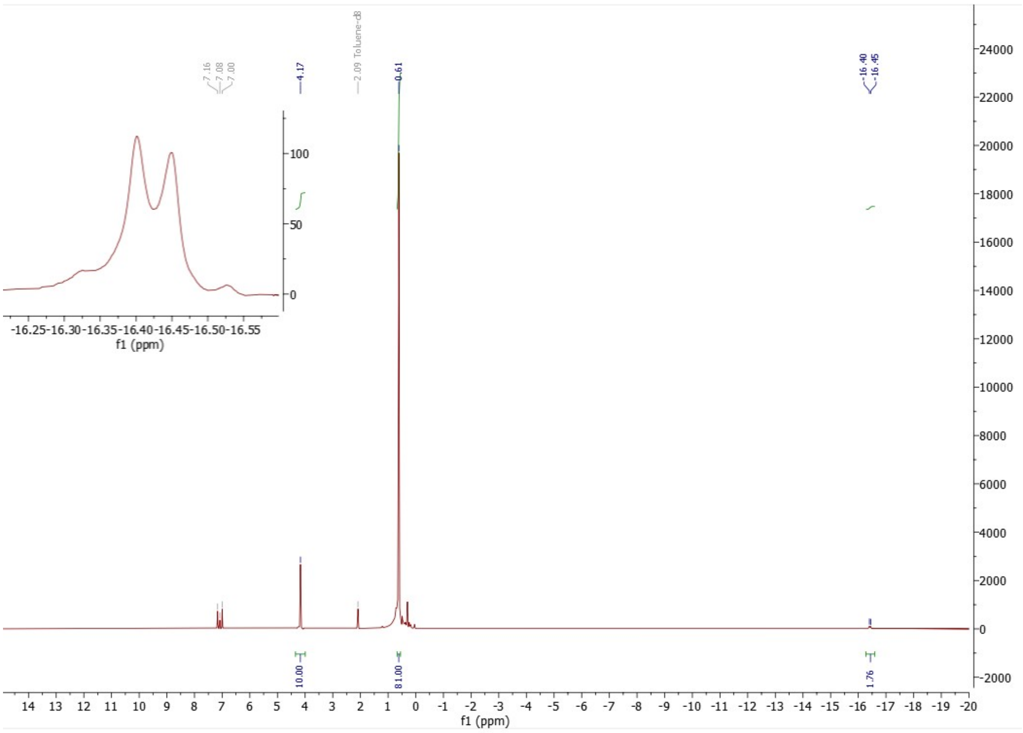

Figure S13. ^{1}H NMR spectrum (C_6D_6, 193 K, 499.93 MHz) of [Rh{Ge_9(Hyp)$_3$}(μ-H)$_2$W(Cp)$_2$] (**1d**).

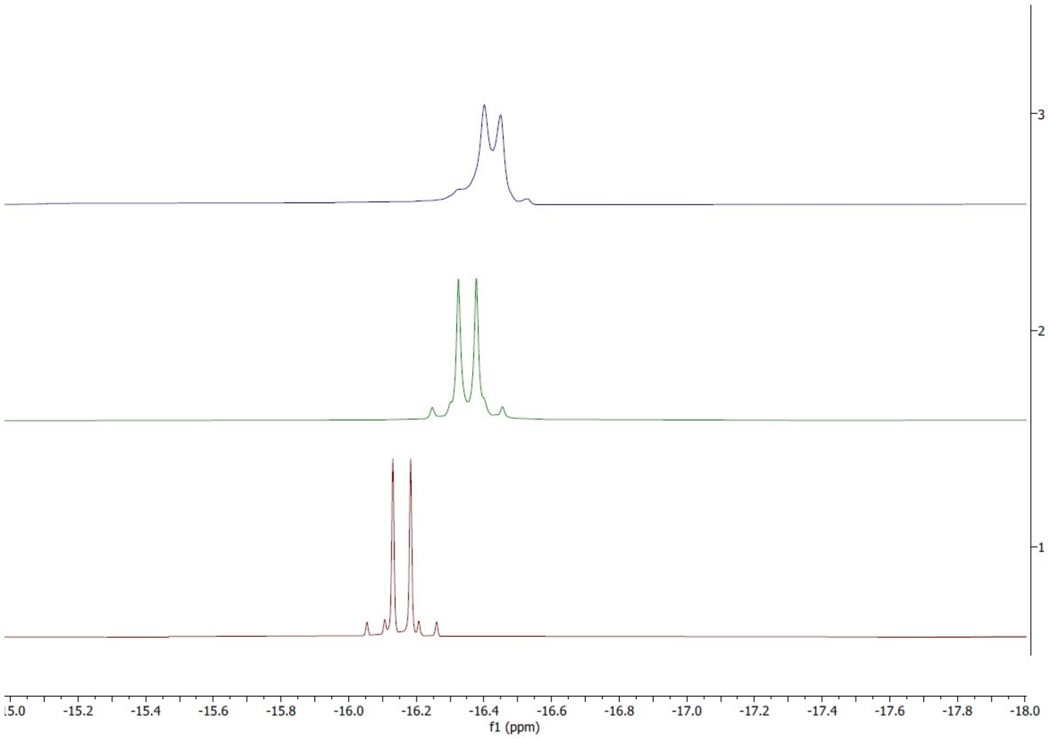

Figure S14. Variable temperature ^{1}H NMR (dihydride region) spectrum (C_7D_8, 499.93 MHz) of $[Rh\{Ge_9(Hyp)_3\}(\mu\text{-}H)_2W(Cp)_2]$ (**1d**). From bottom to top: *T* = 298, 223, and 193 K.

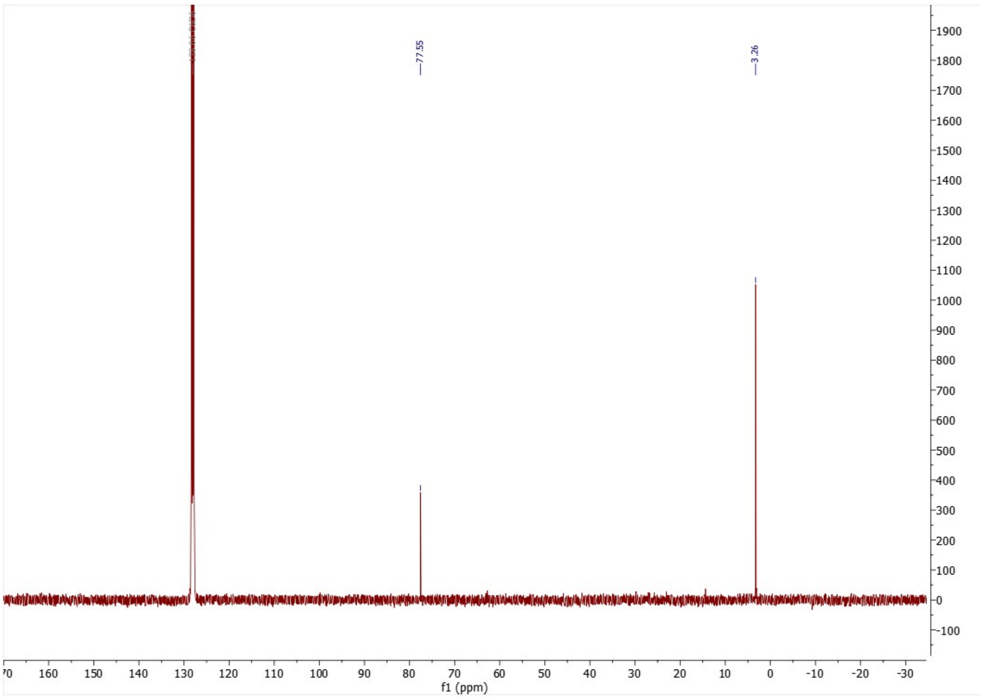

Figure S15. $^{13}C\{^{1}H\}$ NMR spectrum (C_6D_6, 298 K, 100.64 MHz) of $[Rh\{Ge_9(Hyp)_3\}(\mu\text{-}H)_2W(Cp)_2]$ (**1d**).

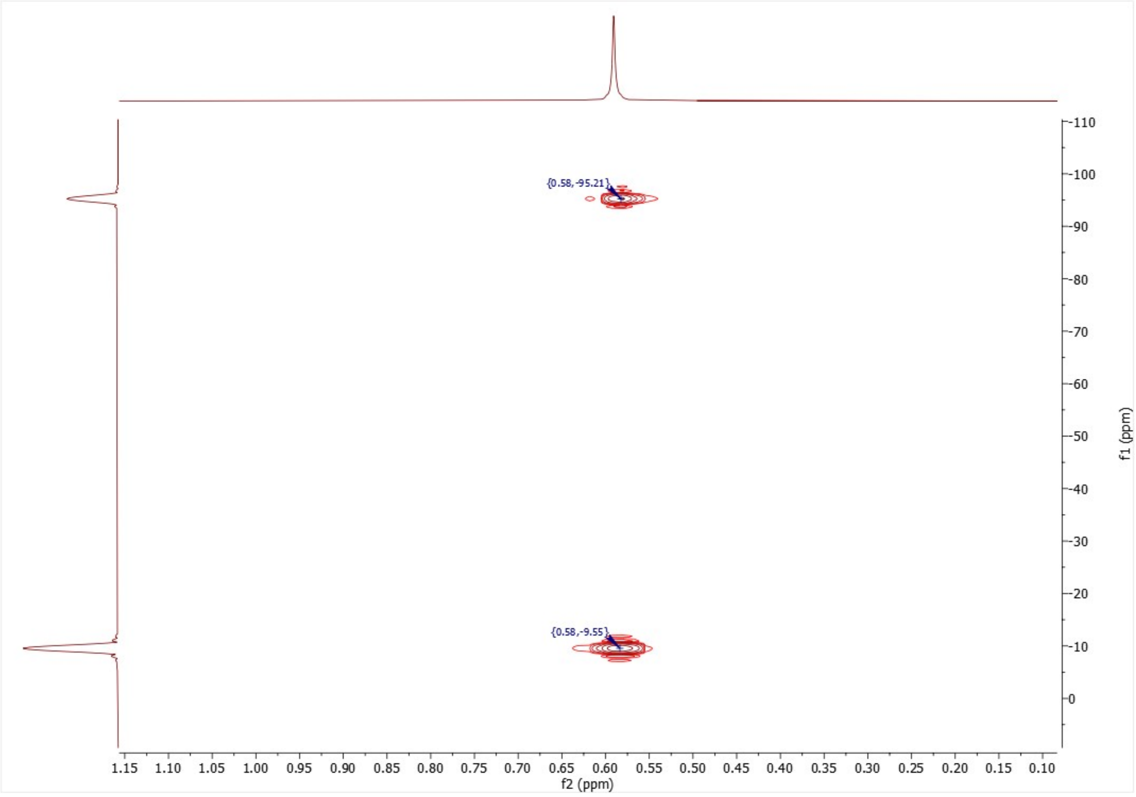

Figure S16. $^{29}Si/^{1}H$ HMBC NMR spectrum (C_6D_6, 298 K, 79.51 MHz) of [Rh{$Ge_9(Hyp)_3$}(μ-$H)_2W(Cp)_2$] (**1d**).

1.5. NMR spectra for [RhH(PPh_3){Ge_9(Hyp)$_3$}][$BAr^F{}_4$] (**2b**[$BAr^F{}_4$]).

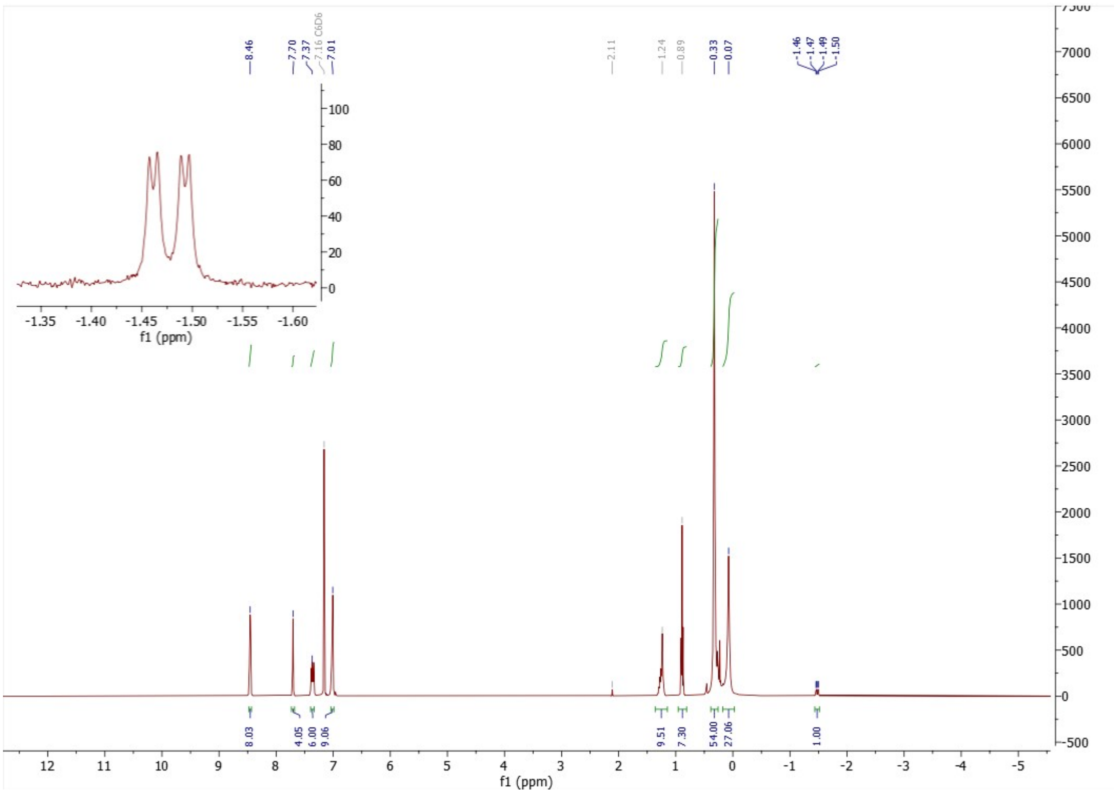

Figure S17. ^{1}H NMR spectrum (C_6D_6, 298 K, 400 MHz) of [RhH(PPh_3){Ge_9(Hyp)$_3$}][$BAr^F{}_4$] (**2b**[$BAr^F{}_4$]).

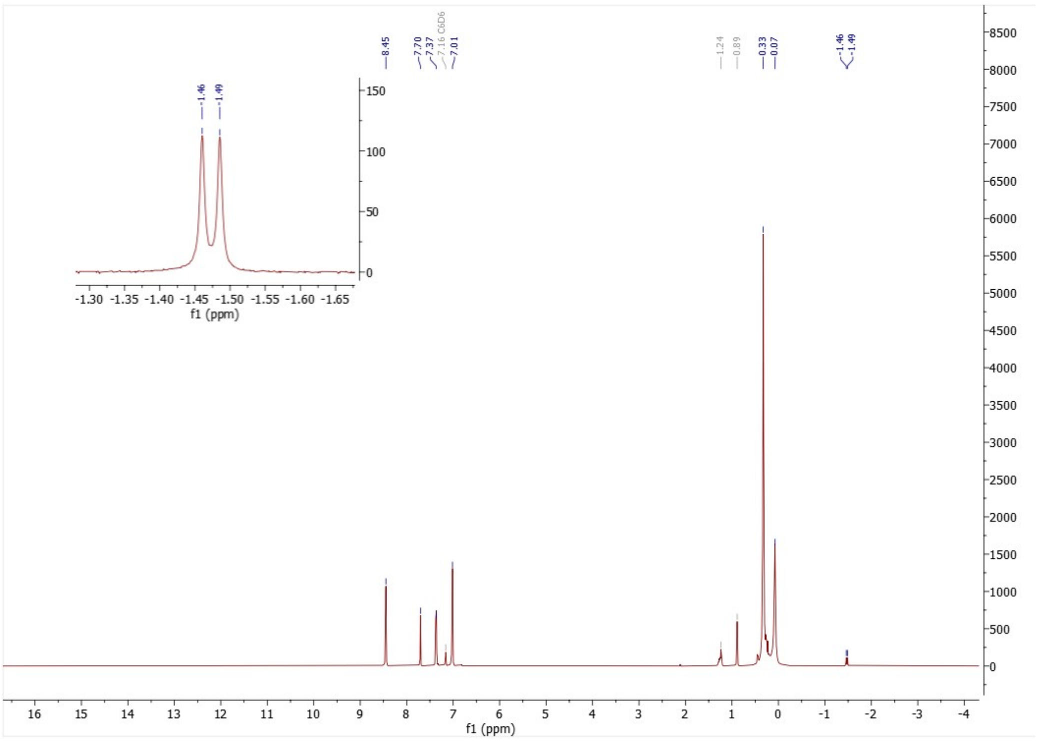

Figure S18. ^{1}H{^{31}P} NMR spectrum (C_6D_6, 298 K, 499.94 MHz) of [RhH(PPh_3){Ge_9(Hyp)$_3$}][$BAr^F{}_4$] (**2b**[$BAr^F{}_4$]).

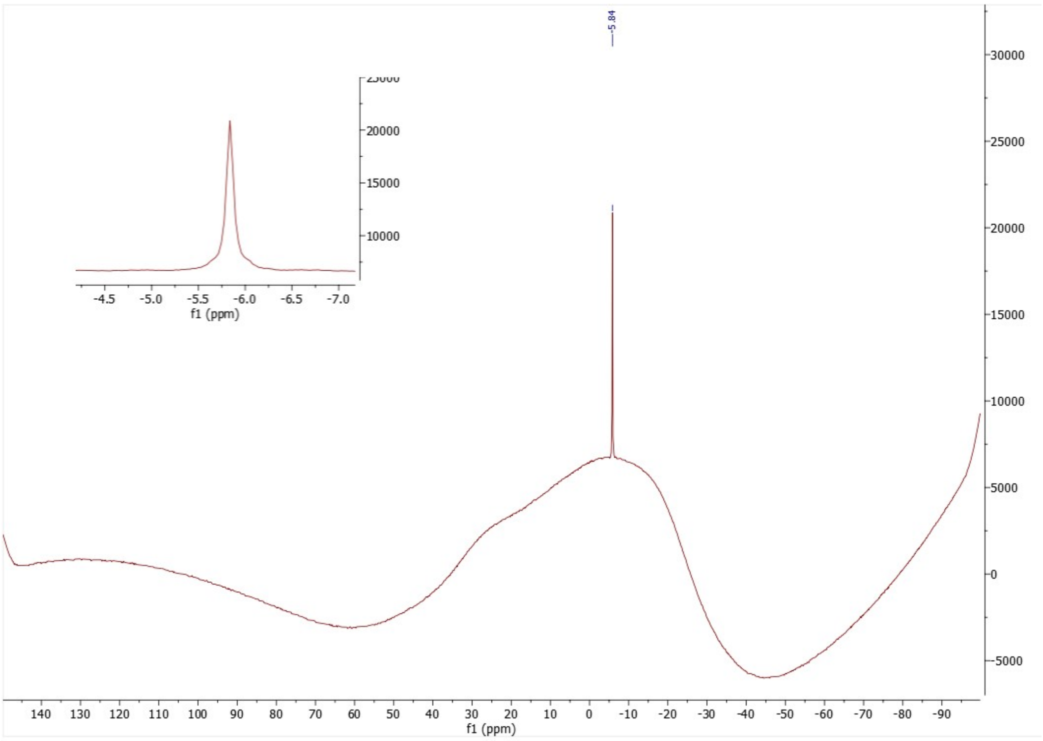

Figure S19. $^{11}B\{^{1}H\}$ NMR spectrum (C_6D_6, 298 K, 128.40 MHz) of $[RhH(PPh_3)\{Ge_9(Hyp)_3\}][BAr^F{}_4]$ (**2b**$[BAr^F{}_4]$).

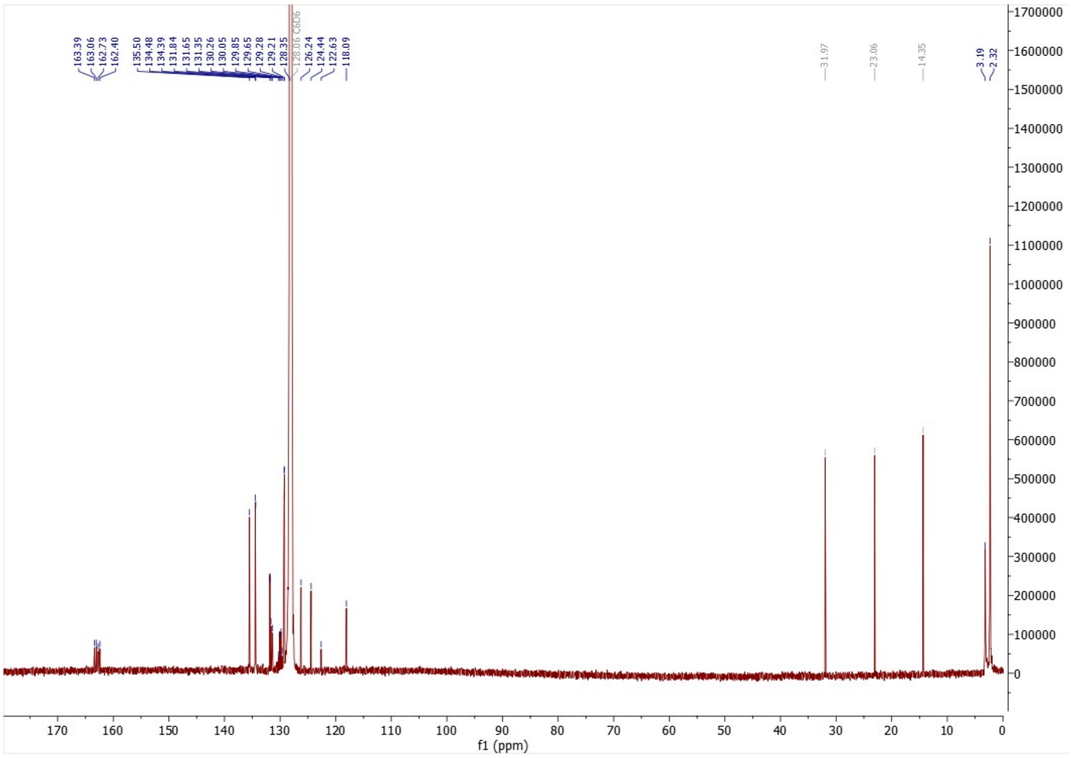

Figure S20. $^{13}C\{^{1}H\}$ NMR spectrum (C_6D_6, 298 K, 151 MHz) of $[RhH(PPh_3)\{Ge_9(Hyp)_3\}][BAr^F{}_4]$ (**2b**$[BAr^F{}_4]$).

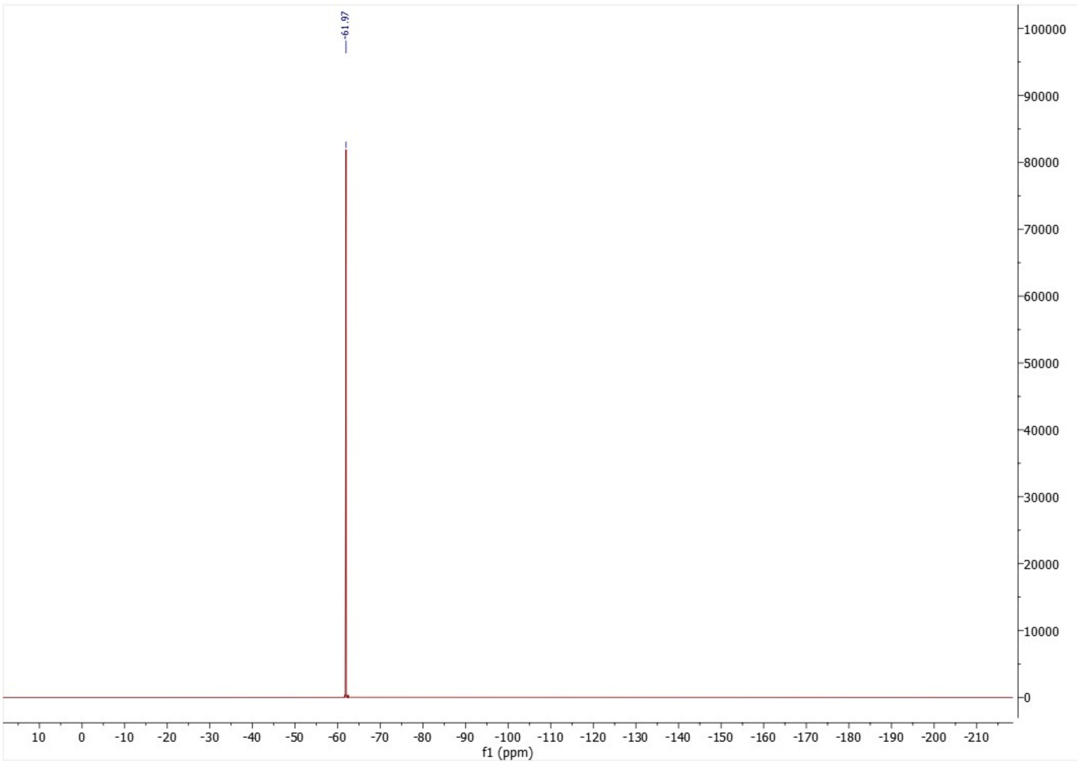

Figure S21. $^{19}F\{^1H\}$ NMR spectrum (C_6D_6, 298 K, 376.53 MHz) of [RhH(PPh_3){Ge_9(Hyp)$_3$}][BAr$^F_4$] (**2b**[BAr$^F_4$]).

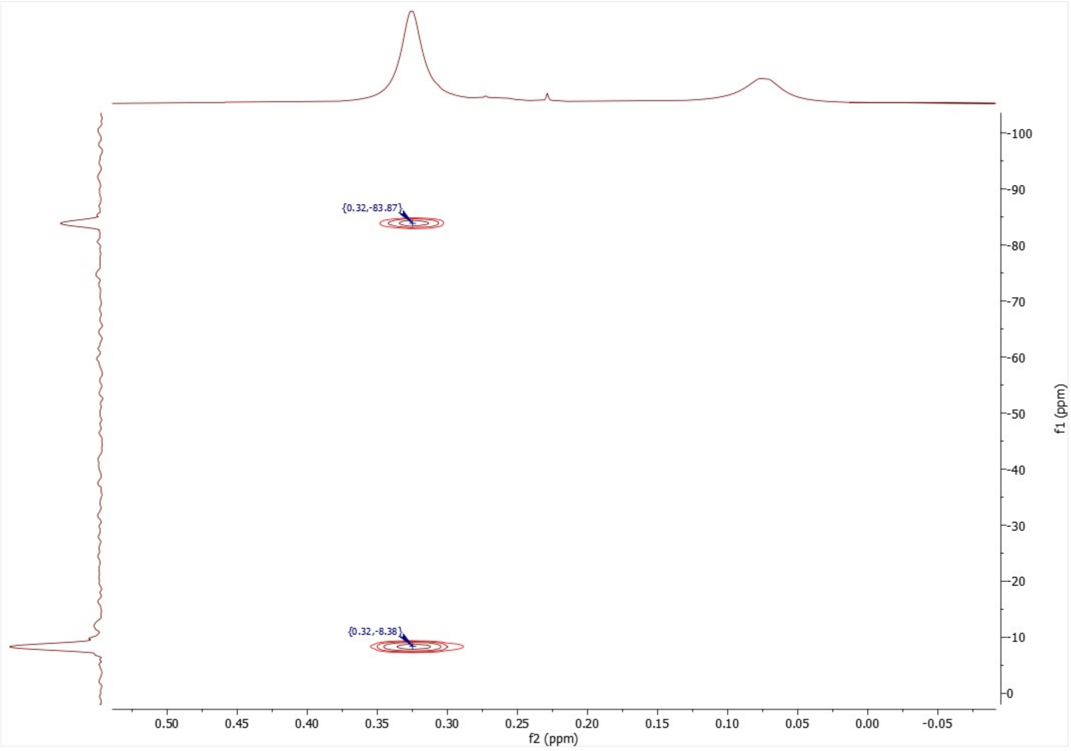

Figure S22. $^1H/^{29}Si$ HMBC NMR spectrum (C_6D_6, 298 K, 79.5 MHz) of [RhH(PPh_3){Ge_9(Hyp)$_3$}][BAr$^F_4$] (**2b**[BAr$^F_4$]).

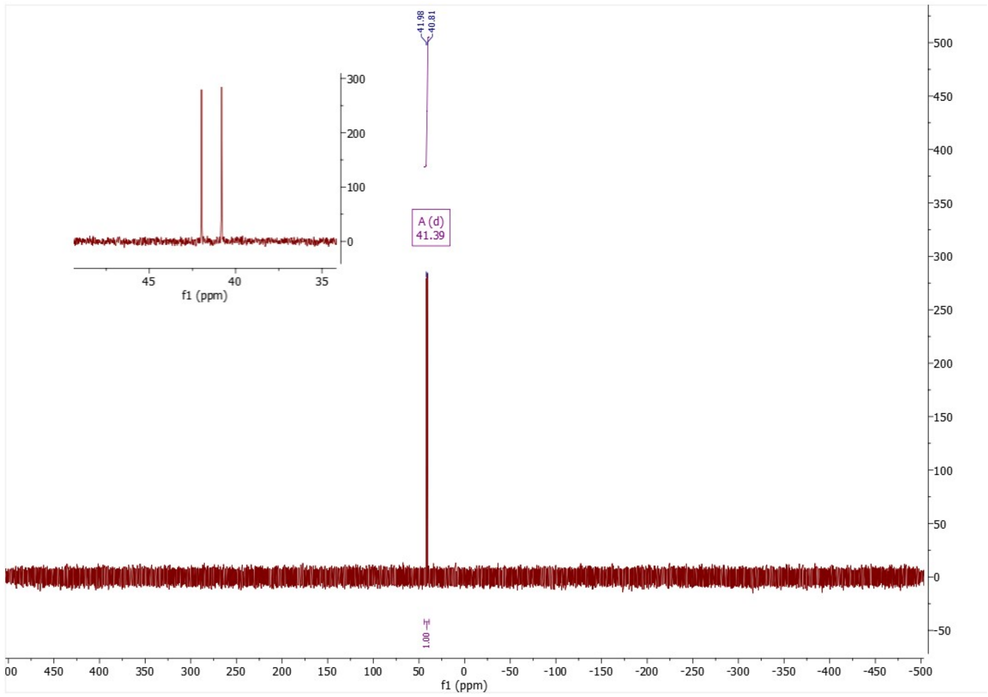

Figure S23. $^{31}P\{^{1}H\}$ NMR spectrum (C_6D_6, 298 K, 162 MHz) of $[RhH(PPh_3)\{Ge_9(Hyp)_3\}][BAr^F_4]$ (**2b**$[BAr^F_4]$).

1.6. NMR spectra for [RhH{Ge_9(Hyp)$_3$}(μ-H)$_2$W(Cp)$_2$][BAr$^F_4$] (**2d**[BAr$^F_4$]).

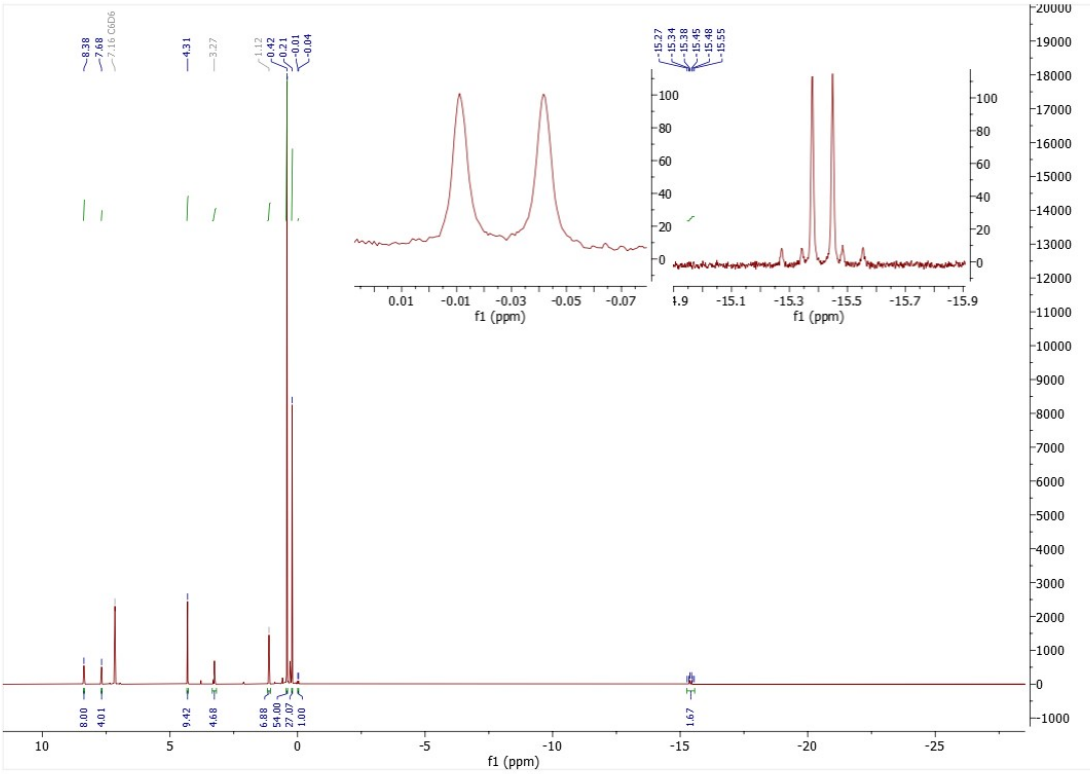

Figure S24. ^{1}H NMR spectrum (C_6D_6, 298 K, 400 MHz) of [RhH{Ge_9(Hyp)$_3$}(μ-H)$_2$W(Cp)$_2$][BAr$^F_4$] (**2d**[BAr$^F_4$]).

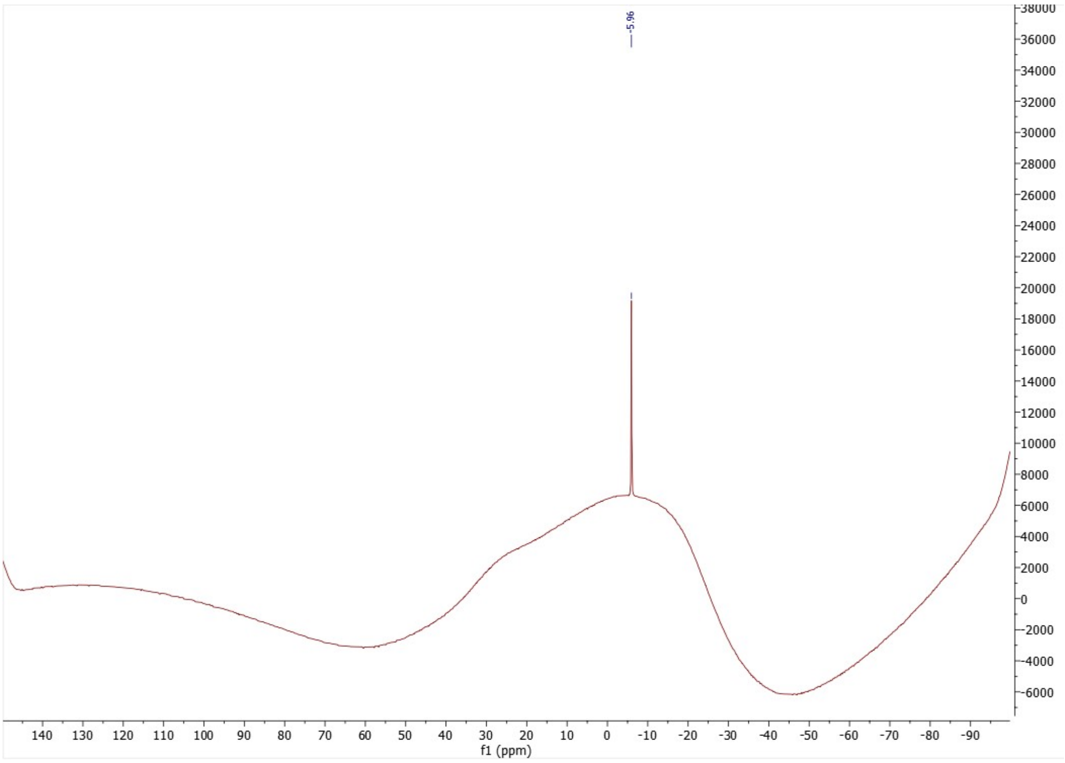

Figure S25. ^{11}B{^{1}H} NMR spectrum (C_6D_6, 298 K, 128.40 MHz) of [RhH{Ge_9(Hyp)$_3$}(μ-H)$_2$W(Cp)$_2$][BAr$^F_4$] (**2d**[BAr$^F_4$]).

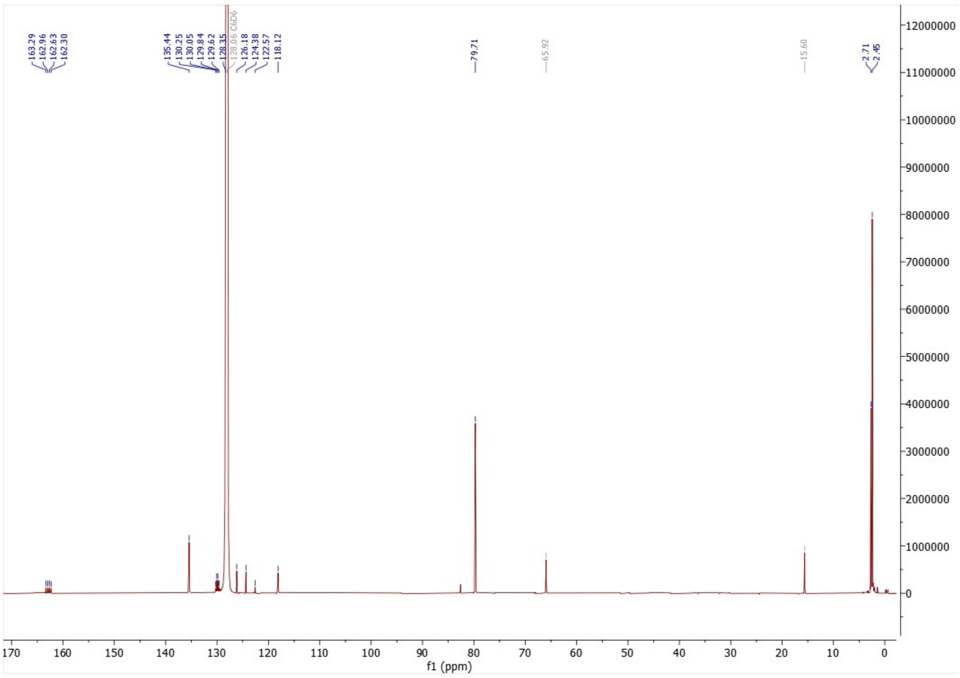

Figure S26. $^{13}C\{^{1}H\}$ NMR spectrum (C_6D_6, 298 K, 151 MHz) of $[RhH\{Ge_9(Hyp)_3\}(\mu\text{-}H)_2W(Cp)_2][BAr^F{}_4]$ (**2d**$[BAr^F{}_4]$).

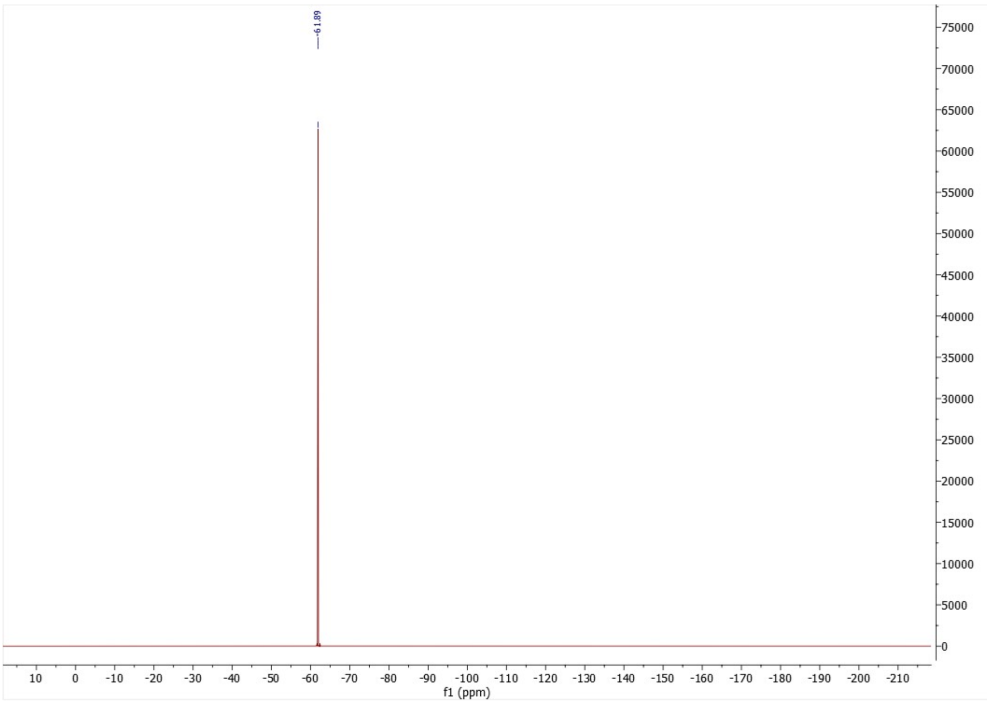

Figure S27. $^{19}F\{^{1}H\}$ NMR spectrum (C_6D_6, 298 K, 376.53 MHz) of $[RhH\{Ge_9(Hyp)_3\}(\mu\text{-}H)_2W(Cp)_2][BAr^F{}_4]$ (**2d**$[BAr^F{}_4]$).

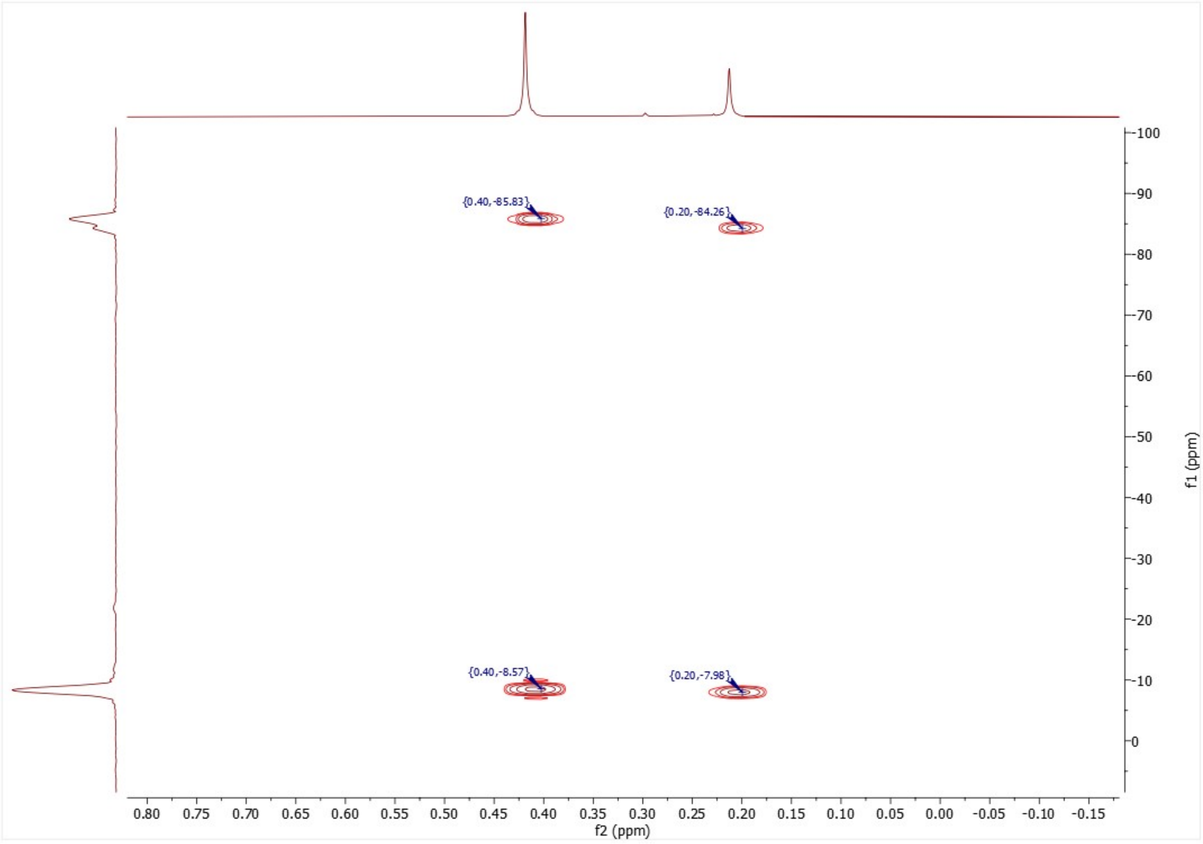

Figure S28. $^1H/^{29}Si$ HMBC NMR spectrum (C_6D_6, 298 K, 79.51 MHz) of $[RhH\{Ge_9(Hyp)_3\}(\mu\text{-}H)_2W(Cp)_2][BAr^F_4]$ (**2d**$[BAr^F_4]$).

1.7. NMR spectra for [Rh{Ge_9(Hyp)$_3$}(μ-D)$_2$W(Cp)$_2$] (d_2-**1d**).

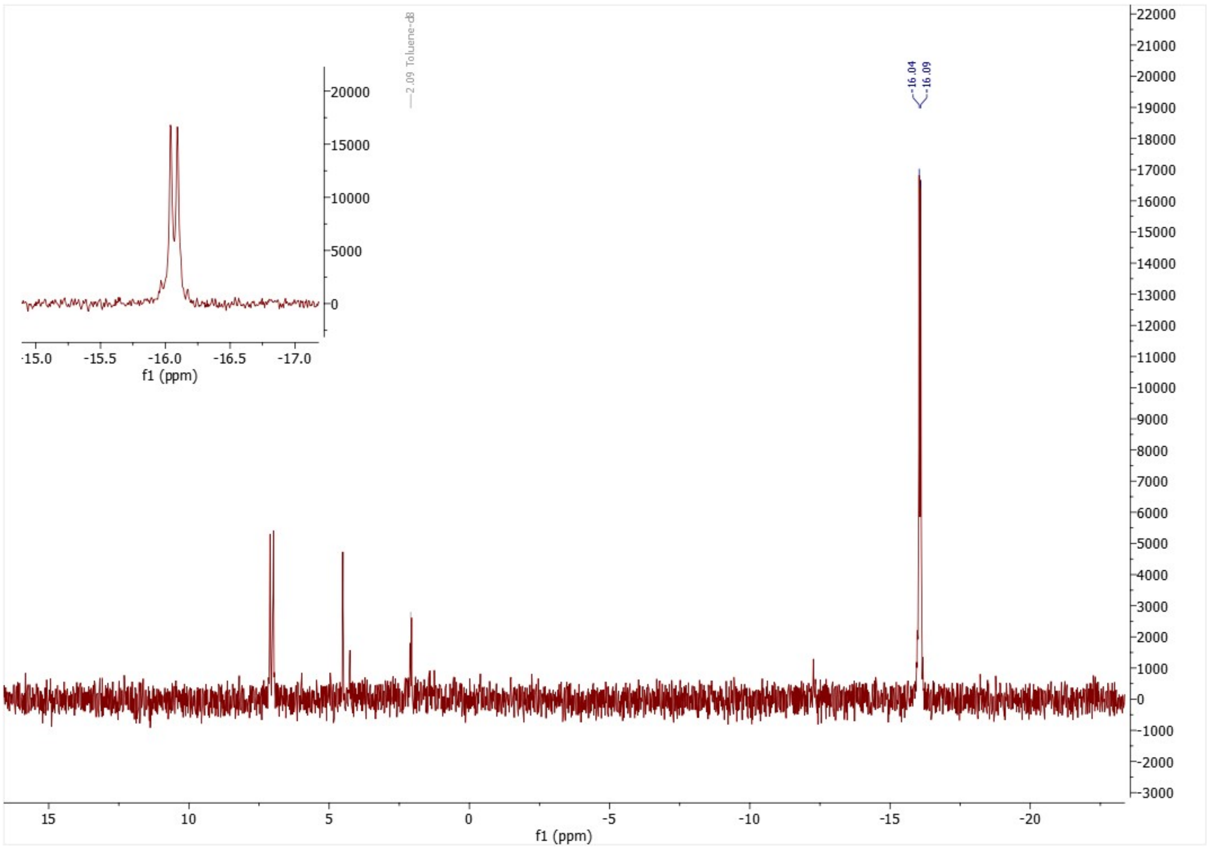

Figure S29. ^{2}H NMR spectrum (C_7H_8, 298 K, 76.74 MHz) of [Rh{Ge_9(Hyp)$_3$}(μ-D)$_2$W(Cp)$_2$] (d_2-**1d**).

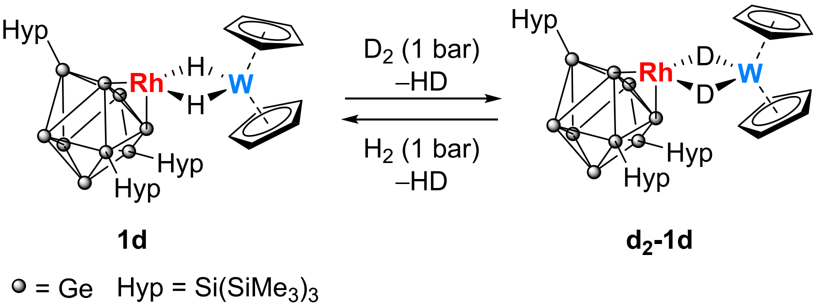

Scheme S1: Reversible formation of [Rh{Ge_9(Hyp)$_3$}(μ-D)$_2$W(Cp)$_2$] (d_2-**1d**).

To test bimetallic reactivity, a C_6D_6 solution of compound **1d** was placed under an atmosphere of D_2 (1 bar). The doublet ^{1}H NMR signal for the bridging hydrides immediately started to decrease in intensity and dissolved H_2 and HD were observed.

The reaction was mixed by tumbling for 3 hours, resulting in a low intensity peak containing two overlapping doublet resonances. This is consistent with a mixture of compound **1d**, an isotopically shifted hydrido-deuteride complex [Rh{Ge_9($Hyp)_3$}(μ-H)(μ-D)W($Cp)_2$] and the unobserved dideuteride complex [Rh{Ge_9($Hyp)_3$}(μ-$D)_2$W($Cp)_2$] (d_2-**1d**). Replacing the atmosphere with H_2 resulted in complete regeneration of compound **1d** and the observation of dissolved HD over the course of a day.

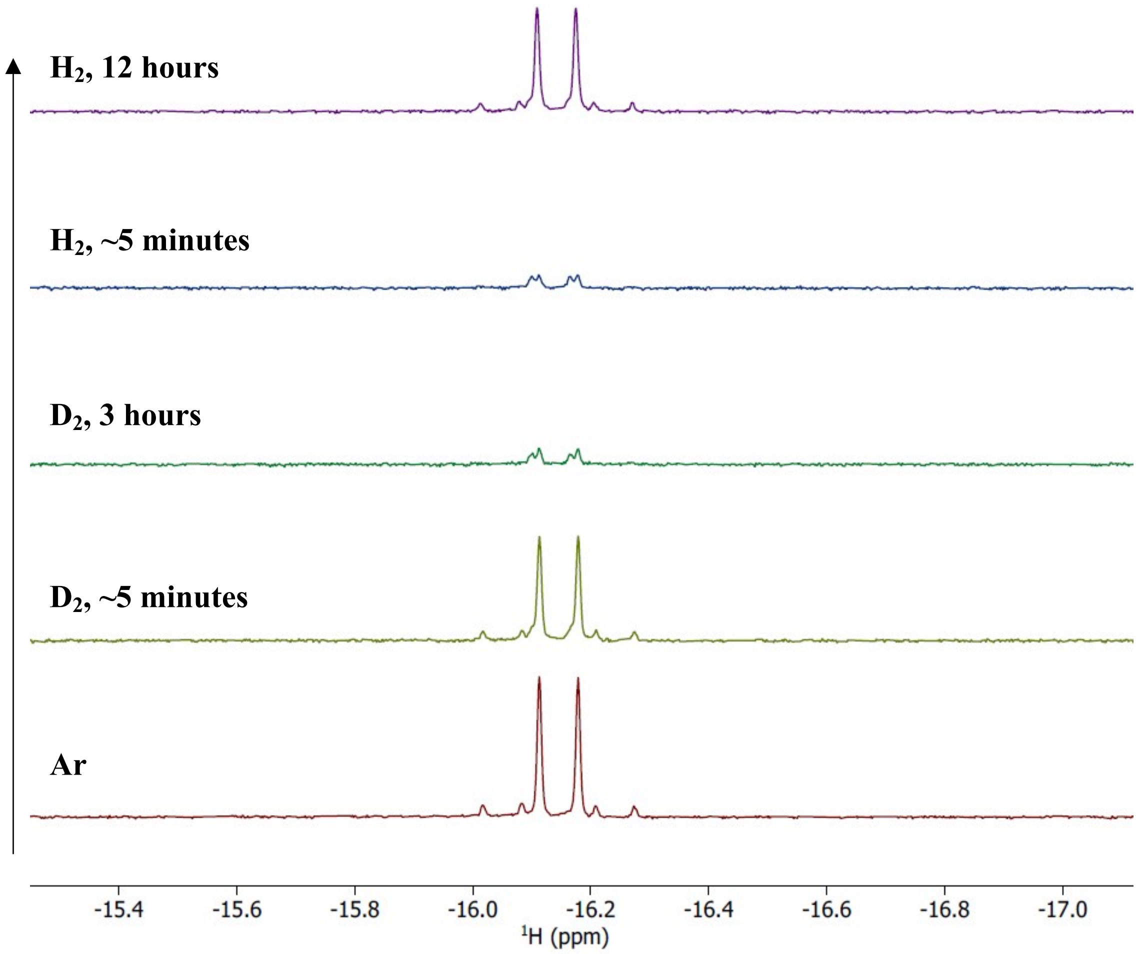

Figure S30. ^{1}H NMR spectra (C_6D_6, 298 K, 400.16 MHz) of the bridging hydride showing the reversible activation of H_2 and D_2 on changing the atmosphere. From bottom to top: Ar, D_2, D_2 (3 hours tumbled), H_2, H_2 (12 hours tumbled).

2. X-ray Crystallography

Single-crystal X-ray diffraction data were collected using an Oxford Diffraction Supernova dual-source diffractometer equipped with a 135 mm Atlas CCD area detector. Crystals were selected under Paratone-N oil, mounted on micromount loops and quench-cooled using an open flow N_2 cooling device.[1] Data were collected at 150 K using mirror monochromated Cu Kα (λ = 1.54184 Å) radiation and processed using the CrysAlisPro package, including unit cell parameter refinement and inter-frame scaling (which was carried out using SCALE3 ABSPACK within CrysAlisPro).[2] Equivalent reflections were merged and diffraction patterns processed with the CrysAlisPro suite. Structures were subsequently solved using direct methods and refined using the SHELXL package.[3] Hydrogen atoms, with the exception of metal hydrides, were typically included in calculated positions (riding model). Crystal data, details of data collections and refinements for all structures can be found in the CIF files and are summarized in Tables S1 and S2.

Table S1. Selected X-ray data collection and refinement parameters for **1a**, **1c**·hexane and **1d**.

	**1a**	**1c**·hexane	**1d**
Formula	$C_{30}H_{90}Ge_9PRhSi_{12}$	$C_{40}H_{107}Ge_9N_2RhSi_{12}$	$C_{37}H_{93}Ge_9RhSi_{12}W$
CCDC	2155445	2155446	2155447
Fw [g mol^{-1}]	1575.28	1709.57	1815.26
Crystal system	monoclinic	triclinic	monoclinic
Space group	$P2_1/c$	P−1	$P2_1/c$
a (Å)	14.4411(1)	16.3622(3)	14.27830(10)
b (Å)	25.5519(2)	19.8996(3)	25.64300(10)
c (Å)	18.9046(1)	25.9438(4)	19.84610(10)
α (°)	90	68.560(1)	90
β (°)	101.771(1)	79.856(1)	101.575(1)
γ (°)	90	82.113(1)	90
V (Å^3)	6829.06(8)	7715.4(2)	7118.64(7)
Z	4	4	4
Radiation, λ (Å)	Cu Kα, 1.54184	Cu Kα, 1.54184	Cu Kα, 1.54184
Temp (K)	150(2)	150(2)	150(2)
ρ_{calc} (g cm^{-3})	1.532	1.472	1.694
μ (mm^{-1})	8.686	7.552	11.032
Reflections collected	132946	141405	196412
Independent reflections	14310	32320	14900
Parameters	569	1332	668
R(int)	0.0521	0.0488	0.0545
R1/wR2,[a] I ≥ 2σI (%)	3.93/9.75	5.44/16.41	2.44/6.23
R1/wR2,[a] all data (%)	4.56/10.20	5.93/17.28	2.52/6.29
GOF	1.019	1.048	1.054

$R1 = [\Sigma||F_o| - |F_c||]/\Sigma|F_o|$; $wR2 = \{[\Sigma w[(F_o)^2 - (F_c)^2]^2]/[\Sigma w(F_o^2)^2\}^{1/2}$; $w = [\sigma^2(F_o)^2 + (AP)^2 + BP]^{-1}$, where $P = [(F_o)^2 + 2(F_c)^2]/3$ and the A and B values are 0.0442 and 15.49 for **1a**, 0.1167 and 1.9 for **1c**·hexane, and 0.0332 and 7.57 for **1d.**

Table S2. Selected X-ray data collection and refinement parameters for **2b**[BAr$^F{}_4$]·1.5hex and **2d**[BAr$^F{}_4$]·Et$_2$O.

	**2b**[BAr$^F{}_4$]·1.5hex	**2d**[BAr$^F{}_4$]·Et$_2$O
Formula	$C_{86}H_{130}BF_{24}Ge_9PRhSi_{12}$	$C_{73}H_{116}BF_{24}Ge_9ORhSi_{12}W$
CCDC	2155448	2155449
Fw [g mol^{-1}]	2754.97	2753.61
Crystal system	triclinic	monoclinic
Space group	*P*−1	$P2_1/c$
a (Å)	15.0839(2)	17.9023(5)
b (Å)	19.3502(3)	13.8042(5)
c (Å)	21.6497(4)	44.4206(12)
α (°)	106.313(1)	90
β (°)	96.757(1)	96.969(3)
γ (°)	92.771(1)	90
V (Å^3)	6000.51(17)	10896.4(6)
Z	2	4
Radiation, λ (Å)	Cu Kα, 1.54184	Cu Kα, 1.54184
Temp (K)	150(2)	150(2)
ρ_{calc} (g cm^{-3})	1.525	1.679
μ (mm^{-1})	5.511	7.778
Reflections collected	89656	101192
Independent reflections	24711	9244
Parameters	1332	1312
R(int)	0.0439	0.1242
R1/wR2,[a] I ≥ 2σI (%)	3.44/8.94	6.47/16.56
R1/wR2,[a] all data (%)	4.02/9.36	9.10/18.38
GOF	1.022	1.058

$R1 = [\Sigma||F_o| - |F_c||]/\Sigma|F_o|$; $wR2 = \{[\Sigma w[(F_o)^2 - (F_c)^2]^2]/[\Sigma w(F_o{}^2)^2\}^{1/2}$; $w = [\sigma^2(F_o)^2 + (AP)^2 + BP]^{-1}$, where $P = [(F_o)^2 + 2(F_c)^2]/3$ and the A and B values are 0.0518 and 2.02 for **2b**[BAr$^F{}_4$]·1.5hex, and 0.0908 and 0.00 for **2c**[BAr$^F{}_4$]·Et$_2$O**.**

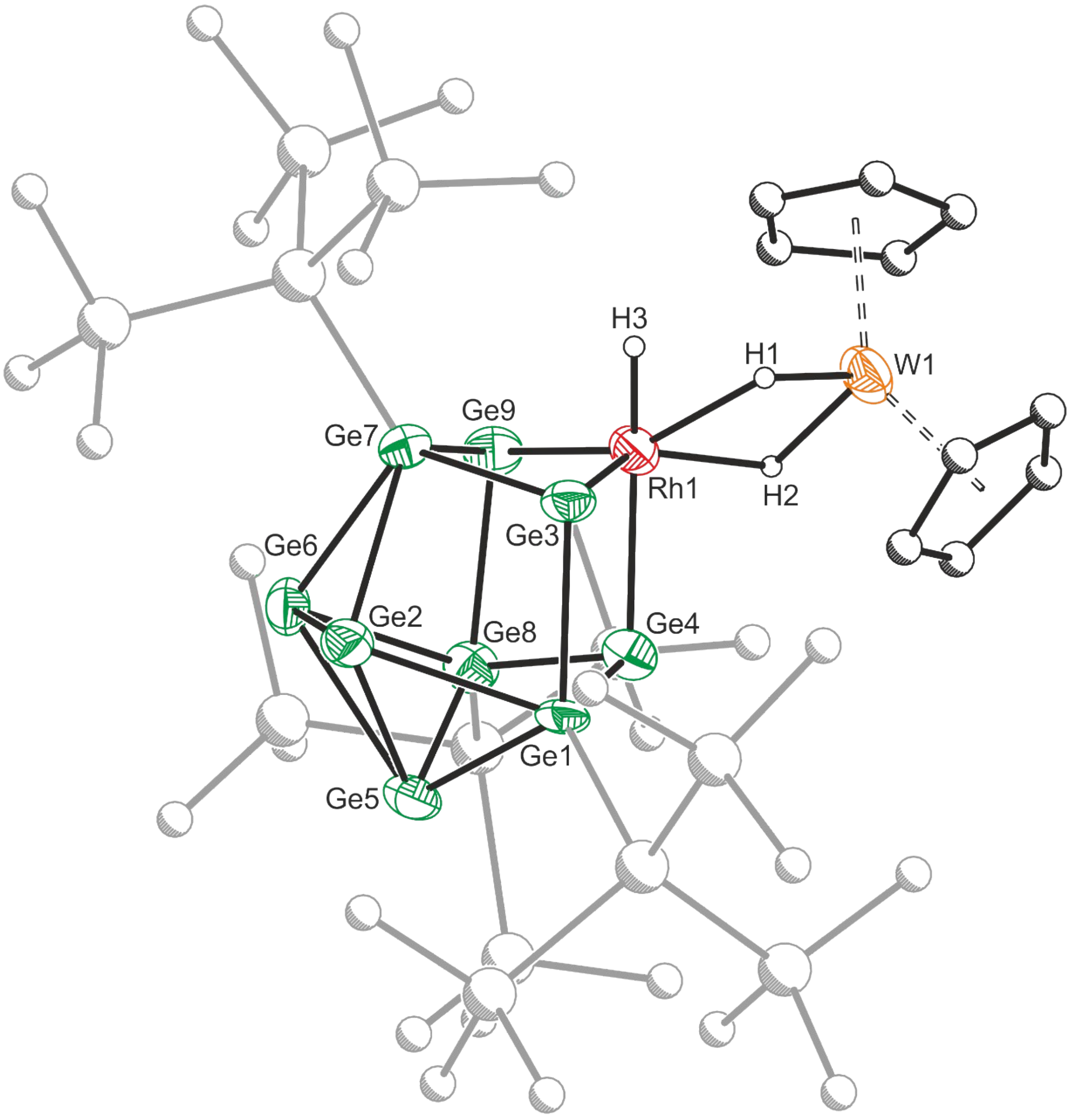

Figure S31. Molecular structure of the cationic portion of **2d**[BAr$^{F}{}_{4}$]·Et$_2$O. Anisotropic displacement ellipsoids are set at 50% probability. Only the major disordered component is shown.

3. Catalysis Procedures

3.1. H/D Scrambling

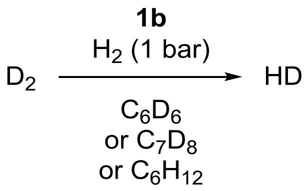

Scheme S2. H/D scrambling of a H_2/D_2 mixture in the presence of **1b**.

Compound **1b** (6 mg, 0.0034 mmol) was added to an NMR tube with a J. Young stopcock and dissolved in either C_6D_6, C_7D_8 or cyclohexane. This was freeze-pump-thaw degassed three times before charging with D_2 (0.5 bar) and stirring overnight to saturate the solution. The solution was then frozen, and partially degassed (~10^2 mbar) before charging with H_2 (1 bar). HD formation was observed within 5 minutes of the H_2 addition by ^{1}H NMR spectroscopy.

A Hg drop test was used to discount the formation of a colloidal catalyst using the following method: Compound **1b** (6 mg, 0.0034 mmol) was added to an ampoule with a J. Young stopcock and dissolved in C_6H_6 (0.5 ml). This was freeze-pump-thaw degassed three times before charging with H_2 (0.5 bar) and stirred for 5 minutes before transferring via cannula onto Hg (0.5 g) and pressurizing with D_2 (1 bar). This was stirred for 10 minutes before transferring the headspace into an evacuated NMR tube containing degassed C_6D_6. The NMR tube was vigorously shaken to saturate the solution with the headspace atmosphere. Dissolved H_2 and HD was observed by ^{1}H NMR spectroscopy.

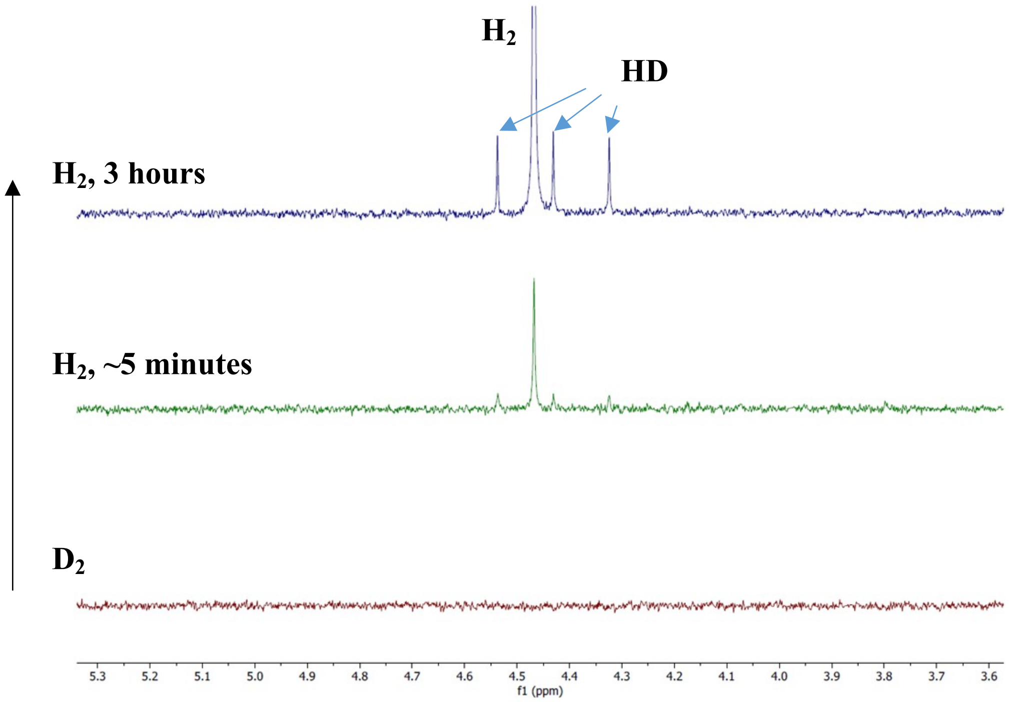

Figure S32. ^{1}H NMR spectra (C_6D_6, 298 K, 400 MHz) to show HD formation from H_2 and D_2 in the presence of **1b**. Bottom to top: D_2 atmosphere, immediately after adding H_2, 3 hours standing.

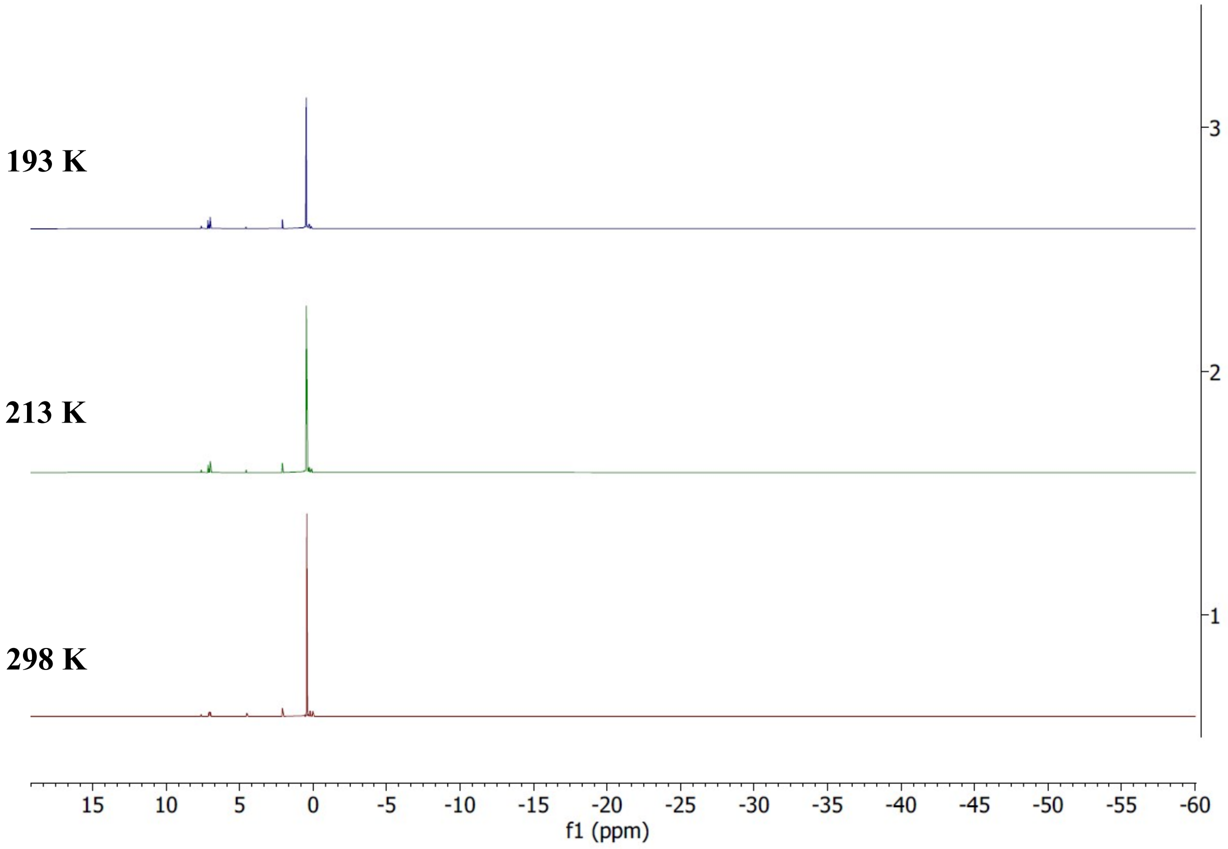

Figure S33. ^{1}H NMR spectra (C_7D_8, 298 K, 500 MHz) to show no observable change or dihydride formation when **1b** is charged with H_2. Bottom to top: 298 K, 213 K, 193 K.

3.2. H/D Scrambling with Alkenes

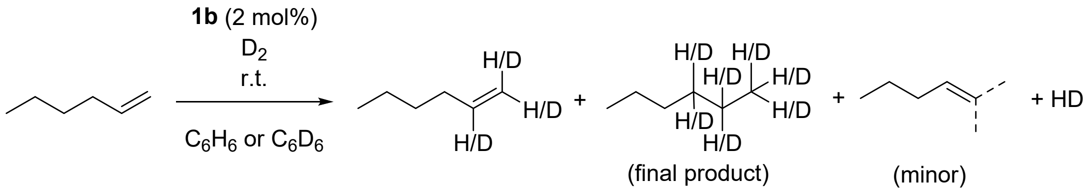

Scheme S3. H/D scrambling and hydrogenation of 1-hexene in under D_2 the presence of **1b**.

Compound **1b** (3 mg, 0.0017 mmol) was added to was added to an NMR tube with a J. Young stopcock and dissolved in either C_6H_6 or C_6D_6. 1-hexene (10 µl, 0.08 mmol) was added using a micro-syringe. The solution was freeze-pump-thaw degassed three times before charging with D_2 (1 bar) and constantly mixed by tumbling. The products were identified by ^1H (C_6D_6 solution) or ^2H (C_6H_6 solution) NMR spectroscopy. After recharging the system with D_2, the deuterated hexenes converged to deuterated hexane.

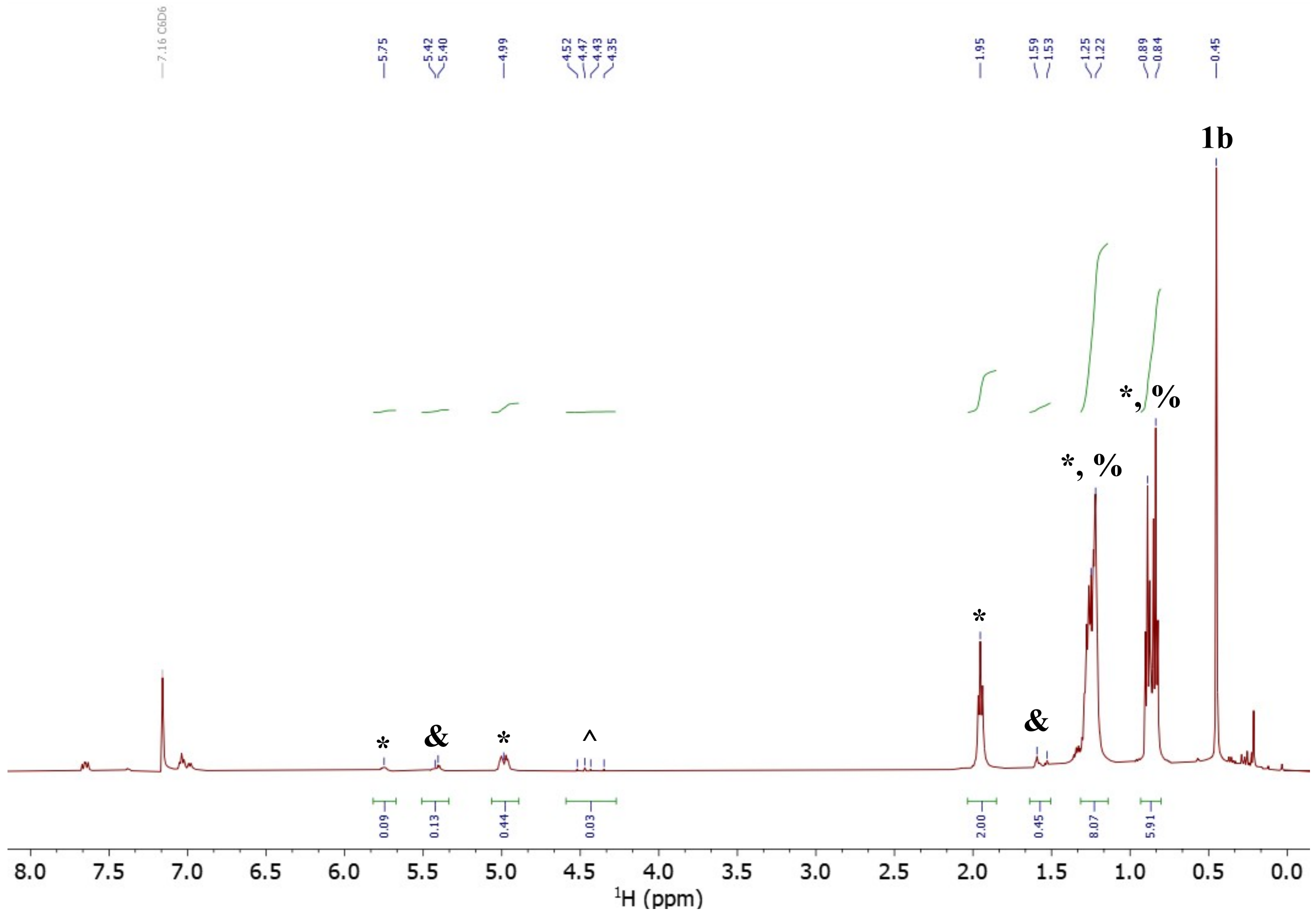

Figure S34. ^{1}H NMR spectra (C_6D_6, 298 K, 500 MHz) to show the decrease in intensity at the alkenic positions when 1-hexene is charged with D_2 in the presence of **1b**, relative to the two vinylic protons (1.95 ppm). Of particular note is the relative integrations of the CH_2 (4.99 ppm) and CH (5.76 ppm) positions, showing selectivity for H/D exchange at the CH position. Assignments: ***** = 1-hexene, **&** = 2-hexenes, ^ = H_2, D_2, **%** = *n*-hexane.

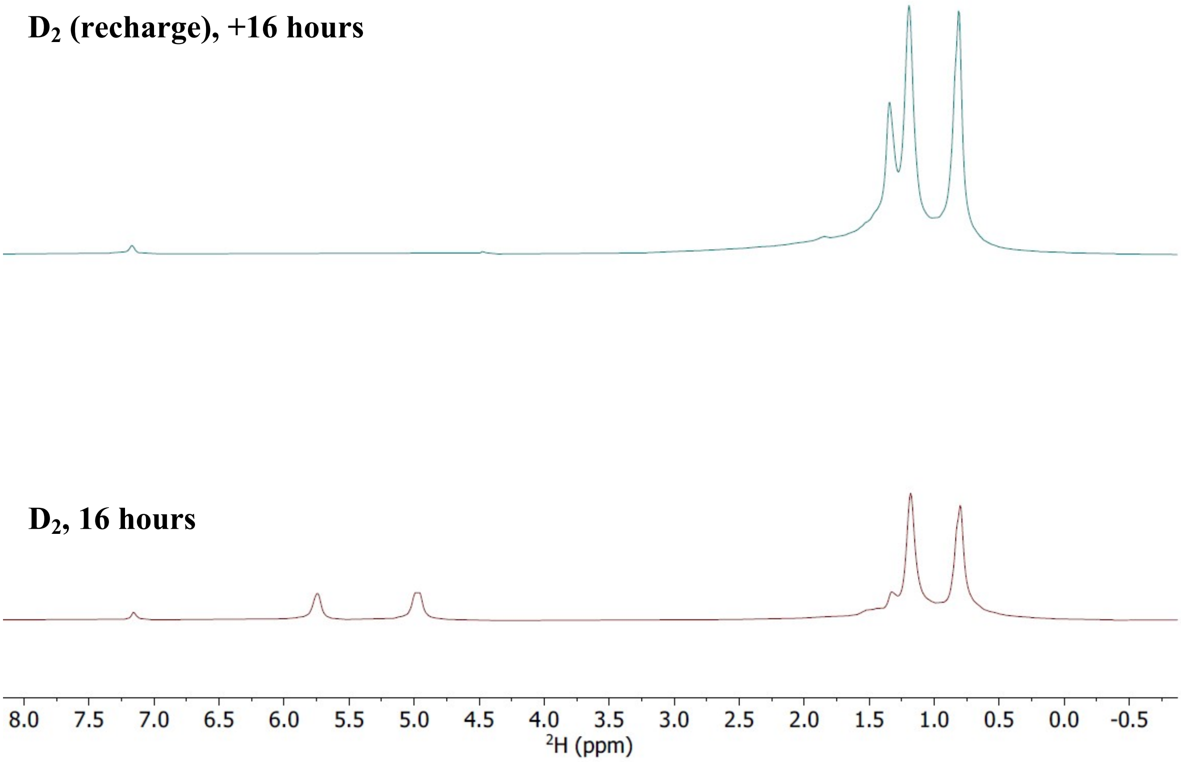

Figure S35. ^{2}H NMR spectrum (C_6H_6, 298 K, 76.74 MHz) of a parallel reaction in C_6H_6 confirming H/D exchange at the alkenic positions, in addition to hydrogenation/deuteration to *n*-hexane (bottom). On recharging the atmosphere with D_2, full hydrogenation/deuteration is observed.

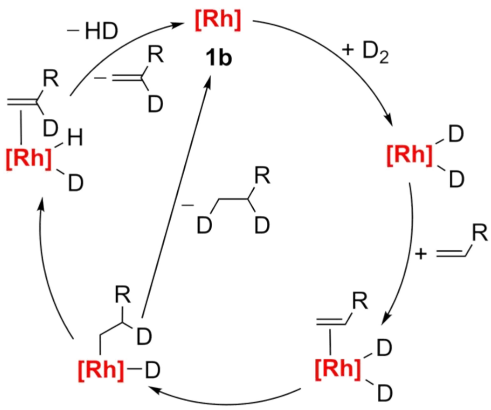

Scheme S4. Proposed catalytic cycle for H/D scrambling and hydrogenation of 1-hexene in under D_2 the presence of **1b**.

4. Computational Details

4.1. Methodology

The geometry optimizations, PCM solvent corrections and frequency calculations were run with Gaussian 16 Revision A.03[4] and performed using the BP86[5,6] functional. Rh, Ge and Si centres were described with Stuttgart RECPs and associated basis sets,[7] with added f-orbital polarization on Rh (ζ = 1.35),[8] and d-orbital polarization on P (ζ = 0.387) Ge (ζ = 0.230) and Si (ζ = 0.284).[9] 6-31G** basis sets were used for C and H (called BS1).[10,11] Single point energy calculations were performed on the optimised geometries, at the BP86/Def2-TZVP level of theory with added pseudopotential on Rh.[12,13] Stationary points were fully characterized via analytical frequency calculations as either minima (all positive eigenvalues) or transition states (one negative eigenvalue). IRC calculations and subsequent geometry optimizations were used to confirm the minima linked by the transition states. Energies reported in the text are based on the gas-phase free energies and incorporate a correction for dispersion effects using Grimme's D3 parameter set[14] (i.e. BP86-D3) as well as solvation (PCM approach) in benzene.

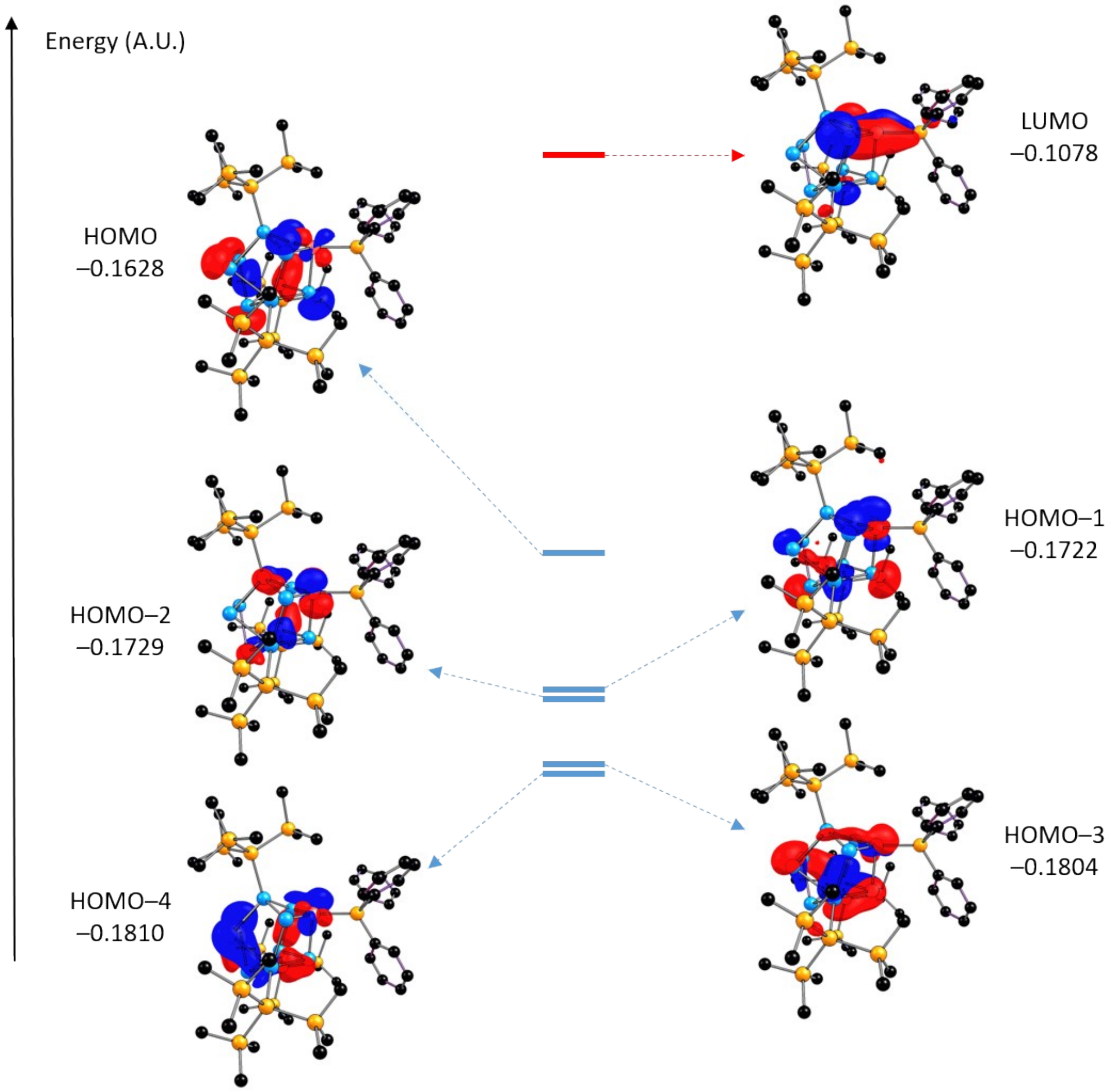

Figure S37. Frontier molecular orbital diagram of **1b** calculated at the BP86/Def2-TZVP level of theory.

4.2. Fluxional Behavior

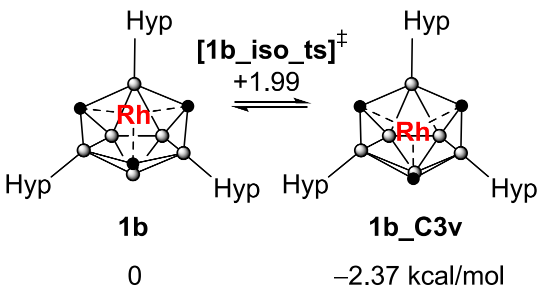

Scheme S6. Isomerization of **1b**, calculated at the BP86-D3/Def2-TZVP level of theory.

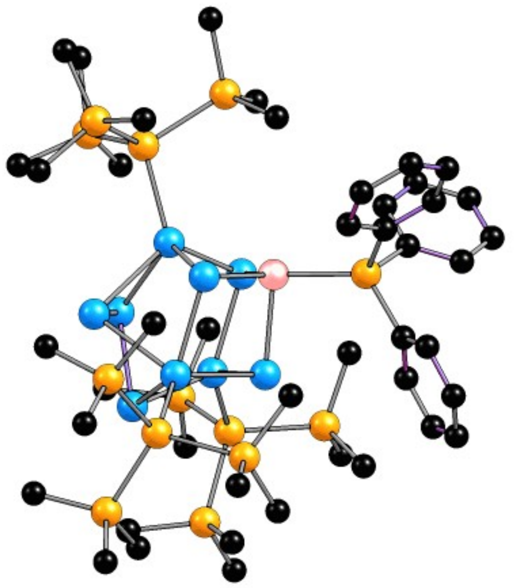

Figure S38. Optimized geometry of **1b** calculated at the BP86/6-31g(d,p)/SDDAll level of theory. Hydrogen atoms attached to carbon atoms omitted for clarity.

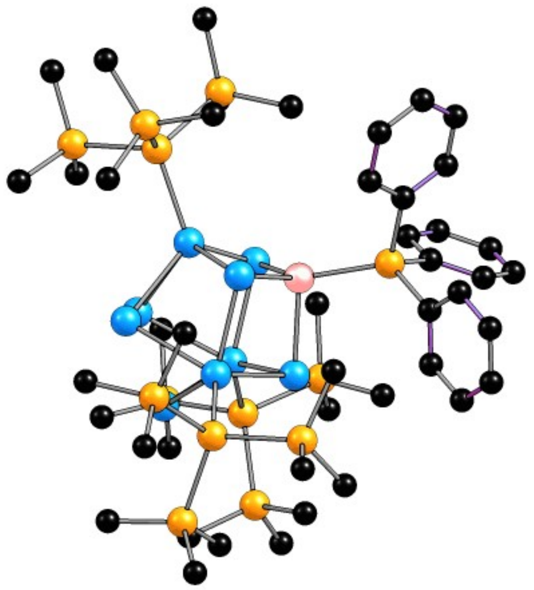

Figure S39. Optimized geometry of **1b_iso_ts** calculated at the BP86/6-31g(d,p)/SDDAll level of theory. Hydrogen atoms attached to carbon atoms omitted for clarity.

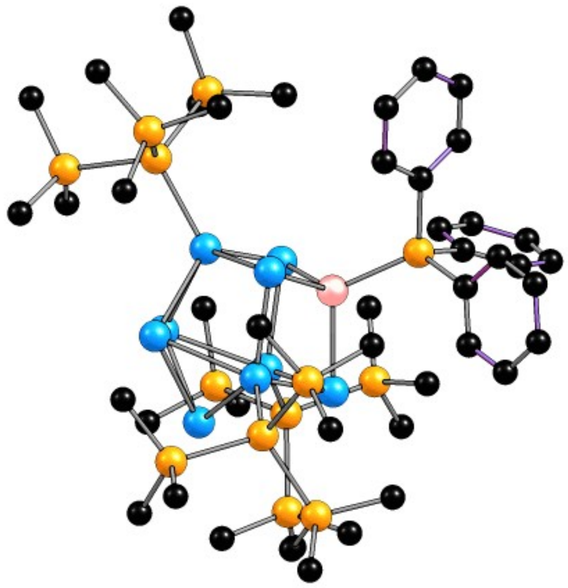

Figure S40. Optimized geometry of **1b_C3v** calculated at the BP86/6-31g(d,p)/SDDAll level of theory. Hydrogen atoms attached to carbon atoms omitted for clarity.

4.3. η^3–η^1 Isomerisation

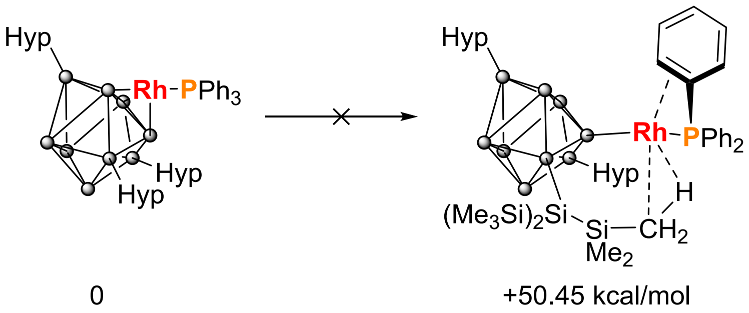

Scheme S7. η^3–η^1 isomerization of **1b**, calculated at the BP86-D3/Def2-TZVP level of theory.

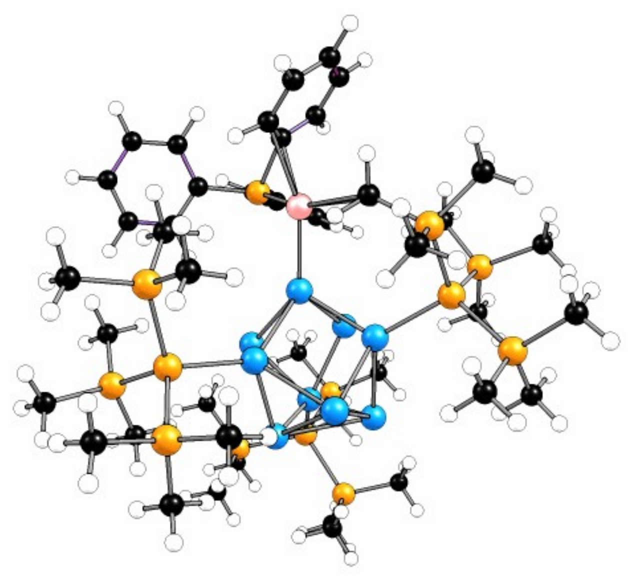

Figure S41. Optimized geometry of **1b_eta1** calculated at the BP86/6-31g(d,p)/SDDAll level of theory.

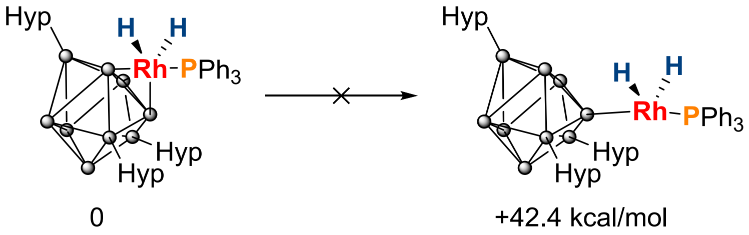

Scheme S8. η^3–η^1 isomerization of **1b_H_H**, calculated at the BP86-D3/Def2-TZVP level of theory.

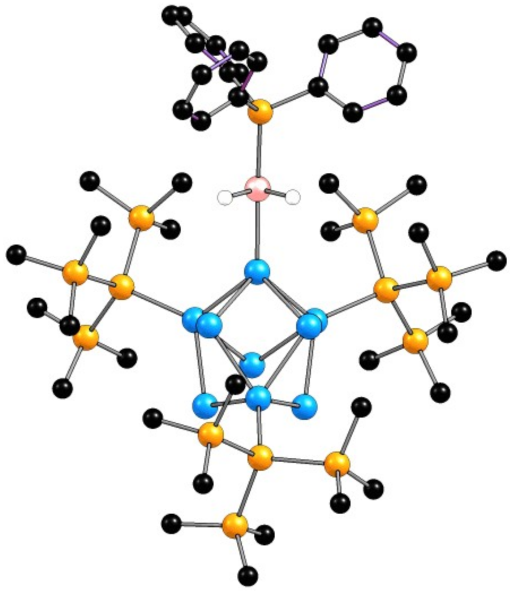

Figure S42. Optimized geometry of **1b_H2_eta1** calculated at the BP86/6-31g(d,p)/SDDAll level of theory. Hydrogen atoms attached to carbon atoms omitted for clarity.

4.4. Phosphine Dissociation

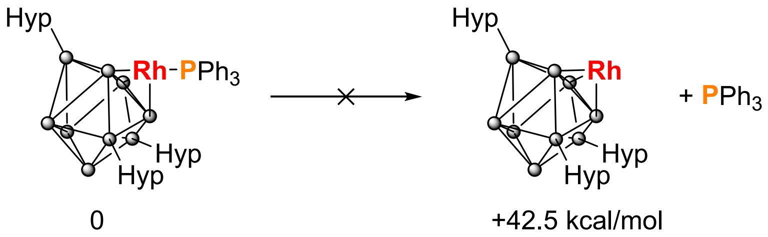

Scheme S9. Phosphine dissociation from **1b**, calculated at the BP86-D3/Def2-TZVP level of theory.

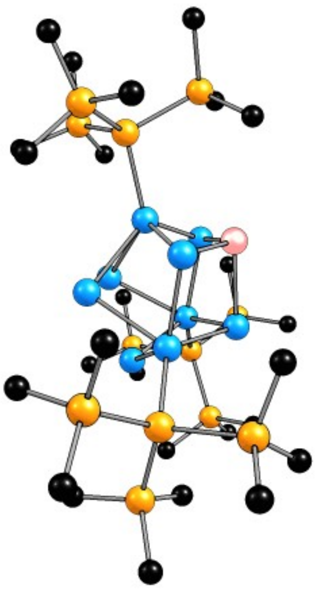

Figure S43. Optimized geometry of **1b_PPh3diss** calculated at the BP86/6-31g(d,p)/SDDAll level of theory. Hydrogen atoms attached to carbon atoms omitted for clarity.

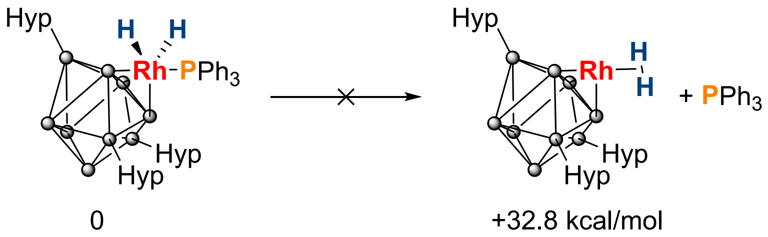

Scheme S10. Phosphine dissociation from **1b_H_H**, calculated at the BP86-D3/Def2-TZVP level of theory.

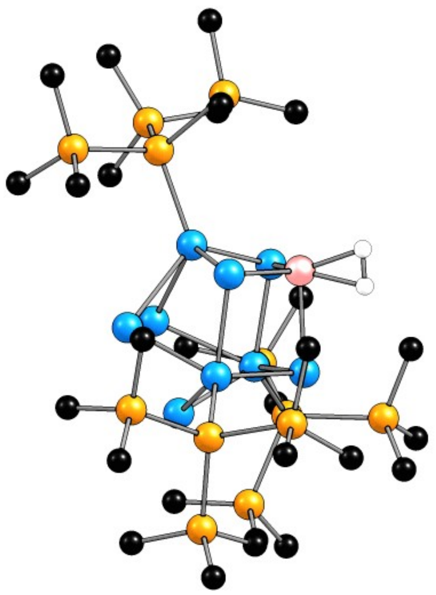

Figure S44. Optimized geometry of **Rh_H2** calculated at the BP86/6-31g(d,p)/SDDAll level of theory. Hydrogen atoms attached to carbon atoms omitted for clarity.

4.5. Oxidative Addition of H_2 at **1b**

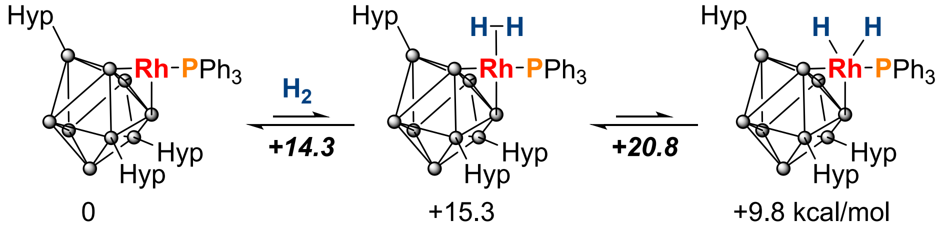

Scheme S11. Oxidative addition of H_2 at **1b**, calculated at the BP86-D3/Def2-TZVP level of theory.

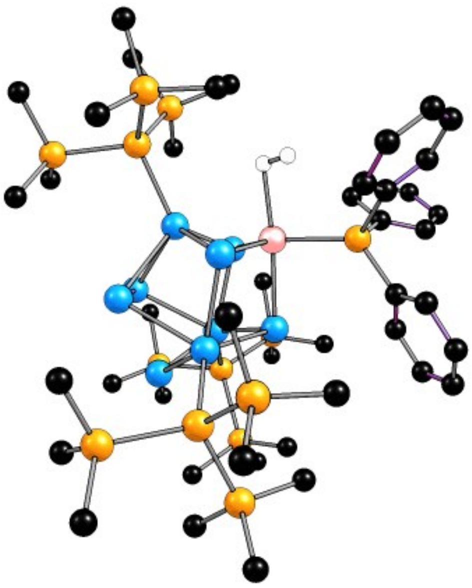

Figure S45. Optimized geometry of **1b_H2_diss_ts** calculated at the BP86/6-31g(d,p)/SDDAll level of theory. Hydrogen atoms attached to carbon atoms omitted for clarity.

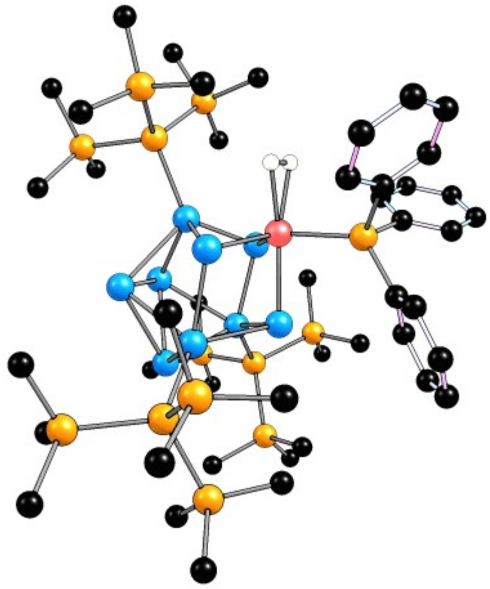

Figure S46. Optimized geometry of **1b_H2** calculated at the BP86/6-31g(d,p)/SDDAll level of theory. Hydrogen atoms attached to carbon atoms omitted for clarity.

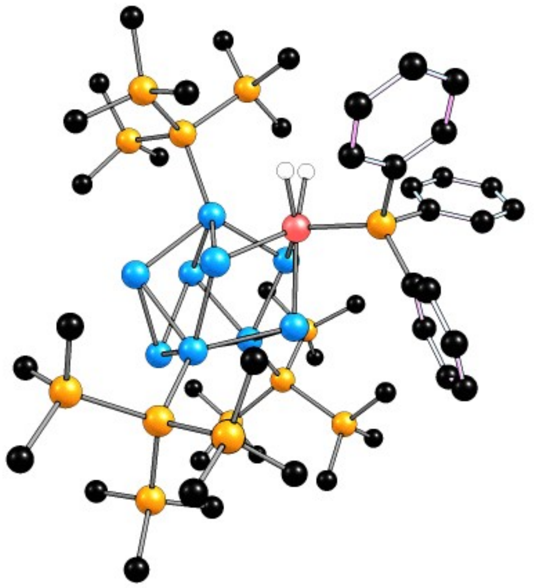

Figure S47. Optimized geometry of **1b_H2_oxadd_ts** calculated at the BP86/6-31g(d,p)/SDDAll level of theory. Hydrogen atoms attached to carbon atoms omitted for clarity.

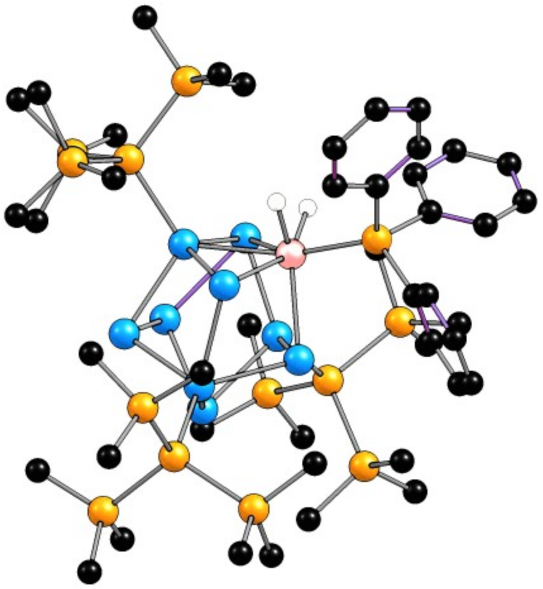

Figure S48. Optimized geometry of **1b_H_H** calculated at the BP86/6-31g(d,p)/SDDAll level of theory. Hydrogen atoms attached to carbon atoms omitted for clarity.

4.6. Oxidative Addition of H_2 at **2b**

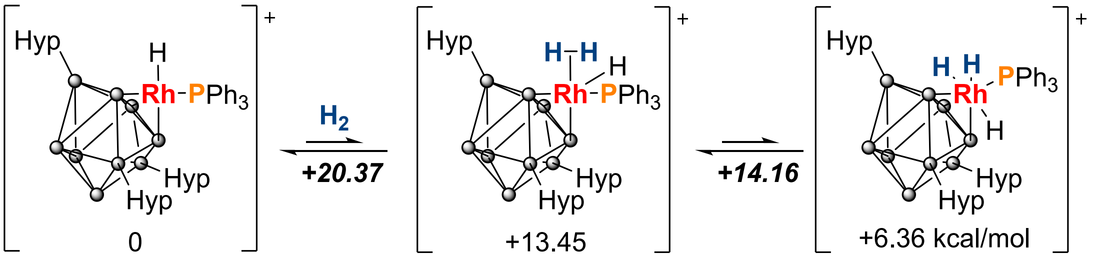

Scheme S12. Oxidative addition of H_2 at **2b**, calculated at the BP86-D3/Def2-TZVP level of theory.

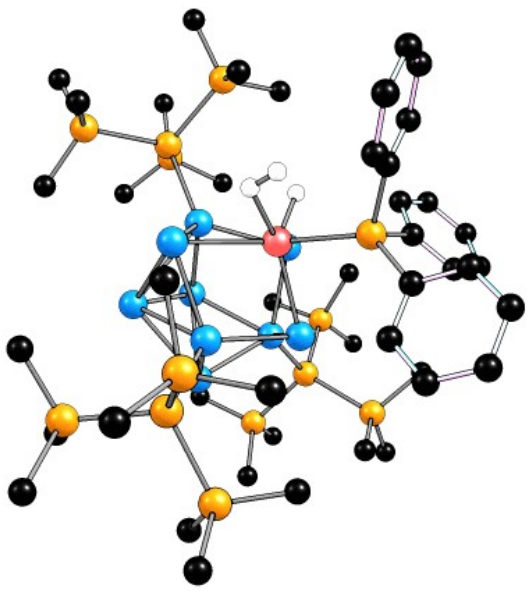

Figure S49. Optimized geometry of **2b_H2_diss_ts** calculated at the BP86/6-31g(d,p)/SDDAll level of theory. Hydrogen atoms attached to carbon atoms omitted for clarity.

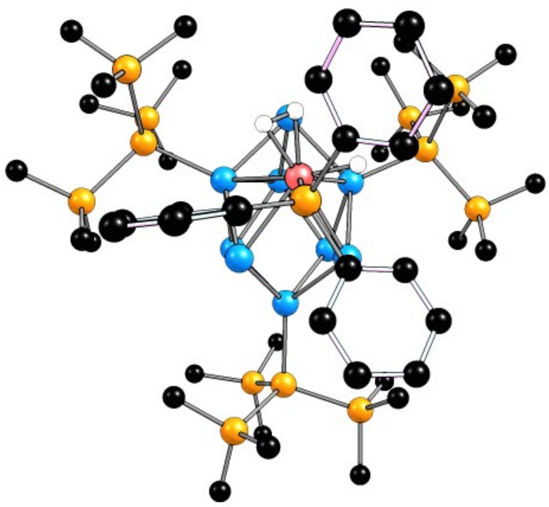

Figure S50. Optimized geometry of **2b_H2** calculated at the BP86/6-31g(d,p)/SDDAll level of theory. Hydrogen atoms attached to carbon atoms omitted for clarity.

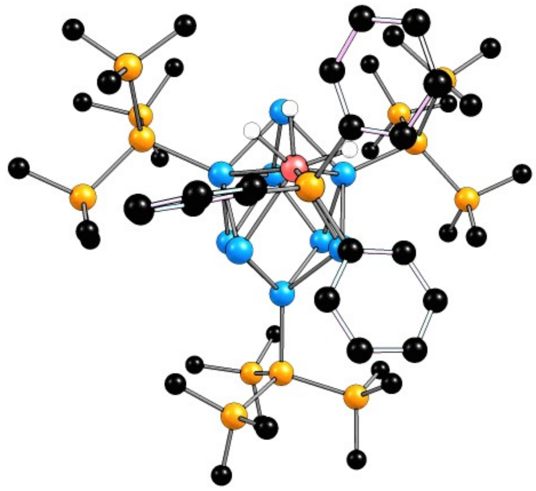

Figure S51. Optimized geometry of **2b_H2_oxadd_ts** calculated at the BP86/6-31g(d,p)/SDDAll level of theory. Hydrogen atoms attached to carbon atoms omitted for clarity.

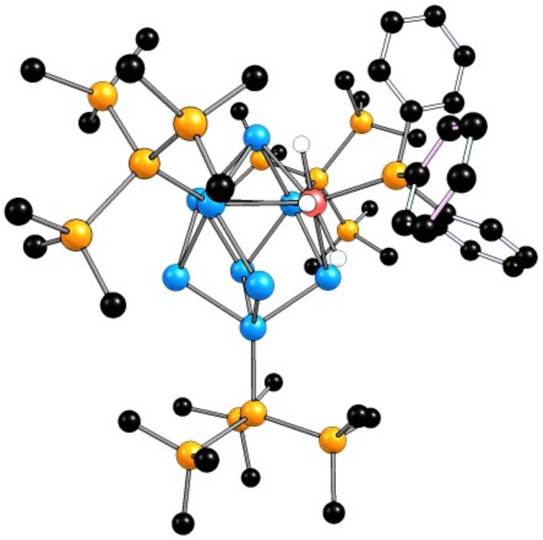

Figure S52. Optimized geometry of **2b_H_H** calculated at the BP86/6-31g(d,p)/SDDAll level of theory. Hydrogen atoms attached to carbon atoms omitted for clarity.

4.7. Computational Appendix

Cartesian Coordinates (Å) of Optimized Geometries and Energies (hartrees) of all Species.

PPh3

```
SCF  =          -701.502629721
H(0 K) =        -701.237101
H(298 K) =      -701.219663
G(298 K) =      -701.284051
SCF+D3 =        -701.538095733
PCM SCF (Benzene) =     -701.505227632
BS2 (def2-tzvp) =       -1036.57409771
Low Freq. = 22.8073cm-1, 24.7885cm-1

P        0.000147000     -0.000103000     -1.251107000
C       -1.129207000     -1.239567000     -0.416237000
C       -0.508733000      1.597479000     -0.415916000
C        1.638087000     -0.358286000     -0.415831000
C        2.472984000     -1.311150000     -1.041407000
C        3.724064000     -1.635758000     -0.495434000
C        4.167165000     -0.997805000      0.675236000
C        3.351248000     -0.039111000      1.297360000
C        2.093521000      0.278551000      0.757806000
H        2.139112000     -1.797967000     -1.965372000
H        4.357922000     -2.379396000     -0.990407000
H        5.147796000     -1.242459000      1.097046000
H        3.693549000      0.465047000      2.207814000
H        1.463094000      1.028265000      1.246792000
C       -0.099524000      2.796907000     -1.040647000
C       -0.443603000      4.042680000     -0.494538000
C       -1.218821000      4.107540000      0.675361000
C       -1.642663000      2.921683000      1.296569000
C       -1.289187000      1.673586000      0.756936000
```

H	0.489909000	2.751147000	-1.964034000
H	-0.115402000	4.963393000	-0.988845000
H	-1.497077000	5.079154000	1.097213000
H	-2.251373000	2.966151000	2.206386000
H	-1.624373000	0.752807000	1.245202000
C	-2.373358000	-1.483170000	-1.040056000
C	-3.280436000	-2.403927000	-0.494134000
C	-2.948505000	-3.109300000	0.674674000
C	-1.708859000	-2.885119000	1.294973000
C	-0.804408000	-1.955116000	0.755531000
H	-2.628884000	-0.948520000	-1.962564000
H	-4.242531000	-2.578682000	-0.987691000
H	-3.651064000	-3.835895000	1.096448000
H	-1.442609000	-3.435755000	2.203921000
H	0.161148000	-1.786438000	1.243232000

H2
SCF = -1.17646513430
H(0 K) = -1.166541
H(298 K) = -1.163237
G(298 K) = -1.178048
SCF+D3 = -1.17646516273
PCM SCF (Benzene) = -1.17652523211
BS2 (def2-tzvp) = -1.17758514685
Low Freq. = 4356.1179cm-1

H	0.000000000	0.000000000	0.375260000
H	0.000000000	0.000000000	-0.375260000

1b
SCF = -1972.04904032
H(0 K) = -1970.786845
H(298 K) = -1970.664346
G(298 K) = -1970.967735
SCF+D3 = -1972.35054229
PCM SCF (Benzene) = -1972.05341083
BS2 (def2-tzvp) = -24395.8954352
Low Freq. = 5.2097cm-1, 7.5107cm-1

Rh	1.214242000	-0.065347000	1.313954000
Ge	1.865567000	0.072636000	-1.371241000
Ge	-1.338191000	2.037042000	-0.488684000
Ge	-1.366112000	-1.940698000	-0.650030000
Ge	1.137202000	2.102262000	0.136576000
Ge	-1.270203000	-0.030102000	1.235908000
Ge	1.117953000	-2.098626000	-0.069586000
Ge	-2.248942000	0.119034000	-2.089821000
Ge	0.017446000	-1.199641000	-2.923680000
Ge	0.030394000	1.493429000	-2.812140000
P	1.362923000	-0.252054000	3.639036000
Si	4.226191000	0.075044000	-2.149455000
Si	-2.863817000	-3.929236000	-0.690886000
Si	-2.736662000	4.090633000	-0.320173000
Si	-2.141328000	-5.422566000	-2.428000000
Si	-5.130468000	-3.207013000	-1.008119000
Si	-2.623879000	-4.971258000	1.459010000
Si	-4.769170000	3.865893000	-1.577928000
Si	4.835467000	2.311721000	-2.783979000
Si	5.601120000	-0.669938000	-0.330025000
Si	4.399130000	-1.434411000	-4.011985000
Si	-3.195360000	4.353121000	2.022006000

Si	-1.410324000	5.924495000	-1.126856000
C	-0.153828000	-0.080043000	4.720043000
C	2.067325000	-1.886288000	4.222604000
C	-5.948366000	5.313034000	-1.139673000
H	-6.242449000	5.281920000	-0.076956000
H	-6.870063000	5.244805000	-1.745314000
H	-5.486810000	6.294999000	-1.334898000
C	-3.531860000	-3.949225000	2.800905000
H	-3.194009000	-2.898717000	2.792555000
H	-3.333695000	-4.371227000	3.802856000
H	-4.623318000	-3.953942000	2.642160000
C	5.403106000	-2.552913000	-0.049903000
H	4.342754000	-2.822886000	0.095747000
H	5.968177000	-2.865748000	0.846860000
H	5.785206000	-3.131432000	-0.907798000
C	-2.352879000	-0.932172000	5.361214000
H	-3.142431000	-1.682235000	5.248063000
C	-1.188949000	-1.032988000	4.585756000
H	-1.077913000	-1.865021000	3.881853000
C	-0.310825000	0.978235000	5.635896000
H	0.483366000	1.720798000	5.755958000
C	-2.003535000	-4.529757000	-4.110249000
H	-2.963145000	-4.077965000	-4.411071000
H	-1.700251000	-5.244582000	-4.896246000
H	-1.249860000	-3.724474000	-4.070891000
C	-5.430432000	-2.685123000	-2.822352000
H	-4.745163000	-1.872579000	-3.117835000
H	-6.465669000	-2.320877000	-2.949396000
H	-5.281362000	-3.529294000	-3.516419000
C	1.736676000	-2.440844000	5.477791000
H	1.013906000	-1.936489000	6.126448000
C	3.556826000	3.079121000	-3.977196000
H	3.471999000	2.496624000	-4.909431000
H	3.854909000	4.109140000	-4.243613000
H	2.554460000	3.119780000	-3.517939000
C	-4.411507000	3.916169000	-3.455480000
H	-3.977209000	4.882536000	-3.761784000
H	-5.346327000	3.769576000	-4.025724000
H	-3.708295000	3.117836000	-3.746271000
C	2.538424000	0.996184000	4.396346000
C	2.325095000	-3.645963000	5.896389000
H	2.056728000	-4.068577000	6.870524000
C	-5.664603000	2.227779000	-1.176068000
H	-5.044050000	1.363445000	-1.467778000
H	-6.616135000	2.168785000	-1.734499000
H	-5.891509000	2.137096000	-0.101260000
C	4.242949000	2.942841000	5.525469000
H	4.908197000	3.695638000	5.961304000
C	-2.505943000	0.130303000	6.268795000
H	-3.418002000	0.212692000	6.869073000
C	2.992981000	-2.558229000	3.397591000
H	3.238781000	-2.143154000	2.414260000
C	-6.331770000	-4.640196000	-0.583832000
H	-6.133247000	-5.535206000	-1.196503000
H	-7.374945000	-4.327044000	-0.769936000
H	-6.253384000	-4.931575000	0.477168000
C	-1.485214000	1.083125000	6.402303000
H	-1.595092000	1.912337000	7.109226000
C	3.299148000	0.697477000	5.546776000
H	3.234336000	-0.293910000	6.005561000
C	-5.531190000	-1.729758000	0.134885000

H	-5.408746000	-1.995271000	1.197879000
H	-6.573880000	-1.398540000	-0.018029000
H	-4.868131000	-0.874925000	-0.079418000
C	0.048458000	6.271267000	0.061853000
H	-0.305887000	6.594149000	1.055212000
H	0.691817000	7.071687000	-0.345528000
H	0.671200000	5.369964000	0.196917000
C	3.248175000	-4.306859000	5.070383000
H	3.701904000	-5.248390000	5.397182000
C	-0.704313000	5.562951000	-2.864402000
H	-0.068435000	4.661430000	-2.857428000
H	-0.091495000	6.413688000	-3.212808000
H	-1.507939000	5.397517000	-3.600889000
C	-0.786760000	-5.120904000	1.966277000
H	-0.210682000	-5.707268000	1.231319000
H	-0.699699000	-5.621691000	2.947316000
H	-0.312168000	-4.128078000	2.045946000
C	-3.406611000	-6.851041000	-2.613747000
H	-3.552121000	-7.398779000	-1.668194000
H	-3.052654000	-7.571367000	-3.373230000
H	-4.389743000	-6.474058000	-2.942429000
C	-3.836237000	6.124153000	2.380566000
H	-4.098121000	6.223963000	3.449386000
H	-4.735401000	6.361988000	1.788196000
H	-3.068896000	6.882549000	2.150861000
C	-0.436838000	-6.170984000	-1.988494000
H	0.312554000	-5.379822000	-1.817874000
H	-0.074568000	-6.810422000	-2.813295000
H	-0.490480000	-6.791885000	-1.078411000
C	3.607957000	-3.126681000	-3.611684000
H	4.076781000	-3.597304000	-2.731903000
H	3.722310000	-3.812331000	-4.470474000
H	2.529709000	-3.015447000	-3.405350000
C	6.532877000	2.288909000	-3.675408000
H	7.320346000	1.831176000	-3.054413000
H	6.845896000	3.321343000	-3.914276000
H	6.478697000	1.728045000	-4.623595000
C	-3.378334000	-6.732990000	1.437071000
H	-4.434502000	-6.721457000	1.120848000
H	-3.329900000	-7.181025000	2.445890000
H	-2.825650000	-7.394662000	0.749122000
C	2.644670000	2.276895000	3.816013000
H	2.076390000	2.505083000	2.906598000
C	3.487847000	3.246540000	4.381379000
H	3.560448000	4.236359000	3.918540000
C	3.581495000	-3.759675000	3.820588000
H	4.294237000	-4.271971000	3.166052000
C	7.448357000	-0.315702000	-0.700496000
H	7.775987000	-0.787045000	-1.641932000
H	8.079499000	-0.712118000	0.115245000
H	7.641646000	0.767386000	-0.777444000
C	4.976779000	3.431624000	-1.239719000
H	4.033002000	3.430851000	-0.667406000
H	5.195788000	4.472100000	-1.539837000
H	5.783669000	3.095517000	-0.567006000
C	4.148123000	1.666701000	6.104387000
H	4.738391000	1.420613000	6.993521000
C	-2.486300000	7.507467000	-1.240343000
H	-3.290432000	7.390941000	-1.986216000
H	-1.863413000	8.365970000	-1.549966000
H	-2.953583000	7.758177000	-0.273808000

C	-4.520416000	3.100733000	2.598912000
H	-4.205027000	2.065582000	2.385469000
H	-5.488726000	3.274473000	2.100345000
H	-4.680017000	3.189140000	3.688487000
C	5.124476000	0.242042000	1.277932000
H	5.311802000	1.325690000	1.197639000
H	5.701219000	-0.146036000	2.136660000
H	4.046912000	0.111315000	1.494573000
C	3.539890000	-0.708616000	-5.557782000
H	2.476378000	-0.495822000	-5.356502000
H	3.590343000	-1.427343000	-6.395323000
H	4.019318000	0.228442000	-5.887217000
C	-1.611104000	4.069136000	3.053187000
H	-1.824876000	4.206043000	4.128298000
H	-0.811027000	4.772388000	2.768488000
H	-1.232258000	3.043064000	2.909245000
C	6.249849000	-1.723932000	-4.420883000
H	6.776000000	-0.780505000	-4.641103000
H	6.341252000	-2.378021000	-5.306719000
H	6.773422000	-2.217082000	-3.584579000

1b_iso_ts
SCF = -1972.04864464
H(0 K) = -1970.786336
H(298 K) = -1970.664709
G(298 K) = -1970.963410
SCF+D3 = -1972.35143201
PCM SCF (Benzene) = -1972.05297235
BS2 (def2-tzvp) = -24395.8949558
Low Freq. = -14.9007cm-1, 10.0566cm-1

Rh	-0.876351000	0.097610000	1.407162000
Ge	-1.854793000	0.431844000	-1.322834000
Ge	0.931195000	-2.123957000	-0.720968000
Ge	1.658697000	1.767966000	-0.507556000
Ge	-1.520745000	-1.805606000	0.006875000
Ge	1.534109000	-0.315788000	1.149826000
Ge	-0.776193000	2.250797000	0.207117000
Ge	2.180871000	-0.258851000	-2.180915000
Ge	0.180383000	1.519143000	-2.790900000
Ge	-0.311261000	-1.123917000	-2.949987000
P	-1.169882000	-0.028897000	3.707506000
Si	-4.205191000	0.914827000	-2.001233000
Si	3.465755000	3.487007000	-0.397117000
Si	1.910608000	-4.417491000	-0.790338000
Si	3.399272000	4.817382000	-2.397615000
Si	5.573869000	2.353535000	-0.185635000
Si	3.028935000	4.826187000	1.548259000
Si	4.133582000	-4.327851000	-1.688848000
Si	-5.389325000	-1.171928000	-2.137077000
Si	-5.222230000	2.374277000	-0.386303000
Si	-4.157478000	1.976941000	-4.160025000
Si	1.942211000	-5.175606000	1.487371000
Si	0.467179000	-5.808512000	-2.113474000
C	-0.251747000	-1.322025000	4.704157000
C	-0.799731000	1.539242000	4.659070000
C	5.021367000	-5.996503000	-1.365603000
H	5.136361000	-6.188209000	-0.285423000
H	6.031221000	-5.981156000	-1.813789000
H	4.469983000	-6.845257000	-1.803132000
C	3.263531000	3.780763000	3.132541000

H	2.609829000	2.892634000	3.116315000
H	3.006790000	4.374433000	4.027867000
H	4.305821000	3.436639000	3.240207000
C	-4.539265000	4.153275000	-0.551413000
H	-3.441983000	4.167613000	-0.435125000
H	-4.973385000	4.800010000	0.232342000
H	-4.786200000	4.596315000	-1.530635000
C	1.885654000	-2.269166000	5.413713000
H	2.979310000	-2.269426000	5.364504000
C	1.158780000	-1.343804000	4.651023000
H	1.691571000	-0.635736000	4.005489000
C	-0.920833000	-2.252825000	5.524870000
H	-2.012879000	-2.246932000	5.583050000
C	3.443373000	3.733275000	-3.969698000
H	4.334482000	3.084921000	-3.995918000
H	3.458550000	4.374037000	-4.869716000
H	2.552409000	3.085146000	-4.028428000
C	6.095370000	1.580040000	-1.854429000
H	5.321306000	0.890973000	-2.233088000
H	7.032885000	1.008184000	-1.733908000
H	6.268201000	2.353804000	-2.621139000
C	-0.271245000	1.514198000	5.967084000
H	-0.029017000	0.559124000	6.442463000
C	-4.384422000	-2.480390000	-3.100713000
H	-4.178645000	-2.153831000	-4.133430000
H	-4.942139000	-3.432988000	-3.146947000
H	-3.415355000	-2.674255000	-2.610571000
C	4.095149000	-4.014149000	-3.574438000
H	3.603322000	-4.840116000	-4.115041000
H	5.123610000	-3.918375000	-3.966407000
H	3.554486000	-3.081668000	-3.808626000
C	-2.952625000	-0.372098000	4.167215000
C	-0.053970000	2.714155000	6.665067000
H	0.361287000	2.681721000	7.677969000
C	5.147754000	-2.936195000	-0.861729000
H	4.689535000	-1.951861000	-1.057766000
H	6.175801000	-2.920658000	-1.265948000
H	5.210186000	-3.071246000	0.230407000
C	-5.622561000	-0.998374000	4.836696000
H	-6.659782000	-1.237159000	5.094320000
C	1.212433000	-3.197220000	6.227144000
H	1.779940000	-3.923937000	6.817884000
C	-1.108422000	2.781180000	4.066035000
H	-1.505343000	2.806477000	3.044349000
C	6.933666000	3.594036000	0.352429000
H	7.009650000	4.447559000	-0.341443000
H	7.915920000	3.088519000	0.376662000
H	6.737229000	3.993014000	1.361771000
C	-0.189644000	-3.188670000	6.276925000
H	-0.722992000	-3.908263000	6.907117000
C	-3.577606000	0.254074000	5.266022000
H	-3.027240000	0.984197000	5.867147000
C	5.484039000	0.970238000	1.129471000
H	5.222404000	1.374053000	2.121704000
H	6.459732000	0.458860000	1.213450000
H	4.723774000	0.216711000	0.862785000
C	-1.217684000	-6.033397000	-1.235636000
H	-1.105832000	-6.563979000	-0.275118000
H	-1.906153000	-6.620605000	-1.869348000
H	-1.691167000	-5.057663000	-1.031687000
C	-0.367432000	3.946136000	6.069866000

H	-0.195540000	4.879710000	6.615820000
C	0.160179000	-5.065247000	-3.845745000
H	-0.320716000	-4.074568000	-3.775617000
H	-0.501658000	-5.727449000	-4.432431000
H	1.101761000	-4.940903000	-4.405763000
C	1.245633000	5.516251000	1.542031000
H	1.055555000	6.147818000	0.658657000
H	1.074215000	6.129218000	2.445226000
H	0.505484000	4.697857000	1.536275000
C	4.924083000	5.979382000	-2.436431000
H	4.977809000	6.619079000	-1.540231000
H	4.873919000	6.638166000	-3.322100000
H	5.863380000	5.404497000	-2.499203000
C	2.179239000	-7.076816000	1.555473000
H	2.257434000	-7.411634000	2.605592000
H	3.094509000	-7.395263000	1.029483000
H	1.324355000	-7.603181000	1.098645000
C	1.814040000	5.885764000	-2.442824000
H	0.910778000	5.257708000	-2.365051000
H	1.757974000	6.445131000	-3.393817000
H	1.793892000	6.618025000	-1.618433000
C	-2.996002000	3.493042000	-4.191193000
H	-3.283964000	4.244740000	-3.438100000
H	-3.031891000	3.972718000	-5.185942000
H	-1.951357000	3.196827000	-3.993381000
C	-7.061703000	-0.924825000	-3.041333000
H	-7.680996000	-0.150208000	-2.559753000
H	-7.635084000	-1.869380000	-3.036497000
H	-6.908265000	-0.632198000	-4.093579000
C	4.228511000	6.320207000	1.617410000
H	5.284142000	6.003905000	1.594313000
H	4.065019000	6.891074000	2.549286000
H	4.062032000	7.006994000	0.770480000
C	-3.679909000	-1.310740000	3.405149000
H	-3.205347000	-1.780632000	2.535501000
C	-5.004699000	-1.627481000	3.742991000
H	-5.556462000	-2.356414000	3.140476000
C	-0.897939000	3.976658000	4.769361000
H	-1.139712000	4.932781000	4.293918000
C	-7.117819000	2.429224000	-0.670159000
H	-7.372120000	2.752895000	-1.693016000
H	-7.583204000	3.140383000	0.036032000
H	-7.578081000	1.440981000	-0.502368000
C	-5.751624000	-1.855445000	-0.386448000
H	-4.812766000	-1.996188000	0.175868000
H	-6.261469000	-2.833255000	-0.455326000
H	-6.397651000	-1.172303000	0.189966000
C	-4.907641000	-0.056761000	5.593929000
H	-5.383980000	0.440179000	6.445805000
C	1.268635000	-7.534911000	-2.345115000
H	2.210535000	-7.464680000	-2.914745000
H	0.584565000	-8.195018000	-2.908367000
H	1.490896000	-8.019799000	-1.380461000
C	3.383999000	-4.357999000	2.442980000
H	3.322431000	-3.258269000	2.382658000
H	4.363772000	-4.670057000	2.043930000
H	3.343756000	-4.643446000	3.509250000
C	-4.899316000	1.767283000	1.393315000
H	-5.349039000	0.778022000	1.577241000
H	-5.325877000	2.478719000	2.123424000
H	-3.816242000	1.680472000	1.586364000

C	-3.589918000	0.744056000	-5.506616000
H	-2.592645000	0.333449000	-5.274640000
H	-3.530382000	1.250033000	-6.486906000
H	-4.291822000	-0.101027000	-5.604894000
C	0.303720000	-4.744659000	2.369695000
H	0.329183000	-5.091495000	3.417969000
H	-0.559561000	-5.215457000	1.870236000
H	0.139061000	-3.654135000	2.379600000
C	-5.923143000	2.585944000	-4.594785000
H	-6.662933000	1.769328000	-4.568615000
H	-5.928631000	3.016746000	-5.612347000
H	-6.259102000	3.371625000	-3.897232000

1b_C3v
SCF = -1972.05032790
H(0 K) = -1970.788295
H(298 K) = -1970.665623
G(298 K) = -1970.968718
SCF+D3 = -1972.35586438
PCM SCF (Benzene) = -1972.05444079
BS2 (def2-tzvp) = -24395.8957339
Low Freq. = 6.6333cm-1, 8.7855cm-1

Rh	-0.066284005	0.017553001	1.514104107
Ge	1.187231087	-1.864717134	-0.877689062
Ge	1.076411079	1.966175140	-0.852782059
Ge	-2.182813156	-0.018258001	-0.937131066
Ge	2.200895159	0.082908006	0.578190043
Ge	-1.187764085	1.941739142	0.483091035
Ge	-1.063747079	-1.953504139	0.446898032
Ge	-0.733696053	1.377058100	-2.774900198
Ge	-0.697853053	-1.336307097	-2.777602198
Ge	1.640884117	0.061465004	-2.715260197
P	-0.120060009	-0.055435004	3.809134273
Si	2.523626181	-3.971281284	-1.071980077
Si	-4.669298334	-0.146251010	-1.190847088
Si	2.265146162	4.159664299	-1.018052073
Si	-5.329611362	-2.437709175	-0.900019066
Si	-5.267155381	0.601544042	-3.397943246
Si	-5.679992425	1.261222090	0.472209034
Si	0.982850070	5.589329386	-2.461416178
Si	4.822969349	-3.402774243	-0.673506047
Si	1.741305126	-5.539281390	0.570501039
Si	2.260314165	-4.867167349	-3.288568236
Si	2.365796172	5.085956365	1.194726084
Si	4.480070323	3.791582273	-1.872392136
C	0.233096017	1.559963110	4.689265340
C	-1.749737126	-0.563186042	4.583436330
C	1.608678114	7.393741518	-2.289262166
H	1.455470103	7.777114560	-1.266446090
H	1.056041078	8.053569561	-2.982125215
H	2.682401195	7.478606520	-2.525383182
C	-5.477129416	3.106671224	0.011621001
H	-4.412956318	3.368684242	-0.113715008
H	-5.892359404	3.745538270	0.811782057
H	-6.003342426	3.354495243	-0.925281068
C	0.059932004	-6.289674467	0.051166004
H	-0.693708050	-5.500109414	-0.108222008
H	-0.315636023	-6.962531488	0.843036059
H	0.143156010	-6.876529496	-0.878816061
C	-0.268975019	3.937007283	4.939855358

H	-0.811256060	4.826515349	4.603472330
C	-0.450358032	2.719930197	4.266872308
H	-1.122898083	2.666355191	3.402645245
C	1.115846082	1.644279117	5.785786440
H	1.649268121	0.753414055	6.129622427
C	-4.221039303	-3.616778258	-1.915351137
H	-4.266996305	-3.390457246	-2.993306217
H	-4.543156326	-4.663670338	-1.770622127
H	-3.166902226	-3.536608255	-1.599880114
C	-4.700401341	-0.666461047	-4.712046341
H	-3.611924258	-0.837758061	-4.657488336
H	-4.936717353	-0.296920021	-5.725996396
H	-5.204889375	-1.638462120	-4.580784332
C	-2.250939160	0.058006004	5.747220425
H	-1.703709125	0.887022066	6.205359439
C	5.337234372	-1.852421135	-1.664496122
H	5.210859373	-2.003447147	-2.749208198
H	6.397956462	-1.610070115	-1.472867107
H	4.728812338	-0.979383070	-1.373510099
C	1.161303085	5.060193365	-4.290003307
H	2.202249159	5.155382372	-4.641528334
H	0.527410038	5.696254412	-4.933578353
H	0.846189061	4.012806288	-4.432946316
C	1.087554081	-1.227773087	4.632239331
C	-3.451154246	-0.386770028	6.326565477
H	-3.831167277	0.107484008	7.227038514
C	-0.869925063	5.547612386	-1.997140142
H	-1.279833095	4.530889326	-2.121580152
H	-1.443140104	6.228674459	-2.651484189
H	-1.035586075	5.855727427	-0.951780068
C	2.992142218	-2.895611207	5.884679402
H	3.729144269	-3.545598256	6.367911471
C	0.614900042	4.015943288	6.029858439
H	0.763166056	4.968238357	6.549830491
C	-2.469859180	-1.633859120	4.014673289
H	-2.095928153	-2.109299149	3.099997225
C	-7.173681519	0.778767053	-3.505506253
H	-7.692465568	-0.160489012	-3.253428236
H	-7.465137531	1.062913074	-4.532817325
H	-7.541675558	1.564050115	-2.823955204
C	1.309062094	2.869608204	6.446476459
H	2.001337142	2.922490209	7.293509526
C	0.718805053	-2.033553146	5.729935433
H	-0.307813022	-2.009959144	6.106873414
C	-4.500190325	2.299833164	-3.815861275
H	-4.813198345	3.076579221	-3.098994222
H	-4.817204349	2.617457187	-4.825533350
H	-3.397656247	2.252332161	-3.803531276
C	5.528173377	2.823182203	-0.598836041
H	5.660898387	3.394849242	0.335003024
H	6.530650462	2.609314186	-1.010830074
H	5.052512363	1.861057133	-0.342869025
C	-4.157953300	-1.457938107	5.758095388
H	-5.093909365	-1.801393128	6.211096461
C	4.459818323	2.817984201	-3.515321253
H	3.989815288	1.827555133	-3.389405246
H	5.492266372	2.664434193	-3.877666278
H	3.900293283	3.356316243	-4.298036307
C	-4.891139353	0.985670071	2.188899155
H	-4.990651357	-0.058168004	2.528266182
H	-5.374540363	1.637765120	2.938674212

H	-3.814958276	1.227669087	2.167978154
C	-7.142537533	-2.679086191	-1.474809108
H	-7.833409547	-1.997130146	-0.952142068
H	-7.468651532	-3.715012268	-1.271401089
H	-7.245428534	-2.502500182	-2.558627182
C	3.629657263	6.526247495	1.256487091
H	3.613244259	6.999262528	2.255018164
H	3.402523244	7.304575522	0.509210037
H	4.656279335	6.167540464	1.073000076
C	-5.214476374	-2.944057214	0.942368069
H	-4.186967300	-2.800857199	1.317997093
H	-5.479054404	-4.009922288	1.063395075
H	-5.896757449	-2.348167166	1.571416114
C	0.422408030	-5.058547365	-3.770225272
H	-0.117741009	-5.712659436	-3.066249221
H	0.341467025	-5.500317381	-4.779722343
H	-0.089133007	-4.080713292	-3.783546272
C	5.965397453	-4.850873352	-1.195646085
H	5.693177405	-5.791458426	-0.689025048
H	7.014099494	-4.616962334	-0.938028069
H	5.918675442	-5.024779363	-2.283756167
C	-7.553290523	0.873195061	0.601101042
H	-8.065614588	0.996477072	-0.367420027
H	-8.030894580	1.556301113	1.326540094
H	-7.727678557	-0.159218011	0.948127068
C	2.418518175	-1.270344089	4.167191301
H	2.707759194	-0.661437048	3.302338237
C	3.366839245	-2.092511149	4.794250344
H	4.395335319	-2.113845152	4.419387319
C	-3.662379261	-2.083270148	4.601706333
H	-4.209607302	-2.913982209	4.144278296
C	3.009330217	-6.965963520	0.752449053
H	3.197886231	-7.472134524	-0.208769015
H	2.629990189	-7.720799570	1.464677105
H	3.975420286	-6.597807500	1.136965080
C	5.113009367	-3.052130221	1.185971084
H	4.442980321	-2.249383162	1.538283110
H	6.155617465	-2.728695198	1.356506100
H	4.928151354	-3.948245283	1.801860127
C	1.667518121	-2.865801208	6.347519467
H	1.366423098	-3.490446250	7.195263537
C	5.329280362	5.478702395	-2.204089156
H	4.800750344	6.044799441	-2.989487214
H	6.365765446	5.314259401	-2.550036185
H	5.369100368	6.108510424	-1.300403093
C	0.657480046	5.782195390	1.703204120
H	-0.122946009	5.004911361	1.640808116
H	0.355901025	6.622544472	1.055573074
H	0.692526048	6.150215465	2.744075200
C	1.521636109	-4.710080336	2.275795166
H	2.462483179	-4.273737308	2.648433191
H	1.170087083	-5.450916409	3.016398216
H	0.775922054	-3.898807279	2.223889159
C	3.117309227	-3.741293268	-4.574530330
H	2.714186195	-2.715005197	-4.537857325
H	2.955070213	-4.134569296	-5.594101401
H	4.205440301	-3.685784263	-4.403197316
C	2.902909206	3.762367271	2.461559177
H	2.915518209	4.185498299	3.481593252
H	3.911293280	3.378528241	2.233060161
H	2.205269158	2.908278207	2.457897176

C	3.070099223	-6.604197474	-3.364818243
H	4.131543296	-6.580444453	-3.068532218
H	3.013882216	-6.997355479	-4.396012316
H	2.548802186	-7.318759536	-2.705737197

1b_eta1
SCF = -1971.96969316
H(0 K) = -1970.708947
H(298 K) = -1970.586527
G(298 K) = -1970.890014
SCF+D3 = -1972.26157542
PCM SCF (Benzene) = -1971.97495005
BS2 (def2-tzvp) = -24395.8221417
Low Freq. = 4.4724cm-1, 7.3015cm-1

C	-5.707761000	0.527695000	1.865965000
C	-4.898221000	-0.018301000	2.917375000
C	-5.324607000	-1.206762000	3.577662000
C	-6.523646000	-1.820568000	3.213440000
C	-7.317499000	-1.285662000	2.170722000
C	-6.916014000	-0.122178000	1.509604000
P	-3.201926000	0.697070000	3.041739000
C	-3.332666000	2.473561000	3.549006000
C	-4.487788000	2.967748000	4.192249000
C	-4.550759000	4.314858000	4.584656000
C	-3.463370000	5.170531000	4.345856000
C	-2.311316000	4.680653000	3.707046000
C	-2.246189000	3.339069000	3.301666000
C	-2.318157000	-0.117956000	4.451168000
C	-2.219310000	0.488327000	5.721325000
C	-1.582173000	-0.189768000	6.772202000
C	-1.040977000	-1.469100000	6.563192000
C	-1.133106000	-2.072719000	5.297922000
C	-1.764611000	-1.398192000	4.241988000
Rh	-3.439625000	-0.199633000	1.053848000
Ge	-1.233337000	0.003247000	0.081564000
Ge	0.932261000	1.331144000	0.976918000
Ge	2.858744000	-0.380368000	0.439530000
Ge	2.526379000	1.088190000	-1.708680000
Ge	-0.073202000	1.624514000	-1.604399000
Ge	0.737733000	-0.281252000	-3.289677000
Ge	-0.318871000	-1.875058000	-1.459764000
Ge	2.306687000	-1.876634000	-1.658928000
Ge	0.671702000	-1.634491000	1.084271000
Si	5.055570000	-0.483255000	1.549584000
Si	6.573488000	-1.482345000	-0.022574000
C	8.374634000	-1.230363000	0.583865000
Si	4.808007000	-1.837887000	3.515288000
C	3.910177000	-3.476402000	3.116633000
Si	5.713083000	1.760916000	2.095447000
C	6.173997000	2.723653000	0.509911000
C	3.803934000	-0.907304000	4.850913000
C	6.537953000	-2.250922000	4.230488000
Si	-1.673220000	-3.892559000	-1.867641000
Si	-0.969199000	-4.892757000	-3.934407000
C	0.703731000	-5.783211000	-3.680395000
Si	-1.540263000	-5.456179000	-0.053990000
C	-2.344731000	-7.127818000	-0.538442000
Si	-3.893661000	-3.014297000	-2.073189000
C	-5.204971000	-4.404133000	-1.921617000
C	-2.463602000	-4.768769000	1.474759000

C	0.282265000	-5.775192000	0.417992000
Si	-0.518286000	3.999224000	-2.203368000
Si	0.716008000	5.557731000	-0.858974000
C	0.573786000	7.325908000	-1.588942000
Si	0.124247000	4.231269000	-4.512595000
C	2.028378000	4.267522000	-4.684990000
Si	-2.890937000	4.314634000	-2.005488000
C	-3.549275000	3.719120000	-0.314497000
C	-0.554351000	2.809849000	-5.593444000
C	-0.574589000	5.877953000	-5.206058000
C	-4.318179000	-1.746402000	-0.671972000
C	-4.150720000	-2.110597000	-3.737499000
C	-2.269265000	-6.177897000	-4.512721000
C	-0.763122000	-3.586785000	-5.312174000
C	-3.333868000	6.171587000	-2.189578000
C	-3.808864000	3.336549000	-3.370999000
C	2.566586000	5.093606000	-0.779174000
C	0.038112000	5.593968000	0.930228000
C	7.242167000	1.719303000	3.251410000
C	4.309546000	2.694245000	2.994884000
C	6.241061000	-3.358401000	-0.181555000
C	6.398229000	-0.685379000	-1.749417000
H	-3.392151000	-1.322552000	-3.878049000
H	-5.149038000	-1.638831000	-3.773191000
H	-4.076564000	-2.809723000	-4.587488000
H	-5.041444000	-5.187577000	-2.680625000
H	-6.221546000	-3.996660000	-2.066638000
H	-5.169668000	-4.883470000	-0.928832000
H	-3.836844000	-2.103012000	0.281317000
H	-5.408949000	-1.690005000	-0.524940000
H	-3.935426000	-0.742218000	-0.991631000
H	-1.711073000	-3.065620000	-5.524625000
H	-0.422359000	-4.070885000	-6.245072000
H	-0.018290000	-2.823483000	-5.029992000
H	1.470409000	-5.083201000	-3.307598000
H	1.062417000	-6.204420000	-4.636618000
H	0.617573000	-6.611632000	-2.957323000
H	-2.446808000	-6.953873000	-3.749901000
H	-1.920867000	-6.680060000	-5.433210000
H	-3.236280000	-5.697774000	-4.739543000
H	0.848225000	-6.205410000	-0.424754000
H	0.344444000	-6.478318000	1.267813000
H	0.778528000	-4.834307000	0.710589000
H	-3.395794000	-7.002544000	-0.847462000
H	-2.324675000	-7.821578000	0.321396000
H	-1.801416000	-7.608748000	-1.369116000
H	-2.070971000	-3.775060000	1.749594000
H	-2.337075000	-5.443349000	2.340632000
H	-3.544557000	-4.664682000	1.279903000
H	-3.579640000	3.726240000	-4.376856000
H	-4.901426000	3.408443000	-3.221615000
H	-3.530276000	2.269669000	-3.347457000
H	-3.362111000	2.639242000	-0.165099000
H	-4.640358000	3.892153000	-0.260724000
H	-3.078297000	4.259819000	0.523023000
H	-2.905335000	6.767706000	-1.366299000
H	-4.430548000	6.304841000	-2.164743000
H	-2.962824000	6.590355000	-3.139524000
H	-1.019948000	5.903252000	0.958170000
H	0.621773000	6.300970000	1.547150000
H	0.118333000	4.591883000	1.385413000

H	1.048067000	7.386802000	-2.582847000
H	1.085333000	8.049487000	-0.928742000
H	-0.476079000	7.645739000	-1.694127000
H	2.696970000	4.083021000	-0.356479000
H	3.113888000	5.809907000	-0.140385000
H	3.030871000	5.100586000	-1.778868000
H	-1.677567000	5.873251000	-5.215895000
H	-0.230790000	6.021273000	-6.246416000
H	-0.244077000	6.749437000	-4.617346000
H	2.463697000	5.150399000	-4.187420000
H	2.313620000	4.305563000	-5.751656000
H	2.485579000	3.366713000	-4.241485000
H	-1.654982000	2.759082000	-5.558426000
H	-0.161225000	1.834085000	-5.260310000
H	-0.251254000	2.960784000	-6.645222000
H	3.405199000	2.749335000	2.365187000
H	4.627399000	3.725348000	3.232420000
H	4.030853000	2.196098000	3.938201000
H	7.041715000	2.271796000	0.000712000
H	6.433898000	3.768403000	0.757771000
H	5.332298000	2.741870000	-0.202729000
H	7.003195000	1.242997000	4.217218000
H	7.585648000	2.748450000	3.460586000
H	8.083016000	1.167419000	2.799678000
H	5.384436000	-0.845486000	-2.154537000
H	7.121130000	-1.136517000	-2.452652000
H	6.581739000	0.401196000	-1.719450000
H	8.643314000	-0.160912000	0.611228000
H	9.078511000	-1.735817000	-0.101613000
H	8.529385000	-1.645312000	1.593758000
H	6.418664000	-3.886344000	0.770404000
H	6.908751000	-3.799868000	-0.943028000
H	5.199787000	-3.548603000	-0.491943000
H	7.131955000	-2.852145000	3.521748000
H	6.434788000	-2.835223000	5.162561000
H	7.111810000	-1.338900000	4.464386000
H	2.896095000	-3.280050000	2.728509000
H	3.816474000	-4.092964000	4.028657000
H	4.452505000	-4.065544000	2.359113000
H	4.319946000	0.010502000	5.179098000
H	3.660342000	-1.549558000	5.738382000
H	2.808253000	-0.622230000	4.472306000
H	-5.332328000	2.299319000	4.388371000
H	-1.353416000	2.963336000	2.787123000
H	-5.450526000	4.693037000	5.081407000
H	-1.463464000	5.343998000	3.509748000
H	-3.514013000	6.219884000	4.654591000
H	-4.703524000	-1.630538000	4.373265000
H	-5.480098000	1.513668000	1.446346000
H	-6.850856000	-2.724392000	3.738145000
H	-7.539805000	0.308014000	0.718746000
H	-8.256018000	-1.777486000	1.894574000
H	-2.636900000	1.485984000	5.888258000
H	-1.822674000	-1.846093000	3.243272000
H	-1.506535000	0.285439000	7.755854000
H	-0.701841000	-3.064098000	5.125961000
H	-0.540173000	-1.992079000	7.384702000

1b_H2_eta1
SCF = -1973.18143031
H(0 K) = -1971.904348

H(298 K) = -1971.781018
G(298 K) = -1972.086715
SCF+D3 = -1973.47283146
PCM SCF (Benzene) = -1973.18699251
BS2 (def2-tzvp) = -24397.0293965
Low Freq. = 7.4282cm-1, 9.7131cm-1

Ge	0.656817000	-0.012226000	-0.165290000
Ge	-0.485472000	-1.790930000	1.321493000
Ge	-0.469308000	1.780236000	1.317410000
Ge	-1.267865000	1.477918000	-1.290132000
Ge	-1.280272000	-1.488680000	-1.284984000
Ge	-1.420796000	0.000859000	3.064910000
Ge	-3.076191000	1.479682000	1.418486000
Ge	-3.362398000	0.003194000	-0.743912000
Ge	-3.087583000	-1.468172000	1.425136000
Si	0.609978000	-3.949476000	1.799879000
Si	0.629384000	3.938669000	1.792445000
Si	-5.475361000	0.003732000	-2.011279000
Si	2.863621000	-3.337790000	2.346412000
Si	-0.487934000	-4.921861000	3.701981000
Si	0.558721000	-5.415548000	-0.098113000
Si	2.885368000	3.332916000	2.340073000
Si	0.535599000	5.413438000	-0.097450000
Si	-0.453521000	4.900505000	3.710182000
Si	-5.839548000	2.243015000	-2.799232000
Si	-7.196210000	-0.672240000	-0.475955000
Si	-5.236948000	-1.559484000	-3.817802000
C	2.936501000	-2.391818000	4.005899000
H	2.261274000	-1.519891000	3.991994000
H	3.962167000	-2.032010000	4.203413000
H	2.637511000	-3.038048000	4.848269000
C	4.003648000	-4.874518000	2.461307000
H	3.625591000	-5.598929000	3.202056000
H	5.023046000	-4.576500000	2.764882000
H	4.078509000	-5.392240000	1.490271000
C	3.638811000	-2.195205000	1.000473000
H	3.611799000	-2.683623000	0.013330000
H	4.684531000	-1.955117000	1.253459000
H	3.031370000	-1.252491000	0.980289000
C	-0.770330000	-3.625854000	5.076202000
H	0.179284000	-3.194660000	5.433420000
H	-1.279843000	-4.092818000	5.938274000
H	-1.400035000	-2.795066000	4.715522000
C	-2.185036000	-5.634778000	3.185705000
H	-2.814716000	-4.855623000	2.723880000
H	-2.719380000	-6.024800000	4.070488000
H	-2.078410000	-6.461638000	2.463785000
C	0.582855000	-6.339949000	4.422240000
H	0.802778000	-7.113114000	3.667514000
H	0.052704000	-6.825722000	5.261158000
H	1.543818000	-5.960218000	4.808731000
C	-1.217545000	-5.612253000	-0.769799000
H	-1.897198000	-6.025033000	-0.006319000
H	-1.223756000	-6.293205000	-1.639711000
H	-1.623674000	-4.638884000	-1.092661000
C	1.211930000	-7.150979000	0.391459000
H	2.228936000	-7.104436000	0.814996000
H	1.243805000	-7.808157000	-0.496299000
H	0.555728000	-7.629026000	1.138114000
C	1.670678000	-4.729373000	-1.497980000

H	1.370653000	-3.701974000	-1.766343000
H	1.583404000	-5.360634000	-2.400519000
H	2.731703000	-4.713966000	-1.195233000
C	2.947168000	2.386817000	4.000249000
H	2.641412000	3.032457000	4.840560000
H	3.971868000	2.028365000	4.205588000
H	2.273386000	1.513856000	3.981625000
C	3.679489000	2.183402000	1.009154000
H	3.022070000	1.279363000	0.916768000
H	4.685920000	1.875897000	1.340956000
H	3.762992000	2.691699000	0.036211000
C	4.025740000	4.868679000	2.462385000
H	4.106628000	5.386131000	1.491789000
H	5.043355000	4.570917000	2.772663000
H	3.643569000	5.592942000	3.201082000
C	1.585361000	4.722004000	-1.539942000
H	2.653075000	4.663219000	-1.268924000
H	1.491407000	5.373958000	-2.427187000
H	1.243389000	3.711011000	-1.819831000
C	1.222672000	7.139906000	0.377035000
H	0.602481000	7.617463000	1.154122000
H	1.223266000	7.803895000	-0.506223000
H	2.255942000	7.081440000	0.757627000
C	-1.262736000	5.625585000	-0.702933000
H	-1.692870000	4.651740000	-0.991372000
H	-1.293055000	6.290071000	-1.584947000
H	-1.907019000	6.061787000	0.077849000
C	0.624179000	6.312753000	4.432125000
H	1.586190000	5.929746000	4.812637000
H	0.098667000	6.795458000	5.275727000
H	0.842018000	7.089355000	3.680244000
C	-2.152879000	5.621870000	3.212881000
H	-2.049231000	6.464240000	2.508719000
H	-2.684374000	5.992568000	4.107642000
H	-2.783773000	4.852363000	2.736816000
C	-0.731437000	3.598329000	5.079494000
H	0.218079000	3.160006000	5.427982000
H	-1.368296000	2.772830000	4.719206000
H	-1.232425000	4.063877000	5.947329000
C	-4.304471000	2.912486000	-3.719006000
H	-3.417977000	2.913345000	-3.062177000
H	-4.483749000	3.949325000	-4.055798000
H	-4.063672000	2.302178000	-4.605168000
C	-6.227659000	3.402504000	-1.329058000
H	-7.177631000	3.129096000	-0.839922000
H	-6.314574000	4.447790000	-1.675909000
H	-5.429591000	3.359636000	-0.568707000
C	-7.321511000	2.275318000	-4.015049000
H	-7.109449000	1.681541000	-4.920166000
H	-7.526060000	3.313027000	-4.334639000
H	-8.240121000	1.875352000	-3.554970000
C	-7.068163000	0.288356000	1.170308000
H	-6.105335000	0.088445000	1.670651000
H	-7.878481000	-0.019690000	1.855291000
H	-7.146451000	1.376887000	1.014773000
C	-8.916340000	-0.322099000	-1.246809000
H	-9.075125000	0.758472000	-1.401013000
H	-9.713135000	-0.685751000	-0.573208000
H	-9.040398000	-0.825566000	-2.219986000
C	-7.070600000	-2.542181000	-0.097523000
H	-7.238864000	-3.153278000	-1.000106000

H	-7.829432000	-2.829750000	0.652214000
H	-6.077333000	-2.796061000	0.309822000
C	-6.935128000	-1.825091000	-4.666695000
H	-7.663481000	-2.278388000	-3.973342000
H	-6.825856000	-2.504774000	-5.530907000
H	-7.362202000	-0.876979000	-5.033366000
C	-4.605617000	-3.249860000	-3.190383000
H	-3.614691000	-3.145982000	-2.716560000
H	-4.509608000	-3.957330000	-4.033516000
H	-5.289641000	-3.692138000	-2.447821000
C	-4.000851000	-0.894897000	-5.116972000
H	-4.368168000	0.031797000	-5.588892000
H	-3.845205000	-1.642558000	-5.915299000
H	-3.021610000	-0.678619000	-4.657886000
P	5.136040000	-0.010634000	-1.874302000
C	5.843915000	1.694271000	-2.169170000
C	7.205770000	2.004844000	-1.972828000
C	4.968347000	2.704541000	-2.625806000
C	7.680373000	3.303329000	-2.225886000
H	7.897861000	1.233234000	-1.622371000
C	5.448214000	3.996748000	-2.886757000
H	3.907120000	2.475928000	-2.772039000
C	6.805075000	4.300454000	-2.683564000
H	8.739601000	3.531810000	-2.066616000
H	4.756683000	4.767988000	-3.241245000
H	7.177564000	5.311256000	-2.879816000
C	6.541909000	-0.899804000	-1.021292000
C	7.271819000	-1.940592000	-1.630337000
C	6.845135000	-0.540640000	0.312105000
C	8.287124000	-2.604750000	-0.920572000
H	7.050287000	-2.231505000	-2.661452000
C	7.868516000	-1.195423000	1.012919000
H	6.277603000	0.259954000	0.799836000
C	8.590146000	-2.233539000	0.398257000
H	8.844822000	-3.412687000	-1.405954000
H	8.097615000	-0.899535000	2.042169000
H	9.383523000	-2.751021000	0.947385000
C	5.159297000	-0.755226000	-3.587773000
C	5.992938000	-0.247515000	-4.606549000
C	4.344661000	-1.874179000	-3.860689000
C	6.005740000	-0.847617000	-5.875993000
H	6.628588000	0.621561000	-4.410697000
C	4.366448000	-2.477067000	-5.127991000
H	3.683181000	-2.265784000	-3.080638000
C	5.194388000	-1.962927000	-6.139115000
H	6.650822000	-0.438840000	-6.660912000
H	3.726052000	-3.342673000	-5.326486000
H	5.202935000	-2.426798000	-7.130989000
Rh	3.043003000	-0.029855000	-0.806942000
H	2.510359000	-1.051715000	-1.812107000
H	2.534106000	0.956154000	-1.864070000

1b_PPh3diss
SCF = -1270.48818934
H(0 K) = -1269.493521
H(298 K) = -1269.389066
G(298 K) = -1269.650971
SCF+D3 = -1270.71390127
PCM SCF (Benzene) = -1270.49277358
BS2 (def2-tzvp) = -23359.2655215
Low Freq. = 8.3849cm-1, 10.1830cm-1

Rh	1.225343000	-0.081074000	-2.236266000
Ge	2.250112000	-0.015161000	0.158913000
Ge	-1.103918000	-2.076755000	-0.124201000
Ge	-1.082607000	2.093542000	-0.216506000
Ge	1.280032000	-2.199240000	-0.983129000
Ge	-1.222371000	-0.033323000	-1.881144000
Ge	1.294383000	2.106469000	-1.111594000
Ge	-1.759785000	0.044491000	1.337122000
Ge	0.656295000	1.410383000	1.840442000
Ge	0.631878000	-1.324447000	1.920689000
Si	4.727890000	0.014693000	0.252726000
Si	-2.601087000	4.043659000	0.085999000
Si	-2.617710000	-4.036698000	0.111186000
Si	-2.305885000	4.967200000	2.284018000
Si	-4.852507000	3.283167000	-0.261972000
Si	-1.942809000	5.593520000	-1.628844000
Si	-4.705260000	-3.394648000	1.105420000
Si	5.485116000	-2.188223000	0.848195000
Si	5.486465000	0.624761000	-1.946973000
Si	5.368098000	1.648119000	1.894784000
Si	-2.922617000	-4.770845000	-2.159303000
Si	-1.452913000	-5.688368000	1.409230000
C	-5.982246000	-4.803257000	0.864665000
H	-6.189773000	-4.979156000	-0.204246000
H	-6.936684000	-4.536419000	1.353184000
H	-5.631979000	-5.752692000	1.302543000
C	-2.377557000	4.888533000	-3.353196000
H	-1.895969000	3.909304000	-3.514812000
H	-2.029023000	5.574416000	-4.146017000
H	-3.465556000	4.756265000	-3.475806000
C	5.331064000	2.506533000	-2.236714000
H	4.289491000	2.842278000	-2.094928000
H	5.634599000	2.758504000	-3.268741000
H	5.973361000	3.077513000	-1.545474000
C	-2.463312000	3.624657000	3.633324000
H	-3.432916000	3.102860000	3.581690000
H	-2.372312000	4.082135000	4.634882000
H	-1.665991000	2.868810000	3.531945000
C	-5.507152000	2.391214000	1.296985000
H	-4.856468000	1.544786000	1.574786000
H	-6.522084000	1.995748000	1.113008000
H	-5.563025000	3.076297000	2.159627000
C	4.497730000	-2.868930000	2.334161000
H	4.607256000	-2.224534000	3.222023000
H	4.853813000	-3.880461000	2.599534000
H	3.421761000	-2.935280000	2.098992000
C	-4.498156000	-3.070992000	2.977898000
H	-4.181174000	-3.979901000	3.516004000
H	-5.456715000	-2.737036000	3.413991000
H	-3.748653000	-2.283356000	3.163400000
C	-5.386150000	-1.810198000	0.285199000
H	-4.695033000	-0.964428000	0.441611000
H	-6.361307000	-1.539973000	0.728344000
H	-5.526283000	-1.938764000	-0.800423000
C	-5.992767000	4.780583000	-0.623141000
H	-5.952101000	5.529870000	0.184790000
H	-7.040364000	4.442964000	-0.719895000
H	-5.713417000	5.280944000	-1.565419000
C	-4.942595000	2.085311000	-1.747086000
H	-4.596713000	2.568205000	-2.676044000

H	-5.982451000	1.746970000	-1.903794000
H	-4.314257000	1.194190000	-1.579141000
C	0.089557000	-6.310587000	0.465427000
H	-0.187004000	-6.823975000	-0.470600000
H	0.655590000	-7.025647000	1.088902000
H	0.763945000	-5.475651000	0.209584000
C	-0.893063000	-4.977870000	3.091227000
H	-0.197982000	-4.131690000	2.953138000
H	-0.372868000	-5.755487000	3.678864000
H	-1.748343000	-4.616312000	3.685478000
C	-0.063639000	5.939327000	-1.577833000
H	0.246819000	6.371176000	-0.612201000
H	0.212499000	6.651669000	-2.375929000
H	0.515507000	5.012616000	-1.733447000
C	-3.654877000	6.293734000	2.591598000
H	-3.621810000	7.095367000	1.835292000
H	-3.509162000	6.757173000	3.583972000
H	-4.665132000	5.851456000	2.572854000
C	-3.674302000	-6.533285000	-2.185421000
H	-3.865974000	-6.846974000	-3.227314000
H	-4.629401000	-6.579716000	-1.636444000
H	-2.987519000	-7.269036000	-1.734345000
C	-0.588038000	5.790464000	2.443697000
H	0.218768000	5.069392000	2.230032000
H	-0.441401000	6.168904000	3.471167000
H	-0.479075000	6.641894000	1.751325000
C	4.464108000	3.306941000	1.616445000
H	4.668563000	3.718967000	0.614728000
H	4.792929000	4.048093000	2.366927000
H	3.372410000	3.181585000	1.716268000
C	7.337619000	-2.113201000	1.333887000
H	7.958322000	-1.696679000	0.523358000
H	7.708576000	-3.130042000	1.555846000
H	7.493470000	-1.496022000	2.234493000
C	-2.858344000	7.260971000	-1.396172000
H	-3.953296000	7.133654000	-1.400652000
H	-2.593260000	7.954004000	-2.214807000
H	-2.576520000	7.742687000	-0.444803000
C	7.326376000	0.127924000	-2.144795000
H	7.959702000	0.615591000	-1.384884000
H	7.697184000	0.431668000	-3.140101000
H	7.463153000	-0.962420000	-2.052407000
C	5.295271000	-3.391019000	-0.625839000
H	4.243338000	-3.447890000	-0.954604000
H	5.618182000	-4.406320000	-0.333453000
H	5.906898000	-3.076950000	-1.488398000
C	-2.611593000	-7.178719000	1.739411000
H	-3.484638000	-6.881510000	2.344399000
H	-2.068139000	-7.962886000	2.296657000
H	-2.984156000	-7.624670000	0.802554000
C	-4.100615000	-3.589526000	-3.094089000
H	-3.720325000	-2.554000000	-3.075622000
H	-5.112487000	-3.590467000	-2.655478000
H	-4.190526000	-3.899807000	-4.150619000
C	4.465533000	-0.276478000	-3.290948000
H	4.557228000	-1.372216000	-3.216175000
H	4.778626000	0.037516000	-4.302884000
H	3.384721000	-0.020785000	-3.173423000
C	4.959684000	1.016124000	3.652011000
H	3.882303000	0.803820000	3.756567000
H	5.229584000	1.779317000	4.403782000

H	5.514568000	0.093989000	3.893035000
C	-1.256779000	-4.814086000	-3.096739000
H	-1.412590000	-5.169762000	-4.131156000
H	-0.530445000	-5.485776000	-2.610075000
H	-0.806941000	-3.807818000	-3.145598000
C	7.255446000	1.955452000	1.768804000
H	7.833136000	1.026807000	1.907625000
H	7.572783000	2.671980000	2.547680000
H	7.528934000	2.382389000	0.789172000

Rh_H2
SCF = -1271.69380361
H(0 K) = -1270.684551
H(298 K) = -1270.579024
G(298 K) = -1270.843958
SCF+D3 = -1271.92339197
PCM SCF (Benzene) = -1271.69606287
BS2 (def2-tzvp) = -23360.4713327
Low Freq. = 2.8816cm-1, 9.7659cm-1

Rh	0.796518000	0.030821000	-2.335249000
Ge	1.091205000	2.145995000	-1.141246000
Ge	-1.201912000	1.940559000	0.006702000
Si	-2.675303000	3.946077000	0.103981000
Si	-3.457920000	4.241572000	-2.149909000
C	-4.240881000	5.979760000	-2.342780000
Ge	2.194999000	0.113334000	0.109263000
Si	4.677938000	0.193970000	0.202425000
Si	5.389051000	-1.400503000	1.853944000
C	7.286717000	-1.636104000	1.724882000
Ge	1.302888000	-2.033285000	-1.117613000
Ge	-1.015627000	-2.042897000	-0.007720000
Si	-2.324542000	-4.157541000	0.102954000
Si	-2.587292000	-4.849116000	-2.185758000
C	-3.202340000	-6.662792000	-2.248710000
Ge	-1.634430000	-0.075007000	-1.674855000
Ge	0.635341000	1.390155000	1.957436000
Ge	0.765381000	-1.315390000	1.952357000
Ge	-1.681331000	-0.084475000	1.691729000
Si	5.514656000	-0.386862000	-1.977032000
C	4.559575000	0.537438000	-3.347408000
Si	5.339634000	2.426094000	0.799434000
C	5.059983000	3.620900000	-0.667180000
Si	-1.315132000	5.802369000	0.795215000
C	-0.060568000	6.257764000	-0.574459000
Si	-4.493104000	3.569488000	1.626540000
C	-5.328035000	1.882684000	1.306420000
Si	-1.047674000	-5.753143000	1.364509000
C	-0.551588000	-5.050450000	3.069743000
Si	-4.459312000	-3.711441000	1.107863000
C	-5.264412000	-2.163051000	0.332980000
C	-0.354529000	5.401155000	2.396603000
C	-2.429483000	7.325458000	1.129315000
C	-3.873143000	3.607213000	3.434714000
C	-5.805274000	4.947872000	1.398947000
C	-4.775534000	2.925701000	-2.582451000
C	-2.018272000	4.085951000	-3.398013000
C	-2.099794000	-7.329595000	1.648390000
C	0.540242000	-6.238490000	0.416111000
C	5.364663000	-2.266198000	-2.291831000
C	7.365047000	0.100289000	-2.083505000

C	4.357194000	3.065056000	2.307736000
C	7.202467000	2.442433000	1.250114000
C	4.968149000	-0.783177000	3.613882000
C	4.553437000	-3.097639000	1.588112000
C	-5.623462000	-5.206451000	0.819924000
C	-4.278294000	-3.430761000	2.990083000
C	-3.864808000	-3.741139000	-3.078739000
C	-0.934860000	-4.745127000	-3.141013000
H	-2.990741000	-7.110888000	2.260703000
H	-1.502935000	-8.089613000	2.184003000
H	-2.441177000	-7.773681000	0.698904000
H	-4.371316000	1.906676000	-2.456797000
H	-5.095242000	3.037710000	-3.634041000
H	-5.670665000	3.020574000	-1.945383000
H	3.274650000	3.095976000	2.096659000
H	4.684057000	4.087220000	2.570277000
H	4.506544000	2.421867000	3.190651000
H	-1.036701000	5.161483000	3.228879000
H	0.261707000	6.266975000	2.698567000
H	0.316042000	4.536495000	2.254895000
H	-3.083867000	2.855007000	3.600750000
H	-4.704888000	3.384556000	4.126859000
H	-3.465611000	4.595850000	3.704507000
H	5.976906000	-2.849302000	-1.583685000
H	5.707381000	-2.506497000	-3.314386000
H	4.317182000	-2.599970000	-2.197290000
H	0.306740000	-6.746190000	-0.534680000
H	1.152709000	-6.927006000	1.025374000
H	1.154557000	-5.351307000	0.186666000
H	0.081138000	-4.152650000	2.962215000
H	0.019524000	-5.804748000	3.640232000
H	-1.435328000	-4.768980000	3.665647000
H	-5.376040000	5.952593000	1.546857000
H	-6.621040000	4.816098000	2.132400000
H	-6.251999000	4.914694000	0.391003000
H	-5.699054000	1.797071000	0.272060000
H	-6.184202000	1.745915000	1.991064000
H	-4.616847000	1.057703000	1.482775000
H	-4.870123000	-3.840899000	-2.636432000
H	-3.934951000	-4.024257000	-4.144359000
H	-3.572683000	-2.678262000	-3.027145000
H	-0.560824000	-3.707650000	-3.172504000
H	-1.076553000	-5.088357000	-4.181707000
H	-0.154782000	-5.371867000	-2.677999000
H	-1.231886000	4.835349000	-3.209106000
H	-2.391013000	4.232690000	-4.427775000
H	-1.554424000	3.086482000	-3.341628000
H	-3.032159000	7.595497000	0.246519000
H	-1.805347000	8.197833000	1.394176000
H	-3.120379000	7.140595000	1.968943000
H	-6.593885000	-5.030364000	1.317963000
H	-5.200159000	-6.140605000	1.224706000
H	-5.821083000	-5.360680000	-0.254198000
H	0.571294000	5.392620000	-0.839694000
H	0.603597000	7.070761000	-0.230543000
H	-0.568914000	6.601220000	-1.490964000
H	5.496987000	0.154617000	3.853178000
H	5.264249000	-1.538801000	4.363440000
H	3.885574000	-0.602730000	3.724548000
H	7.499581000	1.190969000	-1.990520000
H	7.780268000	-0.207752000	-3.059945000

H	7.961985000	-0.384895000	-1.293492000
H	-5.064669000	6.139230000	-1.627432000
H	-4.648993000	6.099553000	-3.362460000
H	-3.494651000	6.776282000	-2.184301000
H	7.403935000	1.833956000	2.147680000
H	7.527252000	3.476179000	1.466259000
H	7.828600000	2.055844000	0.429167000
H	3.484284000	0.278871000	-3.312515000
H	4.950806000	0.257877000	-4.342409000
H	4.643660000	1.631250000	-3.236965000
H	-2.454769000	-7.355015000	-1.826435000
H	-3.384773000	-6.963531000	-3.296074000
H	-4.142949000	-6.796635000	-1.689397000
H	-3.587421000	-2.597224000	3.201792000
H	-3.896518000	-4.330438000	3.500981000
H	-5.258520000	-3.180434000	3.433907000
H	5.684580000	3.350046000	-1.534965000
H	5.316539000	4.654658000	-0.374001000
H	4.004998000	3.609488000	-0.991101000
H	3.458025000	-3.017190000	1.694071000
H	4.915867000	-3.821011000	2.340419000
H	4.770342000	-3.505997000	0.587434000
H	-6.252764000	-1.978326000	0.790547000
H	-5.406557000	-2.278160000	-0.754009000
H	-4.641129000	-1.268708000	0.500529000
H	7.573184000	-2.069620000	0.751929000
H	7.634991000	-2.325343000	2.515100000
H	7.828204000	-0.683222000	1.844502000
H	0.846909000	0.031025000	-4.027158000
H	-0.043200000	-0.018799000	-3.803259000

1b_H2_diss_ts
SCF = -1973.21515743
H(0 K) = -1971.940920
H(298 K) = -1971.816691
G(298 K) = -1972.123549
SCF+D3 = -1973.51619702
PCM SCF (Benzene) = -1973.21959029
BS2 (def2-tzvp) = -24397.0625713
Low Freq. = -188.2239cm-1, 5.7910cm-1

C	-3.866229000	-2.351772000	6.188423000
C	-4.044395000	-3.548509000	5.476212000
C	-3.345365000	-3.754446000	4.275870000
C	-2.476340000	-2.765582000	3.788417000
C	-2.282609000	-1.564638000	4.505569000
C	-2.987813000	-1.365468000	5.710899000
P	-1.083142000	-0.296001000	3.816954000
C	-1.529404000	1.260743000	4.755348000
C	-1.111681000	1.472454000	6.088009000
C	-1.505251000	2.630504000	6.777887000
C	-2.318575000	3.586823000	6.147928000
C	-2.734810000	3.383949000	4.822564000
C	-2.340159000	2.228907000	4.128933000
Rh	-1.322412000	-0.129098000	1.486851000
Ge	-1.153387000	2.078622000	0.339800000
Ge	1.258483000	2.055471000	-0.437991000
Si	2.725314000	4.063829000	-0.463519000
Si	1.995146000	5.467720000	1.343365000
C	2.908162000	7.152710000	1.298672000
Ge	-2.000280000	0.048802000	-1.174571000

Si	-4.370495000	0.091105000	-1.964927000
Si	-4.367824000	0.341959000	-4.366870000
C	-6.139209000	0.026045000	-5.032523000
Ge	-1.113941000	-2.155665000	0.033611000
Ge	1.318650000	-1.920340000	-0.647051000
Si	2.855913000	-3.859813000	-0.924259000
Si	2.005548000	-5.632298000	0.456304000
C	2.961726000	-7.259469000	0.117241000
Ge	1.173208000	-0.038335000	1.276276000
Ge	-0.275402000	1.526440000	-2.650520000
Ge	-0.214945000	-1.192640000	-2.808849000
Ge	2.090268000	0.169524000	-2.110503000
C	0.525653000	-0.788055000	4.633320000
C	1.585309000	0.147117000	4.677125000
C	2.817862000	-0.203731000	5.247725000
C	3.015026000	-1.495279000	5.764506000
C	1.970519000	-2.430644000	5.716129000
C	0.729295000	-2.079754000	5.157360000
Si	-5.470382000	-1.977499000	-1.415090000
C	-5.261547000	-2.469954000	0.419356000
Si	-5.555669000	1.975365000	-1.040586000
C	-6.014773000	1.706374000	0.798296000
Si	2.505130000	5.179473000	-2.580280000
C	0.764291000	5.949717000	-2.758653000
Si	4.982797000	3.331643000	-0.101450000
C	5.055767000	2.062956000	1.326082000
Si	2.908123000	-4.523677000	-3.234182000
C	3.308188000	-3.055541000	-4.388244000
Si	5.026878000	-3.180879000	-0.158408000
C	4.899041000	-2.277652000	1.520473000
C	2.789642000	3.979983000	-4.038985000
C	3.809493000	6.580066000	-2.697787000
C	5.691520000	2.521723000	-1.681294000
C	6.099845000	4.818532000	0.364912000
C	2.362685000	4.621912000	3.021476000
C	0.117773000	5.816469000	1.253544000
C	4.263845000	-5.857040000	-3.483481000
C	1.222988000	-5.260105000	-3.757382000
C	-4.775032000	-3.399415000	-2.488927000
C	-7.351603000	-1.817892000	-1.751495000
C	-4.539208000	3.588023000	-1.169686000
C	-7.191845000	2.235666000	-2.006261000
C	-3.843544000	2.112735000	-4.863478000
C	-3.215560000	-0.895780000	-5.253742000
C	6.157903000	-4.711929000	0.072871000
C	5.853364000	-2.011995000	-1.425885000
C	2.209613000	-5.193848000	2.308336000
C	0.152771000	-5.968608000	0.123382000
H	5.267947000	-5.452230000	-3.272180000
H	4.257102000	-6.207162000	-4.531533000
H	4.107690000	-6.732943000	-2.832568000
H	1.890705000	3.625720000	3.067281000
H	1.964070000	5.227747000	3.854806000
H	3.446140000	4.493659000	3.182850000
H	-3.610635000	3.514849000	-0.576739000
H	-5.127647000	4.440204000	-0.784189000
H	-4.255843000	3.810088000	-2.211493000
H	3.626829000	0.533043000	5.280755000
H	1.440722000	1.158570000	4.283100000
H	-0.082187000	-2.813180000	5.138485000
H	3.784371000	3.507354000	-3.992696000

H	2.712621000	4.525180000	-4.996936000
H	2.034253000	3.175988000	-4.043435000
H	5.050975000	1.691484000	-2.024839000
H	6.700926000	2.117811000	-1.485262000
H	5.773931000	3.250790000	-2.504813000
H	-0.470802000	0.737104000	6.584491000
H	-4.977037000	-3.237312000	-3.560803000
H	-5.239466000	-4.358883000	-2.198405000
H	-3.683948000	-3.496085000	-2.358492000
H	0.981386000	-6.173904000	-3.189197000
H	1.239215000	-5.522628000	-4.830391000
H	0.409685000	-4.531715000	-3.598990000
H	-1.171163000	2.785545000	7.809293000
H	2.534791000	-2.272242000	-4.318576000
H	3.350115000	-3.401334000	-5.436875000
H	4.277740000	-2.593031000	-4.141232000
H	-4.730973000	-4.314616000	5.851366000
H	3.979509000	-1.769344000	6.204354000
H	-2.641596000	2.083328000	3.085245000
H	6.071005000	5.609267000	-0.403104000
H	7.148308000	4.485573000	0.468967000
H	5.793529000	5.266618000	1.325096000
H	2.113838000	-3.438180000	6.120205000
H	-2.858288000	-0.438037000	6.276373000
H	4.708413000	2.505090000	2.274696000
H	6.091278000	1.707265000	1.472970000
H	4.419066000	1.188355000	1.111525000
H	3.273536000	-5.120533000	2.589800000
H	1.746930000	-5.974963000	2.938309000
H	1.729952000	-4.230381000	2.549088000
H	-2.620727000	4.490830000	6.687002000
H	-0.456717000	-5.070412000	0.325283000
H	-0.209081000	-6.783502000	0.776157000
H	-0.026206000	-6.264828000	-0.923416000
H	-0.156396000	6.332899000	0.318873000
H	-0.192304000	6.453750000	2.101212000
H	-0.461529000	4.878013000	1.300305000
H	3.712687000	7.303933000	-1.871896000
H	3.686933000	7.130569000	-3.647983000
H	4.835176000	6.174920000	-2.674981000
H	7.174536000	-4.391004000	0.363460000
H	6.241903000	-5.302516000	-0.854419000
H	5.777140000	-5.377608000	0.865638000
H	-0.019954000	5.180869000	-2.657026000
H	0.654927000	6.417087000	-3.753693000
H	0.582908000	6.727465000	-1.998075000
H	-4.547339000	2.871453000	-4.482290000
H	-3.812831000	2.202690000	-5.964250000
H	-2.838090000	2.350160000	-4.475296000
H	-7.816069000	-1.077465000	-1.078430000
H	-7.846918000	-2.789472000	-1.573158000
H	-7.564706000	-1.514382000	-2.789280000
H	4.003237000	7.028264000	1.316685000
H	2.620094000	7.762802000	2.173686000
H	2.647312000	7.723713000	0.391706000
H	-1.955084000	-2.914997000	2.835052000
H	-3.483160000	-4.680535000	3.708159000
H	-3.360437000	4.129394000	4.320706000
H	-7.000043000	2.529782000	-3.051447000
H	-7.776140000	3.045083000	-1.532317000
H	-7.817501000	1.328033000	-2.016241000

H	-4.196424000	-2.602564000	0.673840000
H	-5.782320000	-3.426389000	0.607079000
H	-5.685046000	-1.711447000	1.097891000
H	-4.413236000	-2.180539000	7.121660000
H	2.792184000	-7.615620000	-0.912734000
H	2.616810000	-8.050929000	0.806857000
H	4.048027000	-7.135995000	0.258461000
H	5.211664000	-1.141600000	-1.644467000
H	6.057295000	-2.530052000	-2.377991000
H	6.815549000	-1.638464000	-1.031832000
H	-6.615797000	0.792885000	0.941028000
H	-6.608791000	2.563570000	1.163651000
H	-5.115138000	1.623313000	1.429452000
H	-2.158943000	-0.727793000	-4.986139000
H	-3.315697000	-0.774682000	-6.347694000
H	-3.463397000	-1.939940000	-5.001764000
H	5.898725000	-1.946850000	1.854934000
H	4.476749000	-2.932449000	2.300480000
H	4.249854000	-1.388767000	1.447375000
H	-6.439181000	-1.025186000	-4.885828000
H	-6.162349000	0.230795000	-6.118298000
H	-6.894763000	0.667369000	-4.551001000
H	-3.443657000	-0.031625000	1.463355000
H	-3.564697000	-0.399490000	2.130958000

1b_H2

SCF = -1973.21548071
H(0 K) = -1971.939936
H(298 K) = -1971.815753
G(298 K) = -1972.121724
SCF+D3 = -1973.51707851
PCM SCF (Benzene) = -1973.21992643
BS2 (def2-tzvp) = -24397.0624785
Low Freq. = 7.5475cm-1, 9.1755cm-1

C	-4.091939000	-2.489706000	5.998734000
C	-4.259219000	-3.666957000	5.252765000
C	-3.515502000	-3.856349000	4.076669000
C	-2.613081000	-2.870516000	3.647420000
C	-2.429392000	-1.689699000	4.400107000
C	-3.179869000	-1.506893000	5.579996000
P	-1.189179000	-0.425656000	3.781377000
C	-1.591724000	1.099650000	4.789813000
C	-1.217085000	1.208253000	6.147822000
C	-1.572702000	2.344248000	6.892280000
C	-2.303725000	3.382736000	6.291786000
C	-2.674705000	3.283872000	4.941614000
C	-2.318112000	2.150047000	4.193934000
Rh	-1.422513000	-0.172888000	1.462388000
Ge	-1.120171000	2.071186000	0.374761000
Ge	1.295556000	2.042797000	-0.370566000
Si	2.762263000	4.049371000	-0.299078000
Si	1.992517000	5.383998000	1.543085000
C	2.937450000	7.050602000	1.615013000
Ge	-1.981037000	0.098110000	-1.212384000
Si	-4.335447000	0.179049000	-2.038581000
Si	-4.308041000	0.624174000	-4.410366000
C	-6.061111000	0.316368000	-5.125900000
Ge	-1.100524000	-2.162599000	-0.076305000
Ge	1.337328000	-1.874165000	-0.693753000
Si	2.889264000	-3.799511000	-0.975594000

Si	2.027199000	-5.606303000	0.353395000
C	3.010923000	-7.215867000	0.007972000
Ge	1.091695000	-0.084351000	1.315874000
Ge	-0.234660000	1.618755000	-2.606337000
Ge	-0.187154000	-1.106424000	-2.850366000
Ge	2.130952000	0.230676000	-2.122152000
C	0.400231000	-0.996302000	4.587503000
C	1.473984000	-0.083825000	4.707703000
C	2.691994000	-0.491860000	5.271863000
C	2.860603000	-1.817007000	5.706627000
C	1.802195000	-2.729594000	5.582210000
C	0.575883000	-2.322669000	5.028678000
Si	-5.401534000	-1.942252000	-1.653224000
C	-5.212615000	-2.534125000	0.154600000
Si	-5.564337000	1.956278000	-0.972614000
C	-6.038711000	1.519870000	0.830202000
Si	2.581939000	5.228745000	-2.384929000
C	0.841943000	5.996512000	-2.580517000
Si	5.010838000	3.297944000	0.078987000
C	5.052082000	1.984599000	1.466676000
Si	2.975130000	-4.415552000	-3.298243000
C	3.389771000	-2.925354000	-4.418143000
Si	5.044836000	-3.125405000	-0.163720000
C	4.884080000	-2.268978000	1.536530000
C	2.902637000	4.073687000	-3.871835000
C	3.881461000	6.638025000	-2.432745000
C	5.741304000	2.534688000	-1.514038000
C	6.128325000	4.762001000	0.611274000
C	2.278634000	4.451135000	3.190410000
C	0.127079000	5.777072000	1.401139000
C	4.335293000	-5.742948000	-3.554821000
C	1.298268000	-5.143008000	-3.859149000
C	-4.649604000	-3.277784000	-2.796484000
C	-7.278695000	-1.802842000	-2.017936000
C	-4.575091000	3.590469000	-0.949014000
C	-7.194066000	2.269288000	-1.933884000
C	-3.828662000	2.444497000	-4.748009000
C	-3.108133000	-0.505094000	-5.375461000
C	6.186203000	-4.652196000	0.042500000
C	5.877359000	-1.915588000	-1.387807000
C	2.181646000	-5.200064000	2.217197000
C	0.187162000	-5.961126000	-0.027626000
H	5.336315000	-5.341476000	-3.323577000
H	4.341851000	-6.073507000	-4.609205000
H	4.171943000	-6.630936000	-2.922355000
H	1.783694000	3.465231000	3.170141000
H	1.862239000	5.025876000	4.036953000
H	3.352158000	4.289637000	3.384654000
H	-3.649308000	3.480088000	-0.357302000
H	-5.180287000	4.396211000	-0.495603000
H	-4.287734000	3.907337000	-1.964903000
H	3.511859000	0.227598000	5.364309000
H	1.351720000	0.954236000	4.381345000
H	-0.246663000	-3.039724000	4.951048000
H	3.899919000	3.607043000	-3.821398000
H	2.838263000	4.645709000	-4.814975000
H	2.153836000	3.264606000	-3.913062000
H	5.099739000	1.723095000	-1.897882000
H	6.743098000	2.114380000	-1.313549000
H	5.845748000	3.290730000	-2.310257000
H	-0.638972000	0.408693000	6.621744000

H	-4.826054000	-3.047475000	-3.860392000
H	-5.100588000	-4.263651000	-2.583790000
H	-3.560437000	-3.360424000	-2.642129000
H	1.053344000	-6.071535000	-3.316940000
H	1.327579000	-5.378342000	-4.938164000
H	0.480746000	-4.421393000	-3.691569000
H	-1.273232000	2.418054000	7.943138000
H	2.615230000	-2.143542000	-4.344726000
H	3.446642000	-3.251414000	-5.472389000
H	4.355390000	-2.467139000	-4.148931000
H	-4.971552000	-4.430522000	5.582304000
H	3.813349000	-2.134989000	6.142468000
H	-2.582720000	2.085118000	3.132063000
H	6.114051000	5.579293000	-0.128763000
H	7.173537000	4.419687000	0.717766000
H	5.811252000	5.177923000	1.582363000
H	1.922827000	-3.763251000	5.922562000
H	-3.060609000	-0.594848000	6.171940000
H	4.695774000	2.400202000	2.423930000
H	6.082009000	1.614269000	1.616584000
H	4.410288000	1.123424000	1.216333000
H	3.237307000	-5.116762000	2.525570000
H	1.715830000	-6.000141000	2.820571000
H	1.682068000	-4.248571000	2.464951000
H	-2.576229000	4.269520000	6.873591000
H	-0.439101000	-5.074043000	0.172031000
H	-0.178209000	-6.789441000	0.606079000
H	0.035807000	-6.245864000	-1.081911000
H	-0.100972000	6.348412000	0.486304000
H	-0.202527000	6.374977000	2.269884000
H	-0.471832000	4.850100000	1.375540000
H	3.760814000	7.339141000	-1.590394000
H	3.778539000	7.213446000	-3.370437000
H	4.908707000	6.237883000	-2.396052000
H	7.195955000	-4.329923000	0.354806000
H	6.287825000	-5.217900000	-0.898437000
H	5.801195000	-5.341404000	0.812780000
H	0.059428000	5.221760000	-2.517223000
H	0.752831000	6.489739000	-3.565055000
H	0.639690000	6.752862000	-1.803875000
H	-4.559592000	3.147187000	-4.313920000
H	-3.786545000	2.629526000	-5.836422000
H	-2.835742000	2.676155000	-4.325832000
H	-7.772213000	-1.112636000	-1.312971000
H	-7.756958000	-2.792881000	-1.907866000
H	-7.476701000	-1.442335000	-3.040453000
H	4.027672000	6.904041000	1.685130000
H	2.614303000	7.630370000	2.498369000
H	2.735664000	7.663089000	0.720212000
H	-2.057595000	-3.006082000	2.711629000
H	-3.644234000	-4.766917000	3.482323000
H	-3.234742000	4.093801000	4.462695000
H	-6.996451000	2.650163000	-2.949649000
H	-7.798232000	3.027330000	-1.403318000
H	-7.802986000	1.354763000	-2.025217000
H	-4.149232000	-2.689046000	0.406656000
H	-5.738329000	-3.496533000	0.291096000
H	-5.636206000	-1.810104000	0.870182000
H	-4.673699000	-2.330500000	6.912900000
H	2.867953000	-7.556972000	-1.031084000
H	2.660574000	-8.022604000	0.676849000

H	4.092359000	-7.082386000	0.174857000
H	5.229035000	-1.047087000	-1.594069000
H	6.101979000	-2.406303000	-2.349647000
H	6.828876000	-1.541666000	-0.969024000
H	-6.639857000	0.596889000	0.884972000
H	-6.635542000	2.339361000	1.269907000
H	-5.142254000	1.379918000	1.456472000
H	-2.060837000	-0.329811000	-5.078208000
H	-3.196194000	-0.299478000	-6.457752000
H	-3.329148000	-1.572728000	-5.211839000
H	5.874759000	-1.933537000	1.892468000
H	4.460912000	-2.949590000	2.293568000
H	4.223357000	-1.387627000	1.478639000
H	-6.330392000	-0.752029000	-5.074864000
H	-6.080250000	0.614530000	-6.189846000
H	-6.840240000	0.890453000	-4.599137000
H	-3.320839000	-0.655648000	1.442870000
H	-3.339820000	0.031049000	1.848312000

1b_H2_oxadd_ts
SCF = -1973.20936262
H(0 K) = -1971.934478
H(298 K) = -1971.811374
G(298 K) = -1972.112655
SCF+D3 = -1973.51539061
PCM SCF (Benzene) = -1973.21347846
BS2 (def2-tzvp) = -24397.0525643
Low Freq. = -677.2002cm-1, 8.4514cm-1

C	5.772578000	1.825244000	5.105879000
C	5.864290000	3.047952000	4.422654000
C	4.897793000	3.381364000	3.459333000
C	3.849487000	2.491790000	3.175832000
C	3.745271000	1.264821000	3.867263000
C	4.715901000	0.939434000	4.836393000
P	2.296893000	0.145208000	3.464374000
C	2.794791000	-1.488811000	4.234520000
C	2.693083000	-1.716106000	5.625196000
C	3.103877000	-2.940774000	6.175694000
C	3.619616000	-3.950765000	5.346932000
C	3.721464000	-3.732577000	3.963868000
C	3.308319000	-2.510224000	3.410100000
Rh	1.856198000	0.081489000	1.180223000
Ge	0.729607000	-2.056070000	0.193616000
Ge	-1.729388000	-1.547225000	-0.408293000
Si	-3.171292000	-3.546909000	0.059984000
Si	-4.028967000	-3.386681000	2.299003000
C	-4.729955000	-5.087131000	2.840713000
Ge	1.780101000	-0.120931000	-1.441091000
Si	3.917669000	-0.197142000	-2.717088000
Si	3.529554000	-0.999217000	-4.955021000
C	5.099057000	-0.687432000	-6.012629000
Ge	0.814731000	2.094131000	-0.082288000
Ge	-1.660309000	1.606714000	-0.618742000
Si	-3.058927000	3.645164000	-0.272248000
Si	-2.697504000	4.465188000	1.957671000
C	-3.458748000	6.217747000	2.122070000
Ge	-0.750283000	0.153709000	1.544542000
Ge	-0.130975000	-1.599313000	-2.611720000
Ge	-0.033480000	1.297155000	-2.803363000
Ge	-2.412970000	-0.063600000	-2.524032000

C	0.995533000	0.738279000	4.671109000
C	-0.123955000	-0.086871000	4.925093000
C	-1.134209000	0.336183000	5.801789000
C	-1.048519000	1.592022000	6.425849000
C	0.055786000	2.419671000	6.171812000
C	1.074388000	1.996522000	5.300595000
Si	4.822450000	2.027597000	-2.791936000
C	4.824237000	2.848462000	-1.066104000
Si	5.452750000	-1.699729000	-1.631695000
C	6.308702000	-0.890445000	-0.123280000
Si	-1.839993000	-5.547176000	-0.126419000
C	-0.681343000	-5.741623000	1.382902000
Si	-4.972550000	-3.624135000	-1.530674000
C	-5.885083000	-1.952089000	-1.661211000
Si	-2.405944000	5.280898000	-1.909787000
C	-2.343387000	4.537537000	-3.667333000
Si	-5.354760000	3.009624000	-0.557639000
C	-5.735152000	1.440079000	0.463057000
C	-0.783228000	-5.563349000	-1.716369000
C	-3.001486000	-7.073248000	-0.183784000
C	-4.313765000	-4.094525000	-3.262901000
C	-6.250093000	-4.944035000	-0.980323000
C	-5.437536000	-2.099492000	2.415075000
C	-2.651637000	-2.890919000	3.527515000
C	-3.685438000	6.709483000	-1.914543000
C	-0.688204000	6.003153000	-1.483851000
C	3.804295000	3.126251000	-3.980907000
C	6.633893000	1.968452000	-3.417844000
C	4.554572000	-3.286493000	-1.056825000
C	6.835180000	-2.211276000	-2.857972000
C	3.170266000	-2.878104000	-4.942621000
C	2.076794000	-0.115170000	-5.825924000
C	-6.506048000	4.415686000	0.051915000
C	-5.744736000	2.660282000	-2.395933000
C	-3.527495000	3.316257000	3.240018000
C	-0.838566000	4.592753000	2.379192000
H	-4.678983000	6.357176000	-2.238955000
H	-3.362647000	7.497540000	-2.618645000
H	-3.795856000	7.169210000	-0.918709000
H	-5.104020000	-1.104234000	2.078484000
H	-5.785401000	-2.007269000	3.459731000
H	-6.302915000	-2.393850000	1.797805000
H	3.755079000	-3.047035000	-0.334989000
H	5.265557000	-3.978493000	-0.570539000
H	4.091016000	-3.813539000	-1.907346000
H	-1.991274000	-0.318062000	5.991338000
H	-0.197588000	-1.073230000	4.455172000
H	1.936661000	2.645407000	5.121283000
H	-1.409231000	-5.484346000	-2.620532000
H	-0.207709000	-6.504496000	-1.778742000
H	-0.069910000	-4.722317000	-1.725553000
H	-3.559417000	-3.369169000	-3.611459000
H	-5.139832000	-4.103770000	-3.996375000
H	-3.849924000	-5.095097000	-3.263316000
H	2.284369000	-0.938652000	6.278292000
H	3.863541000	2.761466000	-5.020087000
H	4.183423000	4.163863000	-3.964666000
H	2.740312000	3.146700000	-3.689357000
H	-0.708918000	6.562464000	-0.533369000
H	-0.356730000	6.696054000	-2.277839000
H	0.064399000	5.201581000	-1.392520000

H	3.016400000	-3.105447000	7.254968000
H	-1.578875000	3.744536000	-3.735944000
H	-2.086579000	5.323283000	-4.400432000
H	-3.311525000	4.099424000	-3.960838000
H	6.689007000	3.736289000	4.635413000
H	-1.838329000	1.921334000	7.109059000
H	3.366367000	-2.343754000	2.329134000
H	-5.794656000	-5.940805000	-0.862853000
H	-7.052627000	-5.024255000	-1.735665000
H	-6.720181000	-4.669548000	-0.020869000
H	0.134990000	3.397489000	6.658742000
H	4.655350000	-0.009885000	5.376808000
H	-6.281235000	-1.625855000	-0.685693000
H	-6.732790000	-2.038415000	-2.364617000
H	-5.211203000	-1.163535000	-2.037939000
H	-4.623068000	3.290319000	3.115618000
H	-3.309734000	3.668895000	4.263879000
H	-3.143740000	2.285937000	3.149860000
H	3.934680000	-4.906965000	5.777851000
H	-0.370162000	3.593929000	2.396959000
H	-0.710479000	5.043904000	3.379687000
H	-0.294538000	5.213188000	1.648098000
H	-1.805941000	-3.597724000	3.493795000
H	-3.047138000	-2.873813000	4.559227000
H	-2.267789000	-1.884729000	3.283495000
H	-3.676437000	-7.115159000	0.686639000
H	-2.397413000	-7.998542000	-0.189869000
H	-3.620355000	-7.072284000	-1.096546000
H	-7.561994000	4.140353000	-0.121381000
H	-6.312491000	5.363141000	-0.477783000
H	-6.380273000	4.598631000	1.132333000
H	-0.002720000	-4.876132000	1.473548000
H	-0.061072000	-6.648799000	1.268500000
H	-1.248772000	-5.835721000	2.323941000
H	4.013551000	-3.454056000	-4.526014000
H	2.989671000	-3.238090000	-5.971385000
H	2.271659000	-3.102826000	-4.343010000
H	7.282726000	1.412000000	-2.720590000
H	7.036195000	2.993902000	-3.504960000
H	6.710841000	1.489528000	-4.407754000
H	-5.512965000	-5.445906000	2.152228000
H	-5.176487000	-5.005025000	3.848131000
H	-3.939059000	-5.854458000	2.885964000
H	3.111090000	2.737950000	2.404460000
H	4.963729000	4.330525000	2.917245000
H	4.114444000	-4.518003000	3.309823000
H	6.431213000	-2.773347000	-3.716244000
H	7.567459000	-2.860760000	-2.345090000
H	7.378041000	-1.334670000	-3.248998000
H	3.796568000	2.964364000	-0.681584000
H	5.284168000	3.851544000	-1.126622000
H	5.393176000	2.252501000	-0.333405000
H	6.526359000	1.554792000	5.853045000
H	-2.944533000	6.940970000	1.467006000
H	-3.356555000	6.575566000	3.162505000
H	-4.530575000	6.228335000	1.864334000
H	-5.085733000	1.873124000	-2.800732000
H	-5.616220000	3.564236000	-3.014631000
H	-6.789718000	2.319611000	-2.507238000
H	6.906435000	-0.013143000	-0.422761000
H	6.990776000	-1.616260000	0.355463000

H	5.577608000	-0.560695000	0.632960000
H	1.116940000	-0.333824000	-5.328723000
H	2.003655000	-0.461098000	-6.872967000
H	2.209146000	0.979297000	-5.835834000
H	-6.790096000	1.139925000	0.332405000
H	-5.555812000	1.606473000	1.538040000
H	-5.098578000	0.601153000	0.136425000
H	5.289713000	0.391906000	-6.136579000
H	4.957722000	-1.119366000	-7.019897000
H	5.999455000	-1.144820000	-5.571556000
H	3.300652000	0.718650000	0.901619000
H	3.348933000	-0.491103000	1.006732000

1b_H_H
SCF = -1973.22553712
H(0 K) = -1971.948363
H(298 K) = -1971.825526
G(298 K) = -1972.124999
SCF+D3 = -1973.53708679
PCM SCF (Benzene) = -1973.22961216
BS2 (def2-tzvp) = -24397.0684420
Low Freq. = 6.6578cm-1, 11.2512cm-1

C	2.554154000	3.000189000	6.233521000
C	1.264823000	3.545458000	6.330727000
C	0.266557000	3.129565000	5.434617000
C	0.560634000	2.182144000	4.441479000
C	1.852570000	1.623655000	4.340793000
C	2.846807000	2.039712000	5.250704000
P	2.184250000	0.298450000	3.049245000
C	4.063481000	0.218674000	3.091025000
C	4.750562000	-0.637757000	3.979049000
C	6.154135000	-0.620116000	4.042961000
C	6.888985000	0.258314000	3.231844000
C	6.212777000	1.119932000	2.352686000
C	4.811644000	1.096400000	2.280007000
Rh	1.205389000	0.775471000	0.980506000
Ge	2.174113000	1.346625000	-1.527021000
Ge	1.389844000	-1.105140000	-1.103613000
Si	2.983094000	-2.825742000	-2.001537000
Si	5.196078000	-2.369362000	-1.175366000
C	6.494653000	-3.343120000	-2.196962000
Ge	-0.268410000	2.094790000	-0.820571000
Si	-0.434576000	4.569061000	-1.135653000
Si	-0.072806000	5.065572000	-3.463054000
C	-0.592608000	6.876385000	-3.821273000
Ge	-1.424676000	1.002725000	1.330142000
Ge	-2.477519000	-1.024544000	0.044120000
Si	-4.617999000	-2.131421000	0.609881000
Si	-5.872414000	-0.511702000	1.867411000
C	-7.683310000	-1.103538000	2.076235000
Ge	-0.089147000	-1.577723000	1.066589000
Ge	-0.014844000	0.410023000	-3.054600000
Ge	-2.377387000	0.782141000	-1.836395000
Ge	-1.042618000	-1.855210000	-1.983137000
C	1.731186000	-1.254472000	3.997356000
C	2.048522000	-2.511628000	3.433131000
C	1.721926000	-3.698896000	4.105675000
C	1.051161000	-3.649240000	5.338873000
C	0.717844000	-2.406463000	5.898131000
C	1.059586000	-1.214511000	5.235864000

Si	-2.623794000	5.308970000	-0.470899000
C	-3.081911000	4.687163000	1.276526000
Si	1.233436000	5.689045000	0.188694000
C	0.751472000	5.683789000	2.038396000
Si	3.030957000	-2.677242000	-4.405995000
C	3.894002000	-1.062495000	-4.959898000
Si	2.318761000	-5.040985000	-1.350471000
C	1.944420000	-5.135759000	0.522760000
Si	-5.743972000	-2.701956000	-1.435587000
C	-4.617205000	-3.706663000	-2.605537000
Si	-4.127499000	-4.079774000	1.926350000
C	-2.905758000	-3.673722000	3.336411000
C	1.292653000	-2.732504000	-5.196707000
C	4.015135000	-4.164935000	-5.111201000
C	0.763688000	-5.605964000	-2.309491000
C	3.733198000	-6.283889000	-1.715444000
C	5.369927000	-2.904068000	0.652350000
C	5.630606000	-0.513252000	-1.301421000
C	-7.285387000	-3.767973000	-1.034477000
C	-6.316046000	-1.121846000	-2.347380000
C	-3.947733000	4.698639000	-1.708509000
C	-2.674357000	7.225857000	-0.432613000
C	2.957753000	4.885190000	0.019146000
C	1.374604000	7.511143000	-0.392488000
C	1.776004000	4.870861000	-3.912042000
C	-1.101376000	3.937790000	-4.611992000
C	-5.747432000	-4.746319000	2.704240000
C	-3.365950000	-5.452743000	0.834925000
C	-5.105836000	-0.266925000	3.602277000
C	-5.889253000	1.176064000	0.973521000
H	-6.999917000	-4.729778000	-0.576150000
H	-7.840540000	-3.990565000	-1.963517000
H	-7.973510000	-3.253663000	-0.343625000
H	4.685200000	-2.330937000	1.298992000
H	6.397800000	-2.713469000	1.008871000
H	5.158332000	-3.978183000	0.786281000
H	2.939104000	3.852728000	0.406070000
H	3.698858000	5.460563000	0.603281000
H	3.295141000	4.853228000	-1.029812000
H	1.978841000	-4.661547000	3.652358000
H	2.569101000	-2.564143000	2.471151000
H	0.806847000	-0.253198000	5.691752000
H	0.720429000	-3.615932000	-4.868937000
H	1.386089000	-2.772521000	-6.297180000
H	0.702941000	-1.836684000	-4.938740000
H	-0.071630000	-4.897420000	-2.173680000
H	0.433178000	-6.597933000	-1.952528000
H	0.964259000	-5.686106000	-3.391122000
H	4.190976000	-1.319788000	4.625420000
H	-3.783325000	5.118740000	-2.715041000
H	-4.955177000	5.004684000	-1.373997000
H	-3.937535000	3.598338000	-1.795315000
H	-7.057459000	-0.558493000	-1.756403000
H	-6.783933000	-1.387547000	-3.312255000
H	-5.465523000	-0.450473000	-2.555815000
H	6.670764000	-1.295902000	4.732842000
H	-3.713442000	-3.136825000	-2.880908000
H	-5.162340000	-3.953714000	-3.534277000
H	-4.288054000	-4.651045000	-2.141689000
H	1.039388000	4.293759000	7.097793000
H	0.788633000	-4.575955000	5.859661000

H	4.289850000	1.761360000	1.583475000
H	4.029775000	-6.270342000	-2.777012000
H	3.405224000	-7.309665000	-1.467991000
H	4.628490000	-6.063123000	-1.110035000
H	0.195736000	-2.357318000	6.859581000
H	3.853954000	1.617293000	5.194283000
H	2.846377000	-4.903905000	1.114632000
H	1.608933000	-6.152883000	0.794353000
H	1.152735000	-4.422467000	0.810940000
H	-5.137724000	-1.194214000	4.198372000
H	-5.660348000	0.511513000	4.156556000
H	-4.053808000	0.056144000	3.523568000
H	7.982804000	0.271063000	3.283330000
H	-4.865968000	1.574723000	0.872032000
H	-6.485272000	1.906087000	1.550077000
H	-6.325049000	1.099879000	-0.036122000
H	5.548604000	-0.144168000	-2.336980000
H	6.666169000	-0.343105000	-0.955805000
H	4.958884000	0.091324000	-0.669430000
H	5.027674000	-4.239720000	-4.683148000
H	4.115594000	-4.055937000	-6.206284000
H	3.491647000	-5.116326000	-4.917545000
H	-5.541653000	-5.671966000	3.271461000
H	-6.505749000	-4.979743000	1.938653000
H	-6.185973000	-4.014774000	3.403584000
H	3.383838000	-0.176551000	-4.545205000
H	3.876279000	-0.979435000	-6.061389000
H	4.947676000	-1.031964000	-4.635532000
H	2.402895000	5.597919000	-3.368853000
H	1.925783000	5.036361000	-4.994044000
H	2.140786000	3.858480000	-3.668042000
H	-1.991316000	7.629864000	0.333433000
H	-3.695524000	7.567511000	-0.184659000
H	-2.396028000	7.667658000	-1.403277000
H	6.286823000	-4.425739000	-2.207359000
H	7.498487000	-3.194867000	-1.759220000
H	6.530495000	-2.992781000	-3.242005000
H	-0.219188000	1.879391000	3.732664000
H	-0.743147000	3.548399000	5.498882000
H	6.775229000	1.809585000	1.714730000
H	1.754027000	7.573572000	-1.426187000
H	2.081540000	8.057102000	0.258139000
H	0.405222000	8.034721000	-0.353700000
H	-3.047221000	3.585494000	1.334466000
H	-4.103343000	5.014638000	1.541262000
H	-2.387714000	5.083094000	2.036395000
H	3.340986000	3.320434000	6.924814000
H	-8.197886000	-1.170465000	1.102966000
H	-8.243787000	-0.387006000	2.703412000
H	-7.740629000	-2.093078000	2.559135000
H	-2.457165000	-5.096193000	0.322418000
H	-4.076590000	-5.800552000	0.066611000
H	-3.087708000	-6.322911000	1.456228000
H	-0.193275000	6.224892000	2.214570000
H	1.539851000	6.174929000	2.636772000
H	0.633880000	4.654004000	2.415101000
H	-0.790515000	2.882447000	-4.524281000
H	-0.961904000	4.245982000	-5.663974000
H	-2.177959000	3.990438000	-4.380061000
H	-2.729803000	-4.571779000	3.955492000
H	-3.291564000	-2.876558000	3.992995000

H	-1.933755000	-3.340263000	2.934273000
H	-1.678815000	7.014337000	-3.688483000
H	-0.348506000	7.131967000	-4.868266000
H	-0.074841000	7.596795000	-3.167236000
H	0.968032000	2.167514000	1.642930000
H	2.598720000	1.510451000	0.638595000

2b

SCF = -1972.45651221
H(0 K) = -1971.186498
H(298 K) = -1971.063305
G(298 K) = -1971.368302
SCF+D3 = -1972.76166889
PCM SCF (Benzene) = -1972.48205522
BS2 (def2-tzvp) = -24396.2923555
Low Freq. = 6.0160cm-1, 7.3463cm-1

Ge	-2.300764000	-1.373171000	-0.089703000
Ge	-2.396972000	0.365858000	-2.096533000
Ge	-1.755610000	0.897357000	1.096767000
Ge	0.105845000	-1.607503000	0.963887000
Ge	-0.796894000	-1.950134000	-2.185314000
Ge	0.103255000	0.454424000	-3.097401000
Ge	-0.675088000	2.102919000	-0.963119000
Ge	1.546137000	-1.094026000	-1.198154000
Ge	1.849644000	1.449634000	-0.928295000
Rh	0.826671000	0.813192000	1.223837000
P	1.962697000	0.598652000	3.284269000
Si	-4.223203000	-2.793522000	0.596922000
Si	-6.167494000	-1.372101000	0.524443000
Si	-3.715407000	-3.505089000	2.841807000
Si	-4.351149000	-4.640369000	-0.946875000
C	-7.714325000	-2.457492000	0.816938000
H	-7.660985000	-3.010022000	1.769040000
H	-7.852488000	-3.189488000	0.004003000
H	-8.613880000	-1.817142000	0.847029000
C	-6.064685000	-0.043397000	1.893106000
H	-6.935821000	0.632390000	1.827338000
H	-5.153084000	0.570713000	1.795067000
H	-6.066060000	-0.496190000	2.898468000
C	-6.335451000	-0.512494000	-1.170477000
H	-7.250636000	0.105745000	-1.186236000
H	-6.405932000	-1.242897000	-1.992897000
H	-5.476439000	0.147751000	-1.378534000
C	-5.298670000	-4.238159000	3.623427000
H	-5.709286000	-5.066414000	3.023097000
H	-6.085660000	-3.473908000	3.733869000
H	-5.069402000	-4.631704000	4.629719000
C	-2.348236000	-4.839422000	2.833694000
H	-2.095701000	-5.124396000	3.870645000
H	-1.427997000	-4.469730000	2.350024000
H	-2.670890000	-5.751061000	2.304318000
C	-3.123578000	-2.036419000	3.909850000
H	-3.871379000	-1.227411000	3.945237000
H	-2.181722000	-1.612893000	3.521167000
H	-2.939197000	-2.375884000	4.944689000
C	-5.613823000	-5.896405000	-0.251018000
H	-5.736547000	-6.730745000	-0.964460000
H	-6.604793000	-5.440831000	-0.091822000
H	-5.275892000	-6.323176000	0.707957000
C	-2.657483000	-5.500082000	-1.130734000

H	-2.271522000	-5.858986000	-0.162819000
H	-1.909840000	-4.815113000	-1.566291000
H	-2.752428000	-6.370115000	-1.804507000
C	-4.936224000	-4.039322000	-2.661285000
H	-4.259902000	-3.273803000	-3.076970000
H	-5.951572000	-3.611989000	-2.617594000
H	-4.959296000	-4.888317000	-3.367403000
Si	-1.031372000	4.556316000	-1.271743000
Si	-0.659102000	5.638575000	0.846942000
Si	0.485937000	5.341817000	-2.971504000
Si	-3.333805000	4.767635000	-1.960163000
Si	3.486715000	-2.440066000	-2.040534000
C	1.045540000	5.174609000	1.563249000
H	1.123657000	4.089237000	1.743396000
H	1.862501000	5.462927000	0.881780000
H	1.205081000	5.692947000	2.525467000
C	-0.714164000	7.530571000	0.575270000
H	-1.664200000	7.859556000	0.124156000
H	0.108602000	7.867560000	-0.076886000
H	-0.604041000	8.045779000	1.546254000
C	-2.018840000	5.146797000	2.095637000
H	-1.811352000	5.604451000	3.079436000
H	-2.063084000	4.052135000	2.229848000
H	-3.014080000	5.489970000	1.767916000
C	2.272604000	5.415807000	-2.297789000
H	2.608763000	4.429372000	-1.933767000
H	2.362806000	6.134908000	-1.466817000
H	2.963615000	5.735404000	-3.098003000
C	0.449629000	4.219178000	-4.514063000
H	1.122064000	4.627633000	-5.289392000
H	-0.562507000	4.150431000	-4.944922000
H	0.786786000	3.194858000	-4.279533000
C	-0.061229000	7.101219000	-3.483193000
H	0.650914000	7.509452000	-4.222379000
H	-0.091115000	7.793325000	-2.625896000
H	-1.059743000	7.092991000	-3.950948000
C	-4.485543000	3.739022000	-0.836463000
H	-5.537037000	3.894589000	-1.136545000
H	-4.387316000	4.026894000	0.223040000
H	-4.267670000	2.660478000	-0.921063000
C	-3.553709000	4.199704000	-3.769419000
H	-4.615931000	4.279590000	-4.061382000
H	-3.244241000	3.149910000	-3.907445000
H	-2.967368000	4.824175000	-4.463721000
C	-3.834500000	6.608449000	-1.821001000
H	-3.171054000	7.264493000	-2.407747000
H	-3.821305000	6.955059000	-0.774369000
H	-4.862353000	6.739071000	-2.204056000
Si	3.112605000	-4.730932000	-1.401686000
Si	5.539293000	-1.587592000	-1.113386000
Si	3.513673000	-2.205586000	-4.444889000
C	2.558726000	-4.851720000	0.421824000
H	1.577522000	-4.368105000	0.570695000
H	3.282131000	-4.364820000	1.096216000
H	2.464960000	-5.911255000	0.719721000
C	4.754568000	-5.690454000	-1.608223000
H	5.538218000	-5.307317000	-0.933713000
H	5.135390000	-5.634373000	-2.641125000
H	4.594124000	-6.756140000	-1.365933000
C	1.781332000	-5.535541000	-2.508529000
H	1.597490000	-6.574361000	-2.181347000

H	2.097981000	-5.567884000	-3.564189000
H	0.823897000	-4.989852000	-2.456163000
C	5.603285000	0.320876000	-1.181018000
H	4.796207000	0.769778000	-0.576645000
H	5.501709000	0.696299000	-2.212649000
H	6.568708000	0.679445000	-0.781354000
C	7.004385000	-2.270254000	-2.135790000
H	6.998529000	-1.882413000	-3.167688000
H	6.998385000	-3.371487000	-2.182266000
H	7.954548000	-1.958221000	-1.666419000
C	5.739982000	-2.154601000	0.695913000
H	6.671165000	-1.739373000	1.120492000
H	5.800596000	-3.253271000	0.767677000
H	4.899997000	-1.819065000	1.325653000
C	4.079919000	-0.449561000	-4.938929000
H	4.087867000	-0.354927000	-6.039350000
H	5.097464000	-0.229189000	-4.575894000
H	3.398055000	0.321056000	-4.540974000
C	4.763332000	-3.487779000	-5.119952000
H	4.425532000	-4.519673000	-4.927235000
H	5.767337000	-3.368199000	-4.682670000
H	4.853619000	-3.365310000	-6.214181000
C	1.820624000	-2.558505000	-5.251315000
H	1.075235000	-1.788825000	-4.989829000
H	1.416801000	-3.539370000	-4.951756000
H	1.932916000	-2.557717000	-6.350362000
C	3.018196000	2.119348000	3.501378000
C	3.885064000	2.501349000	2.454131000
H	3.908174000	1.921093000	1.524961000
C	4.713918000	3.623654000	2.595647000
H	5.384638000	3.908978000	1.778919000
C	4.677042000	4.381536000	3.778536000
H	5.320021000	5.260735000	3.886648000
C	3.813019000	4.009247000	4.820174000
H	3.781322000	4.594747000	5.744598000
C	2.986479000	2.880832000	4.687634000
H	2.323340000	2.594025000	5.508717000
C	3.149122000	-0.800078000	3.629432000
C	4.459452000	-0.552797000	4.091770000
H	4.825699000	0.473100000	4.185551000
C	5.294666000	-1.624262000	4.451046000
H	6.308347000	-1.419607000	4.809939000
C	4.831358000	-2.945511000	4.359890000
H	5.482514000	-3.777243000	4.646694000
C	3.527600000	-3.197585000	3.899048000
H	3.157016000	-4.224811000	3.825725000
C	2.693018000	-2.132247000	3.530287000
H	1.677226000	-2.338823000	3.173942000
C	0.875023000	0.581874000	4.802417000
C	-0.333226000	1.309686000	4.822305000
H	-0.666800000	1.839125000	3.924224000
C	-1.116157000	1.356909000	5.985616000
H	-2.050971000	1.926446000	5.984838000
C	-0.706658000	0.670520000	7.140271000
H	-1.321532000	0.701658000	8.045303000
C	0.493200000	-0.057506000	7.128011000
H	0.821260000	-0.594202000	8.023909000
C	1.284195000	-0.102054000	5.968525000
H	2.220735000	-0.666068000	5.977150000
H	0.077867000	1.936485000	2.030954000

2b_H2_diss_ts
SCF = -1973.61430499
H(0 K) = -1972.331332
H(298 K) = -1972.207070
G(298 K) = -1972.510484
SCF+D3 = -1973.92487574
PCM SCF (Benzene) = -1973.63994308
BS2 (def2-tzvp) = -24397.4492172
Low Freq. = -240.1228cm-1, 6.2108cm-1

C	-3.833539000	-1.042102000	6.153169000
C	-4.601797000	-1.853620000	5.300782000
C	-4.308562000	-1.897674000	3.930538000
C	-3.248230000	-1.136437000	3.406805000
C	-2.464266000	-0.334739000	4.255972000
C	-2.772453000	-0.285724000	5.636787000
P	-0.995754000	0.644251000	3.638179000
C	-1.255370000	2.229558000	4.607413000
C	-0.253508000	2.834238000	5.391422000
C	-0.512557000	4.041885000	6.062730000
C	-1.767623000	4.659506000	5.956426000
C	-2.775212000	4.057667000	5.184261000
C	-2.523220000	2.851157000	4.514234000
Rh	-0.549176000	1.145159000	1.313212000
Ge	-1.413818000	2.286681000	-0.939177000
Ge	1.142678000	1.909199000	-0.863113000
Si	2.461306000	4.016709000	-1.137015000
Si	2.594576000	5.108870000	1.004628000
C	3.351542000	6.847127000	0.755717000
Ge	-2.052757000	-0.197886000	-0.999144000
Si	-4.419819000	-0.615709000	-1.736868000
Si	-4.556781000	0.019068000	-4.065558000
C	-6.260963000	-0.547338000	-4.725597000
Ge	-0.713681000	-1.422903000	0.927165000
Ge	1.497066000	-2.033218000	-0.375551000
Si	2.828174000	-4.122676000	-0.201708000
Si	1.763495000	-5.514491000	1.449443000
C	2.430448000	-7.294663000	1.243403000
Ge	-0.278614000	0.767938000	-3.043827000
Ge	-0.331078000	-1.826541000	-2.258317000
Ge	1.863931000	0.196837000	1.004019000
Ge	2.071860000	-0.231909000	-2.205124000
C	0.449713000	-0.192510000	4.480237000
C	1.738805000	0.384084000	4.403312000
C	2.830525000	-0.217693000	5.048973000
C	2.657658000	-1.419053000	5.755138000
C	1.388009000	-2.013474000	5.811369000
C	0.288144000	-1.405125000	5.182541000
Si	-4.899739000	-2.957898000	-1.469364000
C	-4.390509000	-3.553887000	0.272951000
Si	-5.932677000	0.756991000	-0.448947000
C	-6.316554000	-0.027534000	1.249135000
Si	1.364101000	5.405124000	-2.772964000
C	-0.217601000	6.185179000	-2.039744000
Si	4.654998000	3.340186000	-1.874395000
C	5.320210000	1.890524000	-0.822248000
Si	2.772637000	-5.154613000	-2.381606000
C	3.150794000	-3.900490000	-3.769249000
Si	5.057693000	-3.477151000	0.436824000
C	5.025074000	-2.244227000	1.894372000
C	0.904509000	4.411317000	-4.336055000

C	2.576991000	6.797497000	-3.270698000
C	4.625706000	2.814183000	-3.709479000
C	5.842234000	4.826832000	-1.675780000
C	3.709893000	4.106827000	2.191523000
C	0.873411000	5.300503000	1.804180000
C	4.112196000	-6.519198000	-2.404521000
C	1.062868000	-5.942965000	-2.697724000
C	-3.981294000	-4.002829000	-2.776719000
C	-6.783328000	-3.225954000	-1.668260000
C	-5.227069000	2.508964000	-0.165335000
C	-7.580111000	0.915974000	-1.407035000
C	-4.405414000	1.914688000	-4.244063000
C	-3.221520000	-0.816655000	-5.143452000
C	5.997334000	-5.052822000	0.976144000
C	5.968676000	-2.676788000	-1.037632000
C	2.168507000	-4.908178000	3.214276000
C	-0.130674000	-5.527485000	1.210861000
H	5.124273000	-6.095276000	-2.295562000
H	4.075731000	-7.054799000	-3.369948000
H	3.962583000	-7.260054000	-1.602215000
H	3.346458000	3.069723000	2.296842000
H	3.714189000	4.573553000	3.192793000
H	4.752462000	4.066233000	1.834915000
H	-4.307661000	2.477211000	0.443292000
H	-5.968611000	3.129192000	0.369499000
H	-4.987665000	3.011846000	-1.116869000
H	3.817192000	0.252526000	4.991725000
H	1.896052000	1.311163000	3.842344000
H	-0.695398000	-1.876990000	5.248539000
H	1.789548000	3.942653000	-4.796565000
H	0.447384000	5.082773000	-5.084609000
H	0.177072000	3.612501000	-4.110923000
H	3.934138000	1.971625000	-3.879452000
H	5.634355000	2.491876000	-4.023742000
H	4.320171000	3.646253000	-4.365325000
H	0.724157000	2.359302000	5.505358000
H	-4.331073000	-3.772199000	-3.796615000
H	-4.161816000	-5.077236000	-2.594890000
H	-2.891829000	-3.830454000	-2.743650000
H	0.835378000	-6.733153000	-1.962977000
H	1.040847000	-6.401395000	-3.702356000
H	0.258968000	-5.188790000	-2.653224000
H	0.272628000	4.493539000	6.677501000
H	2.364520000	-3.130217000	-3.842054000
H	3.199072000	-4.423946000	-4.740711000
H	4.114714000	-3.390140000	-3.609864000
H	-5.429289000	-2.445338000	5.704755000
H	3.509386000	-1.890195000	6.255502000
H	-3.328303000	2.376283000	3.942994000
H	5.483688000	5.717180000	-2.217652000
H	6.834979000	4.562306000	-2.081607000
H	5.976146000	5.101463000	-0.616337000
H	1.244133000	-2.954196000	6.351958000
H	-2.180164000	0.342513000	6.309165000
H	5.344797000	2.142044000	0.250744000
H	6.348171000	1.638474000	-1.137983000
H	4.698267000	0.987398000	-0.949653000
H	3.251509000	-4.954640000	3.416878000
H	1.661291000	-5.552530000	3.954623000
H	1.833687000	-3.870998000	3.380611000
H	-1.964883000	5.599406000	6.481230000

H	-0.558303000	-4.524033000	1.381174000
H	-0.597930000	-6.222205000	1.931289000
H	-0.411022000	-5.850248000	0.194899000
H	0.172162000	5.837454000	1.144534000
H	0.958522000	5.868175000	2.747906000
H	0.437384000	4.315302000	2.037337000
H	2.910694000	7.387878000	-2.401855000
H	2.079010000	7.486388000	-3.976136000
H	3.471167000	6.393425000	-3.773832000
H	7.040121000	-4.794393000	1.232912000
H	6.025414000	-5.808159000	0.173768000
H	5.538007000	-5.516025000	1.865128000
H	-0.925641000	5.412772000	-1.692796000
H	-0.728686000	6.792553000	-2.807779000
H	0.013296000	6.845440000	-1.187347000
H	-5.208381000	2.442315000	-3.703179000
H	-4.472419000	2.199654000	-5.309010000
H	-3.437252000	2.278453000	-3.859412000
H	-7.355308000	-2.694145000	-0.889732000
H	-7.017006000	-4.301996000	-1.580741000
H	-7.145610000	-2.882200000	-2.650841000
H	4.331811000	6.806776000	0.253948000
H	3.493591000	7.332325000	1.737979000
H	2.687544000	7.493089000	0.157598000
H	-3.033193000	-1.169998000	2.333242000
H	-4.904723000	-2.524268000	3.260732000
H	-3.763657000	4.521799000	5.108258000
H	-7.453125000	1.467631000	-2.352804000
H	-8.307684000	1.472750000	-0.789635000
H	-8.020047000	-0.067885000	-1.638818000
H	-3.308039000	-3.428518000	0.447599000
H	-4.633579000	-4.623561000	0.399962000
H	-4.932920000	-2.987069000	1.047740000
H	-4.059504000	-0.996433000	7.223246000
H	2.165801000	-7.717812000	0.260334000
H	1.991356000	-7.947417000	2.018832000
H	3.526945000	-7.336253000	1.349818000
H	5.435515000	-1.785883000	-1.409873000
H	6.080617000	-3.383147000	-1.876878000
H	6.980820000	-2.361081000	-0.727940000
H	-6.766214000	-1.028586000	1.139777000
H	-7.039984000	0.604888000	1.794352000
H	-5.414480000	-0.123025000	1.874812000
H	-2.208715000	-0.455894000	-4.896758000
H	-3.411721000	-0.581961000	-6.206147000
H	-3.228114000	-1.913148000	-5.031277000
H	6.056736000	-2.015553000	2.216232000
H	4.477176000	-2.649610000	2.760670000
H	4.542620000	-1.295362000	1.602872000
H	-6.347086000	-1.646714000	-4.720962000
H	-6.372767000	-0.209277000	-5.771468000
H	-7.100877000	-0.134276000	-4.145333000
H	-1.841936000	3.100474000	2.229527000
H	0.645514000	1.872327000	2.031953000
H	-1.769435000	2.956488000	1.475540000

2b_H2

SCF = -1973.62379228
H(0 K) = -1972.339141
H(298 K) = -1972.215185
G(298 K) = -1972.519446

SCF+D3 = -1973.93754732
PCM SCF (Benzene) = -1973.64952477
BS2 (def2-tzvp) = -24397.4575056
Low Freq. = 7.0700cm-1, 8.2862cm-1

C	1.808412000	2.605061000	6.541015000
C	1.621132000	3.843803000	5.903140000
C	1.353204000	3.886352000	4.527667000
C	1.268269000	2.694525000	3.786242000
C	1.439842000	1.451028000	4.420797000
C	1.720710000	1.414345000	5.806876000
P	1.299341000	-0.166115000	3.496159000
C	2.779296000	-1.068908000	4.210310000
C	2.664037000	-2.172690000	5.078954000
C	3.816079000	-2.780054000	5.609327000
C	5.089665000	-2.291072000	5.285760000
C	5.211165000	-1.179424000	4.434586000
C	4.065639000	-0.573028000	3.900673000
Rh	1.404888000	-0.248443000	1.099689000
Ge	2.668640000	-0.264685000	-1.467174000
Ge	0.729149000	-1.916033000	-1.015417000
Si	1.453439000	-4.274357000	-1.462474000
Si	3.315672000	-4.780106000	-0.018772000
C	4.111618000	-6.412881000	-0.616948000
Ge	1.100945000	1.756435000	-0.880799000
Si	2.264652000	3.932652000	-1.369681000
Si	2.688463000	4.024013000	-3.747846000
C	3.223067000	5.810425000	-4.175338000
Ge	-0.690959000	1.494887000	1.049462000
Ge	-2.589992000	0.269728000	-0.322931000
Si	-5.031804000	0.512638000	0.013148000
Si	-5.361863000	2.023857000	1.859376000
C	-7.191896000	2.576974000	1.851538000
Ge	0.511987000	0.050611000	-3.144794000
Ge	-1.305223000	1.694204000	-2.079008000
Ge	-0.972724000	-1.438548000	0.979931000
Ge	-1.602980000	-1.275892000	-2.168262000
C	-0.176569000	-0.983514000	4.283025000
C	-0.424079000	-2.356835000	4.044955000
C	-1.535293000	-2.989012000	4.625974000
C	-2.429946000	-2.253302000	5.419637000
C	-2.208026000	-0.883747000	5.632722000
C	-1.086084000	-0.249567000	5.072449000
Si	0.827918000	5.748652000	-0.712576000
C	0.195937000	5.506754000	1.073908000
Si	4.367635000	4.013559000	-0.189964000
C	4.107545000	4.364792000	1.670057000
Si	2.132222000	-4.371740000	-3.776397000
C	3.815010000	-3.501495000	-4.019705000
Si	-0.404285000	-5.751372000	-1.052939000
C	-1.190641000	-5.440068000	0.660275000
Si	-5.873940000	1.425054000	-2.055680000
C	-5.177397000	0.492052000	-3.568046000
Si	-5.895813000	-1.693850000	0.445884000
C	-4.892384000	-2.566015000	1.815295000
C	0.844990000	-3.564470000	-4.932143000
C	2.309143000	-6.212147000	-4.265861000
C	-1.742480000	-5.533727000	-2.398346000
C	0.230149000	-7.555657000	-1.091910000
C	2.724828000	-4.999012000	1.785568000
C	4.644184000	-3.409764000	-0.056853000

C	-7.776913000	1.240727000	-2.039302000
C	-5.422413000	3.274405000	-2.199262000
C	-0.668349000	5.884432000	-1.891214000
C	1.819294000	7.383668000	-0.768581000
C	5.361170000	2.391152000	-0.372489000
C	5.420909000	5.431227000	-0.924402000
C	4.091255000	2.819742000	-4.228919000
C	1.143010000	3.618691000	-4.792852000
C	-7.709940000	-1.518525000	1.022756000
C	-5.837386000	-2.751040000	-1.142441000
C	-4.983730000	1.150637000	3.515315000
C	-4.245063000	3.562241000	1.694801000
H	-8.082032000	0.181157000	-2.043766000
H	-8.201055000	1.714952000	-2.942293000
H	-8.231593000	1.724616000	-1.159476000
H	2.264590000	-4.069647000	2.162725000
H	3.584019000	-5.236042000	2.437705000
H	1.990374000	-5.815792000	1.881185000
H	4.837302000	1.532694000	0.080680000
H	6.338221000	2.496372000	0.132580000
H	5.550785000	2.146997000	-1.430433000
H	-1.703175000	-4.055614000	4.448054000
H	0.263600000	-2.941569000	3.424929000
H	-0.915805000	0.813639000	5.262394000
H	-0.161323000	-3.991813000	-4.791526000
H	1.139379000	-3.725157000	-5.984657000
H	0.776530000	-2.475242000	-4.766955000
H	-2.105684000	-4.492562000	-2.448096000
H	-2.607657000	-6.184571000	-2.179583000
H	-1.361159000	-5.807222000	-3.396159000
H	1.680000000	-2.556289000	5.360336000
H	-0.351516000	6.124812000	-2.919636000
H	-1.346819000	6.687072000	-1.551662000
H	-1.245486000	4.944180000	-1.925582000
H	-5.871583000	3.868068000	-1.385843000
H	-5.797724000	3.677721000	-3.156567000
H	-4.330144000	3.426711000	-2.174508000
H	3.709450000	-3.635006000	6.284732000
H	-4.085707000	0.623830000	-3.657159000
H	-5.638695000	0.884213000	-4.491841000
H	-5.387447000	-0.588750000	-3.516013000
H	1.689842000	4.772513000	6.478390000
H	-3.297213000	-2.746411000	5.869636000
H	4.180006000	0.306620000	3.258698000
H	0.739028000	-7.797019000	-2.038998000
H	-0.622497000	-8.248972000	-0.980088000
H	0.932960000	-7.754288000	-0.265737000
H	-2.903114000	-0.302564000	6.246643000
H	1.874133000	0.455196000	6.311391000
H	-0.458816000	-5.596155000	1.470321000
H	-2.030279000	-6.139794000	0.820099000
H	-1.581536000	-4.411653000	0.748376000
H	-5.674048000	0.309367000	3.693400000
H	-5.102627000	1.865310000	4.349357000
H	-3.953049000	0.759629000	3.545077000
H	5.984083000	-2.765587000	5.701329000
H	-3.177644000	3.285072000	1.738362000
H	-4.448689000	4.264091000	2.523079000
H	-4.421372000	4.094974000	0.746148000
H	5.012605000	-3.223122000	-1.078653000
H	5.504674000	-3.709658000	0.567742000

H	4.247565000	-2.462294000	0.344854000
H	3.009079000	-6.753705000	-3.608961000
H	2.693476000	-6.281064000	-5.299215000
H	1.337688000	-6.732845000	-4.235877000
H	-8.146161000	-2.520515000	1.183805000
H	-8.331429000	-0.996734000	0.276845000
H	-7.781029000	-0.965396000	1.974058000
H	3.781884000	-2.448285000	-3.691846000
H	4.091704000	-3.512902000	-5.088926000
H	4.618452000	-4.007742000	-3.459419000
H	5.042789000	3.084713000	-3.738949000
H	4.254986000	2.852843000	-5.320730000
H	3.845189000	1.779062000	-3.957299000
H	2.644273000	7.381759000	-0.036775000
H	1.152924000	8.228903000	-0.520204000
H	2.248257000	7.574337000	-1.765484000
H	3.386605000	-7.242280000	-0.648754000
H	4.925541000	-6.701081000	0.072142000
H	4.549022000	-6.302765000	-1.623156000
H	1.066994000	2.735828000	2.710743000
H	1.207910000	4.846362000	4.023316000
H	6.199704000	-0.778681000	4.188575000
H	5.705402000	5.226180000	-1.969546000
H	6.351758000	5.536711000	-0.338847000
H	4.893980000	6.398875000	-0.896144000
H	-0.394721000	4.579593000	1.176025000
H	-0.446529000	6.354286000	1.371345000
H	1.042821000	5.455936000	1.778535000
H	2.026356000	2.564540000	7.612964000
H	-7.438397000	3.152932000	0.944191000
H	-7.386504000	3.226594000	2.723518000
H	-7.881829000	1.719246000	1.909838000
H	-4.810422000	-2.835908000	-1.536070000
H	-6.473006000	-2.325625000	-1.936535000
H	-6.204510000	-3.770781000	-0.929366000
H	3.621404000	5.341675000	1.830338000
H	5.084256000	4.390174000	2.185716000
H	3.487573000	3.593900000	2.156841000
H	0.852211000	2.558294000	-4.701277000
H	1.360721000	3.815207000	-5.858030000
H	0.274965000	4.234005000	-4.504433000
H	-5.328226000	-3.559442000	2.023738000
H	-4.894921000	-1.988201000	2.753852000
H	-3.841271000	-2.711679000	1.513183000
H	2.397720000	6.524457000	-4.017461000
H	3.505930000	5.858475000	-5.242217000
H	4.087373000	6.147495000	-3.581223000
H	2.883358000	0.589232000	1.359225000
H	1.684397000	-1.757433000	1.465446000
H	3.127582000	-0.245040000	1.125724000

2b_H2_oxadd_ts
SCF = -1973.62182862
H(0 K) = -1972.339038
H(298 K) = -1972.215461
G(298 K) = -1972.517500
SCF+D3 = -1973.93751249
PCM SCF (Benzene) = -1973.64747567
BS2 (def2-tzvp) = -24397.4545063
Low Freq. = -640.0738cm-1, 5.0703cm-1

C	1.796771000	2.680513000	6.520775000
C	1.257228000	3.860635000	5.980877000
C	0.823182000	3.881787000	4.647643000
C	0.926675000	2.727332000	3.852066000
C	1.453047000	1.538452000	4.390251000
C	1.894851000	1.524639000	5.733437000
P	1.537409000	-0.041603000	3.397174000
C	3.174840000	-0.734267000	3.981096000
C	3.262863000	-1.726316000	4.980023000
C	4.519203000	-2.161199000	5.436980000
C	5.695483000	-1.606112000	4.912313000
C	5.614073000	-0.607626000	3.927032000
C	4.363531000	-0.176162000	3.462385000
Rh	1.476192000	0.079680000	1.011210000
Ge	2.613580000	0.328239000	-1.602845000
Ge	1.041474000	-1.685506000	-1.072946000
Si	2.187760000	-3.857342000	-1.589651000
Si	4.210385000	-3.982949000	-0.285508000
C	5.265696000	-5.429946000	-0.956978000
Ge	0.676913000	1.963046000	-0.871051000
Si	1.385484000	4.339773000	-1.291832000
Si	1.670196000	4.581807000	-3.679479000
C	1.833450000	6.448026000	-4.067252000
Ge	-0.971012000	1.321104000	1.116749000
Ge	-2.640654000	-0.222708000	-0.253428000
Si	-5.066026000	-0.469958000	0.173212000
Si	-5.621742000	0.978167000	2.016514000
C	-7.525479000	1.138361000	2.083876000
Ge	0.349164000	0.217783000	-3.158980000
Ge	-1.706260000	1.457786000	-1.991177000
Ge	-0.671813000	-1.577153000	0.976403000
Ge	-1.411446000	-1.527079000	-2.129687000
C	0.264252000	-1.123066000	4.217798000
C	0.264630000	-2.515735000	3.966558000
C	-0.697160000	-3.343744000	4.567038000
C	-1.686886000	-2.789472000	5.395210000
C	-1.709262000	-1.404767000	5.624910000
C	-0.737092000	-0.572572000	5.044081000
Si	-0.341067000	5.813127000	-0.488175000
C	-0.784435000	5.417677000	1.327443000
Si	3.488976000	4.784408000	-0.194763000
C	3.247376000	5.060815000	1.679362000
Si	2.708534000	-3.814105000	-3.946275000
C	4.167553000	-2.630349000	-4.290749000
Si	0.693010000	-5.675278000	-1.078195000
C	-0.000911000	-5.537551000	0.696480000
Si	-6.138637000	0.217749000	-1.876037000
C	-5.312382000	-0.583431000	-3.398633000
Si	-5.446314000	-2.797510000	0.665387000
C	-4.245850000	-3.417615000	2.013094000
C	1.212099000	-3.273474000	-5.001574000
C	3.206680000	-5.582124000	-4.479761000
C	-0.762604000	-5.711918000	-2.314226000
C	1.661139000	-7.320004000	-1.208717000
C	3.803406000	-4.312157000	1.552098000
C	5.241114000	-2.380416000	-0.401957000
C	-7.964790000	-0.342349000	-1.797110000
C	-6.070067000	2.116991000	-2.054898000
C	-1.913474000	5.661743000	-1.561096000
C	0.296009000	7.615278000	-0.561953000
C	4.753780000	3.375007000	-0.448630000

C	4.227946000	6.381132000	-0.945256000
C	3.254788000	3.684126000	-4.256586000
C	0.185543000	3.912515000	-4.675925000
C	-7.240601000	-2.982164000	1.298339000
C	-5.219594000	-3.852006000	-0.909458000
C	-5.000776000	0.233436000	3.662188000
C	-4.857619000	2.711110000	1.784691000
H	-8.052134000	-1.441221000	-1.772516000
H	-8.499040000	0.015935000	-2.695090000
H	-8.483319000	0.062698000	-0.912765000
H	3.196635000	-3.493688000	1.975527000
H	4.736394000	-4.374179000	2.139647000
H	3.254505000	-5.258860000	1.686442000
H	4.418276000	2.434217000	0.019391000
H	5.717415000	3.657330000	0.012429000
H	4.934373000	3.175418000	-1.517453000
H	-0.674580000	-4.421454000	4.378553000
H	1.033206000	-2.959512000	3.324794000
H	-0.752564000	0.501907000	5.247250000
H	0.320647000	-3.889874000	-4.800142000
H	1.460081000	-3.373363000	-6.073413000
H	0.943196000	-2.218770000	-4.818177000
H	-1.323820000	-4.761339000	-2.312302000
H	-1.465318000	-6.521166000	-2.047332000
H	-0.413629000	-5.896601000	-3.343746000
H	2.356193000	-2.160846000	5.409724000
H	-1.724734000	5.973284000	-2.601788000
H	-2.711860000	6.308273000	-1.155576000
H	-2.294083000	4.625808000	-1.580779000
H	-6.607636000	2.622741000	-1.235774000
H	-6.543026000	2.420877000	-3.005615000
H	-5.030064000	2.484724000	-2.063105000
H	4.571827000	-2.933901000	6.210691000
H	-4.273039000	-0.235711000	-3.524765000
H	-5.870262000	-0.309556000	-4.311725000
H	-5.297735000	-1.683252000	-3.326806000
H	1.180747000	4.761340000	6.598145000
H	-2.437084000	-3.436005000	5.861067000
H	4.316700000	0.605292000	2.697740000
H	2.127679000	-7.450764000	-2.198373000
H	0.971450000	-8.167306000	-1.045521000
H	2.454537000	-7.383241000	-0.445571000
H	-2.477582000	-0.966018000	6.269206000
H	2.320529000	0.613038000	6.163787000
H	0.809154000	-5.563186000	1.444375000
H	-0.680179000	-6.384349000	0.900657000
H	-0.568020000	-4.601405000	0.839766000
H	-5.493187000	-0.729201000	3.878781000
H	-5.230441000	0.923265000	4.493944000
H	-3.910906000	0.065350000	3.649493000
H	6.672080000	-1.945437000	5.271619000
H	-3.755056000	2.664873000	1.794672000
H	-5.177852000	3.374280000	2.607983000
H	-5.172088000	3.172419000	0.834336000
H	5.501721000	-2.135064000	-1.444319000
H	6.181258000	-2.504346000	0.164855000
H	4.696357000	-1.523633000	0.028603000
H	4.036505000	-5.983460000	-3.875770000
H	3.533677000	-5.566460000	-5.534838000
H	2.357619000	-6.281612000	-4.404457000
H	-7.458648000	-4.048146000	1.488958000

H	-7.976745000	-2.613674000	0.565395000
H	-7.395219000	-2.434525000	2.242863000
H	3.953380000	-1.604916000	-3.943884000
H	4.363632000	-2.584397000	-5.376751000
H	5.092537000	-2.971190000	-3.796809000
H	4.159118000	4.114972000	-3.795770000
H	3.359529000	3.776156000	-5.352257000
H	3.225416000	2.608835000	-4.010803000
H	1.157533000	7.768338000	0.109164000
H	-0.505780000	8.303509000	-0.239999000
H	0.600978000	7.905849000	-1.580132000
H	4.716517000	-6.385450000	-0.949942000
H	6.165315000	-5.552414000	-0.327595000
H	5.603783000	-5.237635000	-1.988750000
H	0.593226000	2.755151000	2.809594000
H	0.401937000	4.795611000	4.217756000
H	6.526153000	-0.161812000	3.517575000
H	4.495706000	6.246092000	-2.006122000
H	5.149927000	6.648620000	-0.398561000
H	3.534166000	7.234502000	-0.872320000
H	-1.173455000	4.389982000	1.435107000
H	-1.558996000	6.115538000	1.691736000
H	0.102416000	5.521708000	1.974713000
H	2.145111000	2.658216000	7.558262000
H	-7.923372000	1.627597000	1.179405000
H	-7.815290000	1.755233000	2.953178000
H	-8.018746000	0.157951000	2.186964000
H	-4.207483000	-3.736582000	-1.332521000
H	-5.948882000	-3.578404000	-1.689707000
H	-5.367989000	-4.920369000	-0.671528000
H	2.602412000	5.933469000	1.876022000
H	4.224787000	5.253928000	2.156698000
H	2.796260000	4.185592000	2.175231000
H	0.112652000	2.813196000	-4.616248000
H	0.308620000	4.182121000	-5.740219000
H	-0.771269000	4.334644000	-4.327096000
H	-4.471539000	-4.470684000	2.258950000
H	-4.330114000	-2.824999000	2.938579000
H	-3.197277000	-3.363751000	1.673859000
H	0.892515000	6.985089000	-3.862191000
H	2.062676000	6.575497000	-5.140453000
H	2.637956000	6.931404000	-3.490484000
H	3.050782000	0.398883000	0.863736000
H	2.198221000	-1.310768000	1.227067000
H	2.398815000	1.367143000	1.277821000

2b_H_H
SCF = -1973.62913735
H(0 K) = -1972.344607
H(298 K) = -1972.220967
G(298 K) = -1972.523816
SCF+D3 = -1973.94716296
PCM SCF (Benzene) = -1973.65936784
BS2 (def2-tzvp) = -24397.4609980
Low Freq. = 3.8840cm-1, 5.8495cm-1

C	3.459667000	3.360815000	5.680269000
C	2.178853000	3.704457000	6.138105000
C	1.049359000	3.070242000	5.592681000
C	1.200847000	2.105009000	4.586526000
C	2.487816000	1.754370000	4.121951000

C	3.618104000	2.385147000	4.680827000
P	2.654601000	0.405646000	2.841035000
C	4.462937000	0.469849000	2.396205000
C	5.382969000	-0.456576000	2.929781000
C	6.753057000	-0.335312000	2.641106000
C	7.216304000	0.711631000	1.829876000
C	6.304108000	1.639474000	1.298609000
C	4.934263000	1.516277000	1.573344000
Rh	1.100790000	0.713400000	1.080277000
Ge	2.203066000	1.031273000	-1.484373000
Ge	1.201733000	-1.339247000	-0.977330000
Si	2.604474000	-3.232639000	-1.902853000
Si	4.896246000	-2.987598000	-1.187398000
C	6.025391000	-4.007955000	-2.347014000
Ge	-0.206781000	2.068638000	-0.921686000
Si	-0.240227000	4.546318000	-1.313493000
Si	0.896219000	4.991059000	-3.393344000
C	0.498126000	6.789545000	-3.909097000
Ge	-1.568440000	1.296633000	1.192133000
Ge	-2.664968000	-0.860752000	0.162401000
Si	-4.836666000	-1.750491000	0.954278000
Si	-6.397628000	0.084911000	0.904055000
C	-8.145019000	-0.611422000	1.244244000
Ge	-0.040572000	0.231230000	-3.000585000
Ge	-2.412187000	0.815126000	-1.861692000
Ge	-0.370099000	-1.731054000	1.116777000
Ge	-1.297018000	-1.869310000	-1.869248000
C	2.494419000	-1.144110000	3.864146000
C	2.386330000	-2.396341000	3.221804000
C	2.286922000	-3.574688000	3.977973000
C	2.288419000	-3.515229000	5.381301000
C	2.398011000	-2.273846000	6.025929000
C	2.503349000	-1.091690000	5.274235000
Si	-2.550351000	5.227194000	-1.447082000
C	-3.587227000	4.458293000	-0.038373000
Si	0.849565000	5.659348000	0.522932000
C	-0.218218000	5.551836000	2.102622000
Si	2.547993000	-3.093825000	-4.318259000
C	3.528120000	-1.569006000	-4.922135000
Si	1.732494000	-5.361422000	-1.191523000
C	1.440456000	-5.384965000	0.697068000
Si	-5.484969000	-3.554333000	-0.505914000
C	-4.052945000	-4.793086000	-0.742583000
Si	-4.412492000	-2.493485000	3.208541000
C	-3.387601000	-1.203383000	4.177095000
C	0.782451000	-3.016584000	-5.042409000
C	3.361712000	-4.680535000	-5.013015000
C	0.099048000	-5.787186000	-2.083083000
C	3.024385000	-6.712195000	-1.599560000
C	5.148278000	-3.644902000	0.589240000
C	5.450911000	-1.164550000	-1.269743000
C	-6.962024000	-4.458080000	0.303609000
C	-6.013448000	-2.877068000	-2.210503000
C	-3.314408000	4.725499000	-3.123492000
C	-2.629462000	7.130834000	-1.277079000
C	2.566214000	4.908529000	0.893239000
C	1.091929000	7.496581000	0.050443000
C	2.787071000	4.811608000	-3.188062000
C	0.306608000	3.820868000	-4.781436000
C	-6.092347000	-2.735010000	4.088560000
C	-3.462088000	-4.150140000	3.208840000

C	-5.959525000	1.365104000	2.252493000
C	-6.402689000	0.942825000	-0.800615000
H	-6.678655000	-4.917446000	1.265140000
H	-7.312308000	-5.265880000	-0.363404000
H	-7.811542000	-3.780058000	0.486532000
H	4.545382000	-3.090495000	1.326399000
H	6.209524000	-3.536214000	0.875522000
H	4.889823000	-4.714260000	0.666066000
H	2.461741000	3.886000000	1.291200000
H	3.083602000	5.519994000	1.653786000
H	3.199898000	4.872457000	-0.008367000
H	2.197082000	-4.537537000	3.466182000
H	2.379966000	-2.452169000	2.127933000
H	2.592187000	-0.131241000	5.788824000
H	0.137515000	-3.819119000	-4.648313000
H	0.837853000	-3.132966000	-6.139809000
H	0.290856000	-2.051567000	-4.834025000
H	-0.676331000	-5.021915000	-1.908071000
H	-0.289212000	-6.753937000	-1.716167000
H	0.246633000	-5.876299000	-3.172180000
H	5.035560000	-1.266061000	3.577871000
H	-2.790856000	5.203922000	-3.967776000
H	-4.372619000	5.039520000	-3.163257000
H	-3.280946000	3.633001000	-3.274512000
H	-6.892607000	-2.216515000	-2.130922000
H	-6.282575000	-3.714566000	-2.878427000
H	-5.200869000	-2.309157000	-2.693610000
H	7.457851000	-1.060130000	3.060859000
H	-3.199531000	-4.320330000	-1.258172000
H	-4.392987000	-5.642645000	-1.361083000
H	-3.693449000	-5.194031000	0.219055000
H	2.059230000	4.463336000	6.917771000
H	2.203955000	-4.434581000	5.969326000
H	4.233718000	2.248725000	1.158044000
H	3.264301000	-6.743120000	-2.674588000
H	2.625339000	-7.701940000	-1.314244000
H	3.964455000	-6.557385000	-1.044545000
H	2.402389000	-2.218728000	7.119195000
H	4.622559000	2.118343000	4.340805000
H	2.387416000	-5.205304000	1.233366000
H	1.049945000	-6.368873000	1.011931000
H	0.714248000	-4.614250000	1.009835000
H	-6.020101000	0.927103000	3.262673000
H	-6.668207000	2.211287000	2.210957000
H	-4.942485000	1.770152000	2.115388000
H	8.284759000	0.806518000	1.611878000
H	-5.423110000	1.393823000	-1.033549000
H	-7.157927000	1.748782000	-0.811164000
H	-6.649632000	0.236970000	-1.610441000
H	5.297952000	-0.740055000	-2.275903000
H	6.525348000	-1.086254000	-1.026958000
H	4.901108000	-0.541821000	-0.544579000
H	4.376953000	-4.848784000	-4.621505000
H	3.432163000	-4.594158000	-6.112260000
H	2.753158000	-5.572427000	-4.788978000
H	-5.925715000	-3.134125000	5.104934000
H	-6.739761000	-3.446020000	3.549448000
H	-6.639101000	-1.782670000	4.187904000
H	3.097895000	-0.630669000	-4.532283000
H	3.500064000	-1.517808000	-6.025088000
H	4.585959000	-1.613683000	-4.614418000

H	3.187544000	5.538932000	-2.462364000
H	3.287907000	4.991206000	-4.156039000
H	3.063939000	3.798593000	-2.848189000
H	-2.300370000	7.463852000	-0.278791000
H	-3.671899000	7.468764000	-1.416221000
H	-2.007552000	7.640058000	-2.031170000
H	5.729795000	-5.068821000	-2.391094000
H	7.063087000	-3.963576000	-1.970150000
H	6.026468000	-3.607274000	-3.373895000
H	0.314670000	1.621020000	4.162944000
H	0.046826000	3.329703000	5.947577000
H	6.657615000	2.459581000	0.665764000
H	1.788350000	7.605883000	-0.797376000
H	1.522489000	8.041993000	0.909310000
H	0.143554000	7.987663000	-0.220290000
H	-3.642324000	3.359943000	-0.135738000
H	-4.619599000	4.849157000	-0.075316000
H	-3.168558000	4.694908000	0.953654000
H	4.344445000	3.848763000	6.101450000
H	-8.470215000	-1.294349000	0.441959000
H	-8.871121000	0.219713000	1.290822000
H	-8.195544000	-1.157688000	2.200128000
H	-2.496926000	-4.061311000	2.681964000
H	-4.043566000	-4.954499000	2.728751000
H	-3.252327000	-4.461236000	4.247779000
H	-1.195043000	6.045503000	1.968841000
H	0.299652000	6.048793000	2.942064000
H	-0.399529000	4.502520000	2.391369000
H	0.573032000	2.770752000	-4.570980000
H	0.786143000	4.103969000	-5.735434000
H	-0.785361000	3.871660000	-4.922841000
H	-3.274813000	-1.527534000	5.227076000
H	-3.868402000	-0.211420000	4.175400000
H	-2.377011000	-1.095982000	3.746844000
H	-0.575637000	6.923262000	-4.121111000
H	1.053771000	7.037464000	-4.831137000
H	0.787679000	7.518949000	-3.135222000
H	0.791132000	2.032638000	1.845043000
H	2.290448000	1.648529000	0.523482000
H	0.061615000	0.008173000	2.165531000

5. References

[1] J. Cosier and A. M. Glazer, *J. Appl. Crystallogr.*, 1986, **19**, 105−107.

[2] *CrysAlisPro*, Agilent Technologies, Version 1.171.35.8.

[3] a) G. M. Sheldrick in SHELXL97, *Programs for Crystal Structure Analysis (Release 97-2)*, Institut für Anorganische Chemie der Universität, Tammanstrasse 4, D-3400 Göttingen, Germany, 1998; b) G. M. Sheldrick, *Acta Crystallogr. Sect. A*, 1990, **46**, 467−473; c) G. M. Sheldrick, *Acta*

Crystallogr. Sect. A, 2008, **64**, 112−122. d) Hubschle, C. B.; Sheldrick, G. M.; Dittrich, B. *J. Appl. Crystallogr.*, 2011, **44**, 1281−1284.

[4] Gaussian 16, Revision A.03, M. J. Frisch, G. W. Trucks, H. B. Schlegel, G. E. Scuseria, M. A. Robb, J. R. Cheeseman, G. Scalmani, V. Barone, G. A. Petersson, H. Nakatsuji, X. Li, M. Caricato, A. V. Marenich, J. Bloino, B. G. Janesko, R. Gomperts, B. Mennucci, H. P. Hratchian, J. V. Ortiz, A. F. Izmaylov, J. L. Sonnenberg, D. Williams-Young, F. Ding, F. Lipparini, F. Egidi, J. Goings, B. Peng, A. Petrone, T. Henderson, D. Ranasinghe, V. G. Zakrzewski, J. Gao, N. Rega, G. Zheng, W. Liang, M. Hada, M. Ehara, K. Toyota, R. Fukuda, J. Hasegawa, M. Ishida, T. Nakajima, Y. Honda, O. Kitao, H. Nakai, T. Vreven, K. Throssell, J. A. Montgomery, Jr., J. E. Peralta, F. Ogliaro, M. J. Bearpark, J. J. Heyd, E. N. Brothers, K. N. Kudin, V. N. Staroverov, T. A. Keith, R. Kobayashi, J. Normand, K. Raghavachari, A. P. Rendell, J. C. Burant, S. S. Iyengar, J. Tomasi, M. Cossi, J. M. Millam, M. Klene, C. Adamo, R. Cammi, J. W. Ochterski, R. L. Martin, K. Morokuma, O. Farkas, J. B. Foresman and D. J. Fox, Gaussian, Inc., Wallingford CT, 2016.

[5] A. D. Becke *Phys. Rev. A*, 1998, **38***,* 3098.

[6] J. P. Perdew *Phys. Rev. B*, 1986, **33***,* 8822.

[7] D. Andrae, U. Häußermann, M. Dolg, H. Stoll and H. Preuß *Theor. Chim. Acta.*, 1990, **77***,* 123.

[8] W. Ehlers, M. Böhme, S. Dapprich, A. Gobbi, A. Höllwarth, V. Jonas, K. F. Köhler, R. Stegmann, A.Veldkamp, G. Frenking *Chem. Phys. Lett.*, 1993, **208***,* 111.

[9] A. Höllwarth, M. Böhme, S. Dapprich, A. W. Ehlers, A. Gobbi, V. Jonas, K. F. Köhler, R. Stegmann, A. Veldkamp and G. Frenking *Chem. Phys. Lett.*, 1993, **208***,* 237.

[10] W. J. Hehre, R. Ditchfield and J. A. Pople *J. Chem. Phys.*, 1972, **56***,* 2257.

[11] P. C. Hariharan and J. A. Pople *Theor. Chim. Acta.*, 1973, **28***,* 213.

[12] F. Weigend and R. Ahlrichs *Phys. Chem. Chem. Phys.*, 2005, **7***,* 3297.

[13] D. Andrae, U. Häußermann, M. Dolg, H. Stoll and H. Preuß *Theor. Chim. Acta*, 1990, **77**, 123−141.

[14] S. Grimme, J. Antony, S. Ehrlich and H. Krieg *J. Chem. Phys.*, 2010, **132**, 154104.
